# Abstracts of scientific papers and sessions, CPA Congress 2011

**DOI:** 10.3138/physio.63.supp

**Published:** 2011-07

**Purpose/Objectives & Rationale:** To assess physiotherapists' perceptions about factors influencing their ability to read journal articles.

**Relevance to Physiotherapy Practice:** At the WCPT congress in 2007, a number of presenters concluded that clinicians were not aware of or practicing as per the latest published clinical guidelines or not aware of the latest research. Prior to September 2007, physiotherapists working only in private clinics (at least 50% of physiotherapists practicing in BC) did not have access to full-text articles. We hypothesized that access was a primary barrier to reading articles, potentially influencing the uptake of research findings.

**Materials and Methods:** A survey was sent by postal mail to 200 randomly selected members of the Physiotherapy Association of BC (PABC), prior to PABC purchasing access for all members to the Electronic Health Library of BC. The same survey was readministered one year later.

**Analysis:** Descriptive statistics were used to assess perceived barriers at baseline and one year.

**Results:** Thirty-four percent (68/200) of participants responded at both baseline and one year. Time was the most important factor (86% of respondents at baseline; 88% at one year). Access to full text was important (45% of respondents at baseline, 32% at one year). Access to databases was important for 21% at baseline and 12% at one year. At one year the percentage of respondents who perceived ‘restricted access to a specific journal’ and their ‘ability to understand articles’ as important barriers increased.

**Conclusions:** ‘Time’, ‘access to full text’ and ‘access to databases’ were identified as the three most important factors influencing respondents' ability to read journal articles.
