# Supplementary material for: Abstracts of scientific papers and sessions, CPA Congress 2011
Source: Physiother Can. 2011 Jul;63(Suppl 1):1–82. doi: 10.3138/physio.63.supp (PMC3286878; doi:10.3138/physio.63.supp)
Supplement: Related texts

# Abstract: A011 – A SYSTEMATIC REVIEW OF PHYSIOTHERAPY MANAGEMENT OF PATIENTS FOLLOWING NECK DISECTION FOR CANCER

**Published:** 2011-07

**Authors:** TJ Overend, CM Anderson, DM Del Greco, RL Mathews, NK Potter, AX Zhao

**Affiliations:** * School of Physical Therapy, The University of Western Ontario; † London Health Sciences Centre, Physiotherapy, London, ON

**Purpose/Objectives & Rationale:** To systematically review the evidence related to the physiotherapy management of patients following neck dissection for cancer.

**Relevance to Physiotherapy Practice:** Historically, survival has been the focus for patients diagnosed with cancer of the head and neck. Improved therapies have increased the likelihood of survival and even cure for patients with these diagnoses.

**Materials and Methods:** Medline, PubMed, CINAHL, EMBASE, Google Scholar and Scopus electronic databases were searched from inception to September 2010. Each study was reviewed by two reviewers for inclusion. A secondary search was also performed. Four reviewers independently reviewed each paper before meeting to reach consensus on study quality (appraised using the Jadad scale and PEDro criteria).

**Analysis:** Meta-analysis was carried out on studies with similar methodology and outcome measures. Standard difference in means and 95% confidence intervals were calculated using a random effects model. Studies not included in the meta-analysis were assessed qualitatively.

**Results:** Two prospective cohort studies and three randomized control trials were identified. Meta-analysis indicated that progressive resistance exercises resulted in significant improvements in active forward flexion (*p* = 0.029) and external rotation (*p* < 0.001), passive forward flexion (*p* = 0.022) and the disability component of the Shoulder Pain and Disability Index (*p* = 0.035). Acupuncture was found to improve the Constant-Murley score (*p* = 0.008) and xerostomia (*p* = 0.02).

**Conclusions:** There is evidence that progressive resistance exercise following neck dissection increases range of motion and decreases shoulder disability. Acupuncture may also be effective in decreasing pain and dysfunction. More research is needed with this population to further our understanding of the optimal physiotherapy management of these patients.

# Abstract: A013 – CAN A PHYSIOTHERAPY EXERCISE PRESCRIPTION CHANGE FATIGUE AND LOW ACTIVITY LEVELS IN YOUNG ADULTS UNDERGOING AGGRESSIVE TREATMENT FOR CANCER?

**Published:** 2011-07

**Authors:** MA Dalzell, S Shallwani, S Adams, T Muanza, R Dalfen, M Karanofsky, P Kavan

**Affiliations:** McGill Adolescent and Young Adult Oncology Program, Jewish General Hospital - Segal Cancer Centre, Montreal

**Purpose/Objectives & Rationale:** To evaluate compliance, changes in activity levels and fatigue in young adults (YA) with cancer who receive active lifestyle coaching and exercise prescription during treatment.

**Relevance to Physiotherapy Practice:** The aggressive nature of YA cancers and treatment results in significant fatigue and functional limitations. While benefits of exercise post-treatment in breast and prostate cancer populations are clear, little evidence on the compliance, risks and benefits during treatment in diverse cancer populations is available to clinicians.

**Materials and Methods:** Ninety-seven patients, aged 18 to 45 years, with brain, gastrointestinal, breast, sarcoma, and testicular cancers were consecutively recruited and screened by a physiotherapist for risks related to exercise. Individualized programs were prescribed and conducted at home or at a community-based Wellness Centre. Metabolic equivalent hours of physical activity (MET-hours/week) and the Brief Fatigue Inventory (BFI), were documented at baseline (T1) and following an average of 6 months of treatment (T2).

**Analysis:** Paired *t-*tests were used to compare activity levels and fatigue at T1 and T2. Compliance was documented and independent *t-* tests used to compare exercisers and non-exercisers.

**Results:** Compliance with exercise prescription was 71% and subjects increased their activity levels from 8.2 to 18.6 MET-hours/week (*p* < 0.01) with no adverse effects. BFI severity and impact scores remained stable (*p* > 0.05). Exercisers had significantly higher activity levels and lower fatigue than non-exercisers at T2 (*p* < 0.05).

**Conclusions:** Patients with YA cancers can safely exercise and benefit from exercise prescription during aggressive treatment. Physiotherapists play a significant role in determining risks and providing exercise prescription to reduce functional morbidity.

# Abstract: A017 – HAND AND UPPER EXTREMITY FUNCTION IN WORKERS WITH HAND DERMATITIS: WHAT HAVE WE LEARNED SO FAR?

**Published:** 2011-07

**Authors:** S Switzer-McIntyre, D Beaton, R Nixon, E Harniman, DL Holness

**Affiliations:** * University of Toronto, Physical Therapy, Toronto; † St. Michael's Hospital, Department of Occupational & Environmental Health, Toronto; ‡ Universtiy of Toronto, Occupational Therapy, Toronto; § St. Michael's Hospital, Mobility Program Clinical Research Unit, Toronto and Occupational Dermatology Research and Education Centre, Victoria, Australia; ** University of Toronto, Departments of Medicine & Dalla Lana School of Public Health, Toronto

**Purpose/Objectives & Rationale:** The overall purpose of this study is to gain a better understanding of the functional compromise of the hand and upper extremity that may exist for workers suffering from contact dermatitis of the hand. Hand dermatitis (HD) is a common disease which is often due to occupational exposures. Traditional methods of assessing impairment for skin conditions focus on clinical severity, medication usage, activities of daily living and quality of life. Assessment of upper extremity function may provide important information that could enhance the overall management of this patient population.

**Relevance to Physiotherapy Practice:** Currently very few HD patients are referred to physiotherapy for rehabilitation of their hands and upper extremity. This study aimed to explore this patient population to begin to understand the role physiotherapy could play in their return to optimal function.

**Materials and Methods:** Data was obtained for 62 participants through a musculoskeletal examination and a patient-reported questionnaire.

**Analysis:** Descriptive statistics including frequency distributions, means, standard deviations, and ranges were used to analyze the data.

**Results:** Finger joint restrictions and numbness were moderate-to-severe in 30.0% and 28.6%, respectively. Positive Tinel's and/or Phalen's Tests occurred in 24.6%. 15.8% had a SF-36 Mental Component Summary score of < 30. Mean QuickDASH score was 30.2. 47.5% reported moderate-to-high work instability and 31.4% reported a > 10% decrease in productivity. Worse UE function and mental health were associated with reduced productivity (*r* = 75.6%).

**Conclusions:** Hand dermatitis is associated with compromised UE function, decreased mental health, and ability to work. Assessment should include more than the severity of skin changes.

# Abstract: A029 – REHABILITATION OUTCOMES BASED ON STRAIGHT LEG RAISE FINDINGS

**Published:** 2011-07

**Authors:** SS Rege

**Affiliations:** CBI Health Centre Moncton, Moncton, NB

**Purpose/Objectives & Rationale:** The straight leg raise (SLR) test is a common component of a clinician's low back pain (LBP) physical examination. This study compares the rehabilitation outcomes of those with (*n* = 343) and those without (*n* = 1853) a positive SLR test.

**Relevance to Physiotherapy Practice:** If neurological deficit does not directly interfere with job demands, return to work can be an achievable goal even for those with a positive SLR finding.

**Materials and Methods:** This was a prospective observational cohort study of 2196 LBP cases treated non-operatively at 40 spine care clinics. Positive SLR findings were cross referenced with location of dominant pain to create 3 groups: False Positives (FP), True Positives (TP) and True Negatives (TN). Outcomes assessed were: change in perceived function, change in pain rating, change in medication usage, total treatment days and return to work rates.

**Analysis:** Analysis of variance was used to assess statistical differences between groups.

**Results:** The TP group had significantly more pain, more medication usage, treatment days and less functional improvement at treatment conclusion and at 3-month follow-up than the FP and TN groups (*p* < 0.05). For the FP and TN groups, there were no statistically significant differences in medication use and functional improvement at follow up. The TN Group had the highest return to work rate with a trend towards statistical significance (*p* < 0.075).

**Conclusions:** A positive SLR was over diagnosed and a true positive SLR test was a rare clinical finding. In spite of slower treatment response, higher medication use and less pain reduction in the TP group, they had comparable return to work rates.

# Abstract: A033 – CAN VASTUS MEDIALIS OBLIQUUS BE PREFERENTIALLY ACTIVATED IN PATIENTS WITH PATELLOFEMORAL PAIN SYNDROME?

**Published:** 2011-07

**Authors:** KD Obright

**Affiliations:** The School of Physical Therapy, Elborn College, The University of Western Ontario, London, ON

**Purpose/Objectives & Rationale:** To review the evidence to determine if vastus medialis obliquus (VMO) could be preferentially activated by lower limb joint position or co-contraction.

**Relevance to Physiotherapy Practice:** Patellofemoral pain syndrome (PFPS) is one of the most prevalent conditions of the knee commonly treated by physiotherapists. Rehabilitation protocols commonly incorporate exercises postulated to selectively strengthen VMO. Utilizing the best available evidence will help therapists select the most beneficial exercises for their clients.

**Materials and Methods:** A literature search was conducted for the years 1980-2010 using CINAHL, Medline, PubMed and PEDRO electronic databases. Keywords included patellofemoral pain syndrome, vastus medialis obliquus, quadriceps, electromyography and exercises.

**Analysis:** Sixty-six articles were reviewed. Seven articles met the inclusion criteria. The Critical Appraisal Skills Programme tool was then used to evaluate methodological quality of each selected article.

**Results:** Seven randomized or clinical controlled trials were identified. One study assessed the influence of lower limb co-contraction, four studies evaluated the effects of altering lower limb orientation and two studies investigated both. Three of these studies suggested that VMO may be preferentially activated by performing quadriceps exercises, however, serious methodological errors meant that their results must be viewed with caution.

**Conclusions:** Research supporting the preferential recruitment of VMO relative to VL using various methods indicates that VMO cannot be preferentially activated by altering lower limb position or through the addition of co-contraction. The evidence demonstrated generalized quadriceps strengthening, but no specific VMO effect. Physiotherapists should, therefore, not focus on selectively retraining vastus medialis obliquus as traditionally recommended, but rather focus on generalized quadriceps exercises to increase VMO activity.

# Abstract: A038 – COLLABORATIVE COACHING SOLUTIONS FOR SPORT-PARTICIPATION OF CHILDREN WITH DISABILITIES: EXTENDED EDUCATIONAL ROLE FOR PAEDIATRIC THERAPISTS

**Published:** 2011-07

**Authors:** L Pelland, V James

**Affiliations:** School of Rehabilitation Therapy, Queen's University, Kingston, ON

**Purpose/Objectives & Rationale:** This study examined the participation of children with disabilities in recreational soccer from the perspective of coaches, a contrast to traditional models of inclusion that focus on family and child related factors. Our aim was to document the knowledge, attitudes and beliefs of recreational soccer coaches, in a small rural community, on the participation of children with disabilities on their teams.

**Relevance to Physiotherapy Practice:** Developing coaches' skill in adopting family-centered principles in their practice could improve the enjoyment of children with disabilities, and their families, in community-based sports; paediatric physiotherapists are well positioned to assume a leadership role in the development of this curriculum.

**Materials and Methods:** Mixed methods, combining semi-structured focus group interviews and self-report questionnaire; eight coaches participated.

**Analysis:** Standard content analysis, with back translation, to create thematic categories; thematic content was qualitatively evaluated as positively or negatively influencing participation. Interviews were contextualized using ethnographic summarization.

**Results:** Six themes emerged: general knowledge and attitude toward disability, adaptation to child's needs, safety, collaboration with parents, preparation of teammates and coaching role. Positive factors on participation included attitudes and beliefs toward benefit of sport-participation for all children, confidence in team management and adoption of fair play rules. Low confidence in knowledge about disabilities, concerns around safety and low confidence in working collaboratively with parents negatively influenced participation. Coaches identified expert-led education session as the most effective information-sharing method.

**Conclusions:** Direct involvement of paediatric therapists with coaches could create innovative educational solutions to support the participation of children with disabilities in community-based sport.

# Abstract: A041 – A CASE STUDY OF PROGRESSIVE EXERCISE TO IMPROVE BALANCE AND MOBILITY THROUGH HOME-CARE PHYSIOTHERAPY

**Published:** 2011-07

**Authors:** M Earl, D Hollway, A Murphy, M MacKay-Lyons, E MacDonald

**Affiliations:** * School of Physiotherapy, Dalhousie University, Halifax; † St. Joseph's Hospital, Saint John, NB; ‡ Faculty of Medicine,Dalhousie University, Halifax

**Purpose/Objectives & Rationale:** Best-practice falls prevention guidelines support assessment and treatment of impairments that compromise balance. This case study illustrates prescription of high-intensity, progressive exercise to improve vestibular control of balance of a senior, who was referred for home care physiotherapy.

**Relevance to Physiotherapy Practice:** Impaired balance and mobility can threaten the independence of seniors who need home-care physiotherapy. More evidence is needed to guide exercise prescription for this vulnerable group.

**Materials and Methods:** An 84-year-old woman completed an 8-week, supervised, home exercise program, that included high-intensity, progressive resistive exercise to strengthen lower-limb muscles (3 sessions/week), plus balance exercise (2 sessions/week) to improve vestibular control. Objective measures were completed at baseline, on completion of the program, and again 6 months later. Measures included: the Clinical Test of Sensory Interaction for Balance (CTSIB), the Timed Up and Go (TUG), and the Activities Specific Balance Confidence Scale (ABC).

**Analysis:** Scatter plots and descriptive statistics were used to document changes in all measures.

**Results:** Baseline assessment revealed CTSIB < 5s (compliant surface; eyes open, eyes closed, and with visual conflict), TUG = 20s, and ABC = 20%. At completion of the program, her CTSIB = 30s for all subtests, TUG = 15s, and ABC = 50%. The improvements persisted after six months. She reported 5 falls in the year prior to the exercise; one fall was reported during the exercise and follow-up periods.

**Conclusions:** High intensity strength and balance exercises were feasible for this client. Her improvements in balance and mobility support further consideration of progressive exercise to restore and protect the balance and mobility of seniors who require home-care physiotherapy.

# Abstract: A047 – WHAT DOES “RECOVERY” FROM WHIPLASH MEAN TO KEY STAKEHOLDERS? A MIXED-METHODS STUDY

**Published:** 2011-07

**Authors:** DM Walton, ET Taylor

**Affiliations:** * The University of Western Ontario, School of Physical Therapy; † LifeMark Physiotherapy, London ON

**Purpose/Objectives & Rationale:** Estimates of recovery following whiplash are hampered by inconsistent definitions of the outcome. The purpose of this study was to elicit the perspectives of patients and clinicians on the meaning of 'recovery' following whiplash injury.

**Relevance to Physiotherapy Practice:** Physiotherapists are often asked to provide an estimate of prognosis following whiplash injury, but the evidence is conflicting for recovery rates (16-99% recovery reported), due in large part to differences in the operationalization of recovery. A more unified definition, that incorporates perspectives of all key stakeholders, is needed to facilitate interpretation of research and communication.

**Materials and Methods:** A mixed-methods approach to eliciting the meaning of recovery from patients (*n = *20) and clinicians (*n = *15) was used. Focus groups (nominal group technique) combined with individual interviews for greater depth were used.

**Analysis:** Vote tallying and thematic analyses were used to identify the important domains of recovery. Triangulation through multiple blinded coders increased confidence in the constructs identified.

**Results:** Both patients and clinicians recognize that reduced or eliminated pain is not a sufficient definition of recovery. Patients viewed recovery more eudaimonically, focusing on personal satisfaction, interpersonal relationships and potential for future achievement. Clinicians endorsed a number of validated self-report tools as well as patient opinion to define recovery. Both groups indicated that return to work could be misleading if not further investigated.

**Conclusions:** A definition of recovery that could better be defined as 'happiness', informed by at least 6 sub-constructs has been developed. Both patients and clinicians recognize that recovery requires more than pain reduction.

# Abstract: A051 – DOES RESPIRATORY MUSCLE TRAINING IMPROVE PERFORMANCE IN ATHLETES?

**Published:** 2011-07

**Authors:** WD Reid, B Ghanbari

**Affiliations:** Muscle Biophysics Laboratory, Department of Physical Therapy; A William Sheel, School of Human Kinetics, University of British Columbia, Vancouver

**Purpose/Objectives & Rationale:** Studies reporting evidence about the benefit of respiratory muscle training (RMT) for athletes is conflicting. A systematic review was performed to address the question – Does RMT improve sport performance, in addition to respiratory muscle strength and endurance?

**Relevance to Physiotherapy Practice:** Synthesis of the evidence about RMT for athletes will provide physiotherapists with a basis for exercise prescription.

**Materials and Methods:** Using the Cochrane protocol, MEDLINE, CINAHL, SPORTDiscus, PEDro, EMBASE, EBM, and COCHRANE databases were searched. Articles were included if (1) participants were adult athletes; (2) RMT was compared to sham/control; (3) they used a single group pre-post or randomized controlled trial (RCT), and reported outcomes of respiratory muscle strength and sport performance; (5) it was published in English. Quality assessment using PEDro and data abstraction was performed by two authors.

**Analysis:** Meta-analyses using RevMan 5.0.25 were calculated when outcomes and study design were similar.

**Results:** Of the 6,918 citations reviewed, 31 met the inclusion criteria and 25 were RCTs. Meta-analyses demonstrated an overall positive effect of RMT on sport performance (*p* = 0.02) and particularly, for rowers (*p* = 0.04). Ratings of perceived breathlessness (*p =* 0.004) and perceived exertion also were greater after RMT (*p =* 0.04). Meta-analyses showed greater improvement after RMT for maximal inspiratory pressures (<0.00001), maximal voluntary ventilation for 15 sec (*p =* 0.007) and maximum sustained voluntary ventilation for several minutes (*p* < 0.0001). Subgroup analyses revealed differences according to training device.

**Conclusions:** Larger sample sizes and closer matching of the RMT protocol (intensity, contraction velocity, range of motion, etc.) to the ventilatory demands of the athlete's sport may further eluciate whether RMT can improve performance.

# Abstract: A073 – PHYSICAL THERAPY MANAGEMENT OF VENTILATED PATIENTS WITH ACUTE RESPIRATORY DISTRESS SYNDROME OR SEVERE ACUTE LUNG INJURY

**Published:** 2011-07

**Authors:** F Chung, D Mueller

**Affiliations:** Burnaby Hospital Physiotherapy Department, Burnaby, BC

**Purpose/Objectives & Rationale:** To review the literature regarding physical therapy interventions for ventilated patients with acute respiratory distress syndrome or severe acute lung injury and to discuss limitations of this literature.

**Relevance to Physiotherapy Practice:** Patients with these severe respiratory conditions are often cared for by physical therapists in Canadian intensive care units but questions remain regarding best treatment practices.

**Materials and Methods:** A literature review was conducted in the spring and summer of 2009.

**Analysis:** A critical review of relevant articles published in English was completed. Statistical analysis was completed where appropriate and data were available.

**Results:** Some relevant articles are of low quality or have methodological problems which may lead to erroneous conclusions. Randomized control trials showed that earlier ICU mobilization is safe and is associated with positive outcomes. Systematic reviews comparing prone to supine position reported improved oxygenation, reduced risk of ventilator associated pneumonia and an increased risk of pressure ulcers in the prone position. A subgroup analysis showed a significant reduction in mortality in patients with higher illness severity in prone position. Systematic reviews comparing kinetic therapy to control reported that kinetic therapy decreased the incidence of nosocomial pneumonia but had no effect on mortality, duration of mechanical ventilation, or duration of ICU and hospital stays.

**Conclusions:** Stepwise early mobilization of ICU patients is safe and is associated with favourable outcomes in both hospital length of stay and functional ability of the patient. Early intervention with sufficient frequency, duration, and for adequate time periods is the key to success for many physiotherapy interventions for this type of patient.

# Abstract: A078 – SIMPLE THINGS MATTER! HOW PROPER PAIN MANAGEMENT STRATEGIES CAN IMPROVE OUTCOMES IN PHYSICAL THERAPY: A CASE REPORT

**Published:** 2011-07

**Authors:** G Leonard, Y Tousignant-Laflamme

**Affiliations:** École de réadaptation, Université de Sherbrooke, Sherbrooke, QC

**Purpose/Objectives & Rationale:** The objective of the present case report is to illustrate the influence of psychological factors on the rehabilitation process of patients suffering from pain.

**Relevance to Physiotherapy Practice:** Traditionally, psychological risk factors for chronic pain are seen as inherent patient characteristics that clinicians are asked to evaluate and modify. The present case report provides evidence that these psychological barriers to rehabilitation can also be involuntarily created and/ or perpetuated by clinicians themselves when too much attention is placed on the presumed source of pain.

**Materials and Methods:** We describe the case of VT, a 17-year-old female complaining of neck and back pain following a motor vehicle accident.

**Analysis:** We report the evolution of VT based on a series of validated and objective outcome measures: numerical pain scale (pain intensity), McGill Pain Questionnaire (pain quality), Pain Disability Index (perceived impact of pain on disability).

**Results:** After seven weeks of unsuccessful treatments, the condition of VT improved drastically when a physiotherapist reassured her about the findings of the physical exam, corrected her misbeliefs about pain, and brought her attention away from the physical “abnormalities” initially identified.

**Conclusions:** The present case report highlights the importance of addressing psychological factors when evaluating and treating people suffering from painful conditions, and warns physiotherapists against the potential drawbacks of according too much attention to the physical “abnormalities”.

# Abstract: A080 – A CLINICAL PRACTICE GUIDELINE FOR MOBILIZING PATIENTS WITH A DVT: TRANSLATING THEORY INTO CLINICAL PRACTICE IN THE ACUTE CARE SETTING

**Published:** 2011-07

**Authors:** C Singh, R Fletcher, K Cunningham, M Szvlika

**Affiliations:** * Surrey Memorial Hospital, Physiotherapy; † Eagle Ridge Hospital, Physiotherapy

**Purpose/Objectives & Rationale:** To standardize care and facilitate best practice in a busy acute care setting by developing and implementing a clinical practice guideline for mobilizing patients with a DVT.

**Relevance to Physiotherapy Practice:** The evidence base for physiotherapy is building, but this evidence is not consistently being transferred to and implemented by the front-line physiotherapist. To provide best patient care, we need to find ways to translate this evidence into practice in busy, acute settings.

**Materials and Methods:** After a CPA teleconference on this topic, a survey was done in our region and found that physiotherapists had 8 different practices for mobilizing patients with a DVT. Many were aware of the latest research but did not know how to apply it in their settings. A group of front-line clinicians developed a practice guideline and followed the Fraser Health Authority's clinical decision tool process which involved identifying stakeholders and a rigorous review of the guideline. The next step was to disseminate the guideline to over 200 physiotherapists and implement it at the individual sites. Six months after implementation, a survey was done to see if there was a practice change.

**Analysis:** Pre and post implementation surveys were reviewed to show evidence of practice change.

**Results:** There was less variation in physiotherapist's practice post-implementation and physiotherapists were using the document clinically to guide their practice.

**Conclusions:** It is important to engage front-line clinicians to change actual practice. A careful approach with new evidence (such as DVT and mobility) can facilitate practice change.

# Abstract: A083 – ACHILLES TENDINOPATHY: WHAT IS THE BEST TREATMENT?

**Published:** 2011-07

**Authors:** A Scott, E Huisman

**Affiliations:** Department of Physical Therapy, University of British Columbia, Vancouver

**Purpose/Objectives & Rationale:** Achilles tendinopathy is a chronic, activity-limiting syndrome. Many treatments have been advocated, but few directly address the underlying pathology — tendinosis. The purpose of this study was to review and summarize the evidence base for treating Achilles tendinopathy.

**Relevance to Physiotherapy Practice:** Many patients are drawn to oral or injection therapies as alternatives or adjunts to physiotherapy.

**Materials and Methods:** MEDLINE (1950 to August 2010) was searched using a string of terms related to Achilles tendinopathy. The Cochrane Collaboration Library was searched for systematic reviews of treatments for Achilles tendinopathy. Abstracts of identified studies were screened against inclusion criteria. Study quality was assessed using the 5 point Oxford Scale.

**Analysis:** Due to the small number of trials, a meta-analysis was not conducted.

**Results:** The search strategy identified 72 RCTs of which 14 qualified. A total of 777 patients (male:female ratio 0.51) were enrolled of average age 45.6 years, ranging from 18 to late 70s. Symptom duration ranged from 3 months to several years. The most common outcome measures were pain at rest or with activity. There was moderate evidence of clinically meaningful, long term reductions in pain and improved function with eccentric exercise. There was weak evidence of short term pain relief with orthotics. There was moderate to weak evidence of a lack of effect of high energy shockwave therapy, oral therapy including NSAIDs, or injections of corticosteroid or platelet-rich plasma.

**Conclusions:** Active exercise remains the best supported treatment for Achilles tendinopathy, positioning physiotherapists as leaders in the treatment of this widespread condition.

# Abstract: A086 – REALIST REVIEW OF KNOWLEDGE TRANSLATION STRATEGIES FOR GETTING OLDER ADULTS' FALLS PREVENTION EXERCISE PROGRAMS INTO THE COMMUNITY

**Published:** 2011-07

**Authors:** MC Ashe, E Gorman, C Leia, KM Khan, J Sims-Gould, AM Chudyk, H McKay, Allan Best

**Affiliations:** * Centre for Hip Health and Mobility, University of British Columbia; † Vancouver Coastal Health Authority; ‡ Centre for Clinical Epidemiology and Evaluation, University of British Columbia, Vancouver

**Purpose/Objectives & Rationale:** Literature highlights effective exercise programs for reducing falls in older adults. The objective of this study was to undertake a knowledge synthesis of available evidence on what facilitates knowledge translation (KT) of evidence-based programs for falls prevention into the community-setting.

**Relevance to Physiotherapy Practice:** Physiotherapists working in research or clinical practice who are involved in development, testing, KT and referral to exercise programs for falls prevention.

**Materials and Methods:** We used a realist review approach to synthesize knowledge on getting evidence-based exercise programs for falls prevention into the community. There were three main components: i) review of existing literature on how evidence-based falls prevention programs were disseminated into the community; ii) expert opinion to synthesize findings and define next steps; and iii) older adults' opinions using focus groups and surveys on what facilitates uptake and adherence to these programs.

**Analysis:** This was a mixed-methods synthesis, and the information was obtained from a standard systematic review, focus groups, surveys and synthesis from an expert panel.

**Results:** From the literature there were few studies that reported underlying theories and/or tested dissemination and implementation strategies for falls prevention exercise programs embedded within the community. Main themes that emerged included: available resources within the community, recruiting older adults into programs, and the importance of instructors and specific program elements for this population (e.g. individualized exercise, group-based).

**Conclusions:** Evidence-based exercise programs are an important component of falls prevention. This study highlights the need to test integrative KT strategies with relevant stakeholders for sustainable exercise programs for older adults.

# Abstract: A087 – ARE THE SAFE QUESTIONNAIRE AND THE ABC-S SCALE SUITABLE TO ASSESS A FRAIL, SENIOR POPULATION?

**Published:** 2011-07

**Authors:** S Hamel, V Tremblay-Boudreault, M Tousignant, H Corriveau

**Affiliations:** Research Centre on Aging, Sherbrooke Geriatric University Institute, Faculty of Medicine, University of Sherbrooke, Sherbrooke, QC

**Purpose/Objectives & Rationale:** The goal of this study was to determine which of the SAFE and ABC-S questionnaire was more suitable to assess fear of falling in a frail senior population.

**Relevance to Physiotherapy Practice:** Fear of falling assessment is recommended in guidelines regarding fall prevention. As those questionnaires have different theoretical definitions, it is difficult for clinicians to choose the right one. This study can help make a choice with respect to which questionnaire should be used in a frail population.

**Materials and Methods:** This study was conducted as a secondary analysis from a RCT. Participants were over 65 years old admitted on a day hospital program (*n =* 77). They were assessed by trained research agents on balance confidence, fear of falling, balance, gait velocity, mobility, dual task execution, activity restriction, functional autonomy, general self-efficacy, number of comorbidities and fall status. Follow-up concerning fall status was conducted for one year post-intervention through fall calendar and phone calls.

**Analysis:** Correlations between the different clinical variables and the ABS-S and SAFE scores were performed using either a Pearson or a point-biserial correlation coefficients according to the type of variable.

**Results:** Correlations between ABC-S score and clinical variables varied between -0.32 and 0.40 (*p = *0.05). Correlations between SAFE score and clinical variables varied between -0.25 and 0.43 and were not consistent. Neither of these tests could predict the fall status.

**Conclusions:** The ABC-S scale was more consistent and more related to clinical balance variables than the SAFE questionnaire. This suggests that it might be a better tool to assess fear of falling in a frail, senior population.

# Abstract: A089 – REQUISITE LENGTH OF PHYSIOTHERAPY INTERVENTION TO IMPROVE CLINICAL CHARACTERISTICS OF BALANCE AND GAIT IN ELDERLY

**Published:** 2011-07

**Authors:** H Corriveau, M Tousignant, PM Roy, V Tremblay-Boudreault, AJ Beaudoin, S Hamel, J Simard

**Affiliations:** Research Center on Aging, Sherbrooke Geriatric University Institute, Faculty of Medicine and Health Science, University of Sherbrooke, Sherbrooke, QC

**Purpose/Objectives & Rationale:** The objective of this study is to determine the required duration (12 or 15 weeks) of physical therapy to improve balance and gait characteristics in elderly.

**Relevance to Physiotherapy Practice:** To prevent falls among the elderly, physiotherapy interventions including progressive balance exercises and gait training have proven their effectiveness. However, it is still common to discharge patients from rehabilitation facilities after short stays. To our knowledge, no study has investigated the effective length of physiotherapy programs needed in order to observe improvement in falling outcomes.

**Materials and Methods:** Patients admitted to a day hospital program at the Sherbrooke University Geriatric Institute, Quebec, Canada were recruited for the study. The physiotherapy intervention was given twice a week (1 hour/session) for 15 weeks. Evaluation was completed at baseline and after 12- and 15-weeks of intervention. Balance was assessed with the Berg scale, and gait with the Time Up and Go test and the 5-meters walking time.

**Analysis:** Paired *t-*tests have been done subsequently to determine the time needed for improvement in each clinical variable. A Bonferroni correction has been applied to the *p-*value significant threshold (*p =* 0.0167).

**Results:** Seventy-six subjects (80.68 ± 5.97 years) participated in the progressive physiotherapy program. After 12 weeks, the participants improved on the Berg (*p =* 0.000), the Time Up and Go test (*p = *0.003) and the 5-meters walking time (*p =* 0.015). There were no significant differences between the improvement at 12 and 15 weeks of intervention for each variable.

**Conclusions:** The above findings indicate that a 12-week physiotherapy exercise program is sufficient to improve balance and gait control.

# Abstract: S003 – EFFECT OF A COMPREHENSIVE TRIAGE SYSTEM ON ACCESS TO SPINE SURGICAL SERVICES

**Published:** 2011-07

**Authors:** G Schneider, K Thomas, P Salo, P Faris, M Benour, University of Calgary Spine Program

**Affiliations:** University of Calgary, Faculty of Medicine, Graduate Sciences Education, Medical Sciences, Departments of Surgery and Clinical Neurosciences

**Purpose/Objectives & Rationale:** Timely access to surgical consultation for patients with spinal dysfunction in Canada is generally poor. We examined the effect of a triage system on access to spine surgical services.

**Relevance to Physiotherapy Practice:** Physiotherapist's expertise allows them to be a valued member of a triage team facilitating appropriate referrals for operative and non-operative spine care.

**Materials and Methods:** In this retrospective cohort study, the primary outcome was time from physician referral of patients with spinal dysfunction to surgical consultation and surgery. A random sample of patients was selected from the year prior to (*n =* 68) and following (*n =* 94) the inception of a triage system. Data was retrieved from patient files and electronic medical records.

**Analysis:** 'Descriptive analyses’ were used to summarize patient referrals. Estimation methods (mean, 95% CI) were used to compare the groups with respect to time (days) to surgical consultation and spine surgery from referral.

**Results:** Logarithmic transformation was used, as the data was not normally distributed. The geometric mean (95% CI) days to surgical consultation was 78 (55-112) and 74 (59-94); and to surgery was 206 (166-256) and 205 (177-237) pre and post triage respectively. The triage team assessed 3300 patients of whom 53% were referred for surgical consultation and the remainder referred for timely evidence-based rehabilitation and pain management. Surgeons noted a higher rate of appropriate surgical referrals post triage.

**Conclusions:** Wait-times for surgical consultation and surgery were unaffected by the initiation of a triage system. Future studies examining cost-effectiveness, acceptability, and functional outcomes are needed.

# Abstract: A001 – BRIDGING THE GAP: MScPT STUDENT VIDEO-CONFERENCES ENHANCE LEARNING ON CLINICAL PLACEMENT

**Published:** 2011-07

**Authors:** TL Parsons, J Dods, KE Norman

**Affiliations:** Queen's University, School of Rehabilitation Therapy, Kingston, ON

**Purpose/Objectives & Rationale:** A pilot project was conducted in order to determine: a) The feasibility of delivering a faculty-moderated Case Workshop Series by multi-point video-conference using existing infrastructure (Ontario Telemedicine Network; OTN) during a Master's of Science in Physical Therapy (MScPT) clinical placement, b) The student-perceived value of a moderated Case Workshop held during a physical therapy clinical placement, and c) Differences in perceived value between first and second year MScPT students.

**Relevance to Physiotherapy Practice:** This project demonstrates a successful and innovative use of technology to support clinical education of MScPT students.

**Materials and Methods:** Participants: 26 first- and 34 second-year Queen's University MScPT students. Case Workshop Series: students met 4 times via video-conferencing (Groups of 5-10) in order to “workshop” patient cases with each other under the guidance of a faculty moderator. Program Evaluation: Case Workshop Questionnaires and Focus Group Interviews.

**Analysis:** Questionnaire responses were summarized by frequency counts. Transcribed data from the focus groups underwent a thematic analysis.

**Results:** 53% of the first year, and 100% of the second year participants reported that their participation enhanced their learning. Focus group interviews revealed the following themes: expectations, structure and content of sessions, the use of topic themes, moderator role, learning, resources available, social networking, technology, logistics and other challenges.

**Conclusions:** This project established that a faculty-moderated Case Workshop for small groups of MScPT students using existing video-conference infrastructure (i.e., OTN) was feasible within our context. Lessons from this project will assist a re-development of the model to the unique needs of first year students.

# Abstract: A002 – FACILITATION BY DISTANCE: A NOVEL METHOD FOR FACULTY DEVELOPMENT AND STUDENT LEARNING

**Published:** 2011-07

**Authors:** B Mori, E Yeung, R Davies

**Affiliations:** Department of Physical Therapy, University of Toronto

**Purpose/Objectives & Rationale:** The purpose of this study was to explore the use of web conferencing to facilitate small group learning from a distance.

**Relevance to Physiotherapy Practice:** Small group learning has traditionally relied on face-to-face facilitation and little is known about remote facilitation.

**Methods:** A qualitative approach using focus group methodology was used. Final year physiotherapy students enrolled in an orthopaedic course in 2008 or 2009 were invited to participate. Sixteen students participated. Three focus groups were conducted; two consisting of students who experienced remote facilitation and one consisting of students who experienced face-to-face facilitation. Participants were asked about their experiences with small group learning during this course, including group process and the role of their facilitator.

**Analysis:** The focus groups were audio taped and transcribed verbatim. Data were coded and analyzed for common ideas using a constant comparison approach.

**Results:** Regardless of method of facilitation, participants expressed a desire for similar facilitator traits and style of facilitation. Students in the remote facilitation groups identified unique challenges related to the facilitator and web conferencing technology.

**Conclusions:** Facilitation by distance is a feasible method for facilitating small group learning. To maximize the remote facilitation experience, attention should be paid to facilitator development that addresses the distinct features of facilitating a small group remotely, small group preparation, quality of technology, and room set up. Future faculty development initiatives should consider the unique role of the remote facilitator and its potential to engage those clinicians (as both teachers and learners) who would otherwise have limited ability to participate due to distance.

# Abstract: A004 – ACADEMIC DISHONESTY AMONG PHYSICAL THERAPY STUDENTS: A PILOT STUDY

**Published:** 2011-07

**Authors:** E Montuno, A Davidson, K Iwasaki, S Jones, J Martin, BE Gibson, B Mori

**Affiliations:** Department of Physical Therapy, University of Toronto

**Purpose/Objectives & Rationale:** Academic dishonesty (AD) refers to a number of behaviours that are associated with misconduct or misrepresentation to gain an academic advantage. The purpose of this study was to explore academically dishonest behaviours based on Physical Therapy (PT) students' current practices and educators' prior behaviours as PT students.

**Relevance to Physiotherapy Practice:** Exploration of both students' and educators' experiences is necessary to provide insight into the potential effect of changes in curriculum over time.

**Materials and Methods:** A web-based questionnaire was sent to 174 students and 250 educators from the PT program at the University of Toronto. The questionnaire gathered data on demographics, as well as the prevalence, seriousness and contributing factors regarding AD.

**Analysis:** Data were analysed with descriptive statistics and non-parametric tests.

**Results:** In all, 52.4% of educators and 44.3% of students responded to the questionnaire over a six-week data collection period. Scenarios rated the most serious were the least frequently performed by educators and students. The impact of generation on attitudes and prevalence of AD were not significant. The most commonly reported contributing factors of AD were school-related pressure, disagreement with evaluation methods and the perception that “everyone else does it.”

**Conclusions:** This study parallels the findings of similar research conducted on other healthcare programs. It suggests that AD occurs throughout the curriculum with greater incidence in situations associated with helping peers rather than personal gain. The consistency in behaviours across generations may reflect a ‘culture of cheating’ in the program that is accepted as the social norm and may be a function of the environment.

# Abstract: A006 – DÉVELOPPER LES COMPÉTENCES RELIÉES À LA PRATIQUE FACTUELLE CHEZ LES ÉTUDIANTS : UN DÉFI POUR LES PROFESSEURS

**Published:** 2011-07

**Authors:** D Cardinal

**Affiliations:** Université d'Ottawa, Faculté des sciences de la santé, Programme de physiothérapie

**Objectif :** Cette étude vise à identifier les moyens mis en œuvre par les professeurs pour développer les compétences en pratique factuelle chez les étudiants des programmes de maîtrise et du baccalauréat en physiothérapie et en ergothérapie de l'Université d'Ottawa et à évaluer la perception que les étudiants ont de leurs compétences en pratique factuelle.

**Pertinence pour la pratique de physiothérapie :** Bien que la littérature n'ait pas réussi à fournir des preuves convaincantes sur les bienfaits de l'enseignement de la pratique factuelle, la plupart des universités incorporent tout de même les éléments de la pratique factuelle dans leur curriculum. Très peu de recherches ont été effectuées dans le but de décrire les différentes approches préconisées par les professeurs pour l'enseignement des compétences liées à la pratique factuelle et de leurs effets sur les étudiants.

**Méthode :** Nous avons effectué un entretien auprès des professeurs enseignant la pratique factuelle (*n = *6) et administré un questionnaire à tous les finissants (*n =* 108).

**Analyse :** Nous avons analysé les données des entretiens avec le logiciel DRAP (Boudreault & Kalubi, 2006) et les données du questionnaire avec le logiciel SPSS.

**Résultats :** Nos résultats indiquent que la majorité des étudiants connaissent peu la terminologie en lien avec la pratique factuelle. Les étudiants de la maîtrise se perçoivent un peu mieux outillés que les étudiants du baccalauréat pour intégrer la pratique factuelle. Les professeurs semblent peu utiliser les stratégies d'enseignement recommandées par la littérature.

**Conclusions:** Nous retenons que les stratégies actuelles utilisées par les professeurs ne permettent que partiellement aux étudiants de développer les compétences visant à intégrer la pratique factuelle.

# Abstract: A018 – THE BOBATH CONCEPT IN PHYSIOTHERAPY CLINICAL PRACTICE

**Published:** 2011-07

**Purpose/Objectives & Rationale:** The purpose of this study was to explore how physiotherapists working in stroke care understand their role(s) in clinical practice and how their post-licensure experiences with the Bobath Concept are reflected in the ways that they practice.

**Relevance to Physiotherapy Practice:** The Bobath Concept, although not generally supported in the scientific literature, is identified by physiotherapists internationally as highly influencing their practice. There is a need to better understand how the Bobath Concept influences physiotherapy clinical practice given its popularity as a treatment approach in stroke care.

**Materials and Methods:** Registered physiotherapists with post-licensure education in the Bobath Concept as well as other neurological physiotherapy approaches and working in adult neurology for greater than two years participated in two activities. First, they responded in writing and verbally to a stroke clinical case. Second, each physiotherapist participated in an indepth interview regarding their pre and post-licensure educational experiences and their clinical practice.

**Analysis:** A hermeneutic phenomenology framework was adopted for this study, which included an adaptation of Carol Gilligan's “The Listening Guide” as a way to analyze the interview transcripts in a principled way."

**Results:** Common themes emerged from the clinical case and interview data analysis regarding the physiotherapists' understanding of the Bobath Concept and its influence on their clinical practice and perception of their role(s).

**Conclusions:** The Bobath Concept offers physiotherapists a unique perspective regarding their clinical practice. The results of this study support the need for further research into the Bobath Concept in physiotherapy clinical practice, and makes suggestions for future research study design and ongoing physiotherapy education.

# Abstract: A048 – A BEHAVIOURAL-BASED INTERVENTION INCREASES PRESCRIPTION OF INSPIRATORY MUSCLE TRAINING (IMT) FOR PEOPLE WITH CHRONIC OBSTRUCTIVE PULMONARY DISEASE (COPD)

**Published:** 2011-07

**Authors:** WD Reid, A Simms, L Li, A Hoens, EL Geddes, D Brooks

**Affiliations:** * Department of Physical Therapy, University of British Columbia, Vancouver; † School of Rehabilitation Science, McMaster University, Hamilton, ON; ‡ Department of Physical Therapy, University of Toronto, Toronto

**Purpose/Objectives & Rationale:** IMT has a strong evidence base for decreasing dyspnea and improving exercise tolerance and quality of life, however, it is not prescribed routinely in pulmonary rehabilitation. The purpose of this study is to assess the effectiveness of a behavioural (Beh)- versus an information (Inf)-based intervention for increasing health professionals' prescription of IMT to people with COPD during out-patient pulmonary rehabilitation.

**Relevance to Physiotherapy Practice:** Introduction of an evidenced based exercise such as IMT into daily practice likely requires addressing more issues besides providing evidence-based information.

**Materials and Methods:** Sixty-one health professionals and 488 COPD out-patients in six hospital-based pulmonary rehabilitation programs participated in the study. Centres were randomly allocated to Beh or Inf implementation interventions. Beh consisted of two interactive 45 min workshops: Session 1 provided hands-on instruction of inspiratory muscle testing and training and Session 2 focused on concerns related to IMT raised by the health professionals. Inf was a 90 min didactic lecture that focussed on evidence for IMT in COPD and prescription details for IMT. Both interventions were supplemented by handouts and an inspiratory muscle force meter. Beh group received a supply of Threshold© trainers.

**Analysis:** Chi^2^ and Mann-Whitney *U* tests were used to examine differences in health professionals' demographics and knowledge via a multiple choice exam, respectively.

**Results:** No COPD patients were prescribed IMT during the 6-month pre-intervention phase and this did not change after Inf. Prescription rates for IMT increased after Beh by 10.2% (95% CI, 5.7–17.1%).

**Conclusions:** Beh is more effective than Inf towards increasing health professionals' prescription of IMT for people with COPD.

# Abstract: A062 – ADMINISTRATION OF THE PATIENT CARE REFLECTION TOOL: A COMPARATIVE STUDY

**Published:** 2011-07

**Authors:** N Littke, A Lowe

**Affiliations:** * Alberta Health Services; † College of Physical Therapists of Alberta, Edmonton, AB

**Purpose/Objectives & Rationale:** This pilot study explored physiotherapists' experiences using the Patient Care Reflection Tool (PCRT) comparing two methods of administration: self-directed vs. facilitated interview as a tool assisting self reflection.

**Relevance to Physiotherapy Practice:** The PCRT was developed to assist physiotherapists engage in reflection on their clinical practice by providing a structured format of questions to guide their thoughts. The authors were interested in piloting the PCRT to determine whether the tool stimulated reflection and which of the two methods of administration was most valuable.

**Materials and Methods:** Five physiotherapists spanning a wide range of practice settings, specialities, years in practice and representing both male and female physiotherapists participated in this qualitative research study. Participants completed the PCRT individually and during a facilitated interview. They were then interviewed by the principal researcher using a semi-structured interview designed to gain understanding of their perspectives and experiences when using the tool.

**Analysis:** Two researchers independently utilized a thematic approach to analyse the transcripts from the primary, semi-structured interviews. The data was reduced into themes that represented the perspectives of the participants relative to the research question and project purpose as interpreted from within the perspective of the researchers.

**Results:** Three themes were identified by the participants: (1) participants preferred self-directed reflection; (2) a structured process was valuable; and (3) acknowledgement of the limitations inherent in self-reflection and the need for behavioural drivers to ensure professional accountability for maintaining competent practice.

**Conclusions:** The PRCT is an easily administered tool that can provide physiotherapists with a structured means to stimulate reflection on clinical practice.

# Abstract: A066 – EXPERIENCES WITH USING REFLECTION AS A PEDAGOGICAL TOOL IN PAIN EDUCATION FOR PHYSIOTHERAPY STUDENTS

**Published:** 2011-07

**Authors:** DM Walton

**Affiliations:** The University of Western Ontario, School of Physical Therapy, London, ON

**Purpose/Objectives & Rationale:** To describe the usefulness of reflective diaries as a teaching and evaluative tool in pain education for physiotherapy students. Both successes and challenges will be addressed.

**Relevance to Physiotherapy Practice:** Pain is the most common symptom treated by physiotherapists. Pain education presents a unique challenge in that pain science is constantly changing. The topic does not lend itself well to traditional didactic lecture and quantitative assessment of knowledge. Reflective diaries represent a novel approach to these challenges.

**Materials and Methods:** Data are taken from a senior-year elective course in understanding pain for physical therapists. Students are required to complete a weekly reflective diary based on the previous week's discussion, and to submit the diary as the main evaluative component at the end of the course.

**Analysis:** Qualitative interpretation of select passages from the reflective diaries of 60 students will be presented using Bloom's taxonomy of learning as a framework. Mean student ratings of the course as a learning experience and the fairness of the evaluation procedure were used to evaluate the student's perspective.

**Results:** After 3 years (60 students), the mean student rating for the course as a learning experience is 6.9/7 with the mean rating of fairness of reflective diaries as an evaluation procedure at 6.1/7. The diaries are useful for demonstrating learning across levels of Bloom's taxonomy which would not have been captured using traditional evaluation methods.

**Conclusions:** Reflective diaries are a novel and promising approach to achieving and evaluating deeper learning of pain concepts in a classroom setting.

# Abstract: A067 – INTRAPROFESSIONAL CLASSROOM BASED ACTIVITIES

**Published:** 2011-07

**Authors:** W Jelley, N Larocque

**Affiliations:** * University of Ottawa; † La Cité collégiale, Ottawa

**Purpose/Objectives & Rationale:** To develop a classroom-based collaborative activity involving physical therapy and physiotherapist assistant students.

**Relevance to Physiotherapy Practice:** Physiotherapy and physiotherapist assistant students frequently get little or no time to interact and work together prior to graduation and entering the work force. Despite this there is an expectation that they will be able to work as a collaborative intraprofessional team.

**Materials and Methods:** A half-day classroom-based intraprofessioanl activity was developed by two educators from the University of Ottawa and La Cité collégiale. The students were divided into mixed teams. The classroom activity included introductions, ice-breaker activities and a case competition. The cases were de-idenitified cases that had been solicited from physiotherapists practicing as part of an intraprofessional team in Ottawa. At the conclusion of the activity the students were asked to complete a questionnaire. No idenitifying information was collected and students were asked to include constructive feedback about the event.

**Analysis:** The survey responses were analyzed and the responses coded in NVivo 8.

**Results:** The activity was viewed by the students as a successful means to learn about the scope of practice, roles and responsibilities of their colleagues on an intraprofessional team. The case permited the physiotherapy and physiotherapist assistant students to apply their knowledege and explain their actions and plans.

**Conclusions:** Opportunities, such as a classroom-based activity with a case competition for intraprofessional student teams can encourage the sharing of information about education, scope of practice, professional and ethical perspectives. The students requested that the activity become an annual event.

# Abstract: A068 – POLICIES, BARRIERS, AND INCENTIVES IMPACTING PT STUDENT CLINICAL PLACEMENTS IN THE COMMUNITY/HOME CARE SETTING

**Published:** 2011-07

**Authors:** HEA MacPhail, T Alappat, B Mullen, L Napoli

**Affiliations:** School of Physical Therapy, University of Western Ontario, London, ON

**Purpose/Objectives & Rationale:** To investigate policies, barriers, and incentives surrounding community/home care clinical placements for physical therapy (PT) students.

**Relevance to Physiotherapy Practice:** The community/home care environment is an expanding area of health care provision. However, there are limited clinical placement opportunities for PT students in this, mainly private, health care environment. No research exists to guide future decision-making.

**Materials and Methods:** An online survey (multiple choice, Likert scale, short answer questions) was used to collect quantitative / qualitative data from Community Care Access Centre (CCAC) administrators, private home care company managers, and home care physical therapists in three Local Health Integrated Networks in ON.

**Analysis:** Percentages were calculated for quantitative survey data. Qualitative data was analysed by investigators highlighting common words and themes.

**Results:** Twenty-two surveys were completed. PT participation was poor and correlated directly with financial reimbursement provided by their company to complete the survey. Seventy two percent of private home care company managers and PTs felt that in a fee-for-service environment, a PT's income decreases while supervising a student during placement. Seventy-six percent of respondents indicated their company had guidelines related to student placements and supervision but no participant was able to provide any written documentation regarding these guidelines.

**Conclusions:** Clarity of CCAC and company policy related to student supervision in the community/home care setting is essential. An alternate model of payment for PTs when supervising students in the community / home care setting is required. The majority of Canadian PT students presently graduate with minimum or no exposure to a community / home care environment.

# Abstract: A007 – PROPERTIES OF THE SLANSS TOOL FOR ASSESSMENT AND PROGNOSIS IN WHIPLASH

**Published:** 2011-07

**Authors:** DM Walton, A Smith

**Affiliations:** * The University of Western Ontario, School of Physical Therapy, London ON

**Purpose/Objectives & Rationale:** Whiplash-associated disorder (WAD) is a common condition representing considerable burden. The purpose of this study was to evaluate the validity of the Self-report version of the Leeds Assessment for Neuropathic Signs and Symptoms (SLANSS) for use in evaluating people with WAD.

**Relevance to Physiotherapy Practice:** Physiotherapists often provide rehabilitation services for people with WAD. Assessment of the condition is challenging however. Previous reports indicate the SLANSS tool may be useful for this population. However it has never been formally validated for use in WAD.

**Materials and Methods:** Data from two separate databases were combined. All subjects presented for physiotherapy treatment of WAD following motor vehicle accident. The SLANSS was part of a battery of tests performed on initial assessment. Pain threshold (PPT), neck disability index (NDI), pain intensity (NRS), age, sex and duration of symptoms were also extracted from the databases. A subset of patients was followed up 3 months later. Analysis: Exploratory factor analysis was performed to evaluate the factor structure of the the SLANSS. Concurrent validity was evaluated through bivariate correlations with NDI, NRS and PPT. Moderators (age, sex and duration) were explored through tests of association. Linear regression was used to evaluate the predictive validity of the SLANSS on 3-month outcomes.

**Results:** Factor analysis revealed three factors. Hypotheses for concurrent validity were satisfied. No moderators were identified. SLANSS explained 16% of variance in 3-month outcomes.

**Conclusions:** The validity of the SLANSS has been supported for use in WAD.

# Abstract: A008 – THE USEFULNESS OF PRESSURE PAIN THRESHOLD AS AN ASSESSMENT TOOL FOR PEOPLE WITH WHIPLASH

**Published:** 2011-07

**Authors:** DM Walton, L Levesque

**Affiliations:** * The University of Western Ontario, School of Physical Therapy, London ON

**Purpose/Objectives & Rationale:** Previous authors have shown that pressure pain threshold (PPT) holds promise as a prognostic tool in acute whiplash. Until now, these studies have been performed using lab-based instruments that are not accessible to clinicians. This presentation will describe the clinimetric properties of a more accessible, lower cost digital algometer in terms of reliability, population norms, and predictive validity.

**Relevance to Physiotherapy Practice:** Whiplash-associated disorder (WAD) is a common outcome of motor vehicle accident. Judicious assessment requires patient self-report and clinical observation. PPT stands to add potentially useful information to clinical assessment.

**Materials and Methods:** Healthy volunteers and people with neck pain were tested by two raters and on two separate days to determine intra-, inter- and test-retest reliability. Data from three databases were combined to determine normative values and identify important moderators. Finally, people with acute WAD were evaluated within 30 days of injury and again 3 months later to determine short-term outcomes.

**Analysis:** Intra-class correlation coefficients for reliability, means and standard deviations for norms, simple bivariate correlations for moderators, and multiple regression for prognosis.

**Results:** Reliability coefficients ranged from 0.76 to 0.97. Means and standard deviations were in keeping with previously reported lab-based values. Sex, age and pain intensity were important moderators. Regression showed that PPT accounted for a significant 14.2% of variance in short-term disability, after controlling for sex, age and pain intensity.

**Conclusions:** PPT can be reliably tested in a clinical setting using an accessible digital algometer. PPT holds promise for assessment of prognosis in acute WAD.

# Abstract: A009 – RESPONDERS AND NON-RESPONDERS OF CERVICAL FACET BLOCKS FOR CHRONIC WHIPLASH SHOW SIMILAR PHYSICAL AND PSYCHOLOGICAL FEATURES

**Published:** 2011-07

**Authors:** A Smith, M Sterling, G Jull, G Schneider, B Frizzell, A Hooper

**Affiliations:** * University of Queensland, Australia; † University of Calgary

**Purpose/Objectives & Rationale:** To compare baseline physical and psychological characteristics of responder and non-responder individuals with chronic WAD, who undergo cervical facet double block (FB) procedures.

**Relevance to Physiotherapy Practice:** Diagnostic FB injections are recommended for individuals with chronic WAD who do not respond to conservative physiotherapy. It is important that physiotherapists recognize those patients who may benefit from FB.

**Materials and Methods:** This cross-sectional study involved 30 WAD individuals who responded to cervical FB procedures (WAD_RF); 19 WAD individuals who did not respond (WAD_C) and 21 Healthy Control (HC) individuals. Quantitative Sensory Testing (pressure; thermal pain thresholds, brachial plexus provocation test), nociceptor flexor reflex (NFR) and sLANSS questionnaire were measured. Motor function (cervical range of movement (ROM); activity of the superficial neck flexors (EMG) and activity levels (IPAQ) were also measured.

Psychological distress (General Health Questionnaire28), post-traumatic stress disorder symptoms (PDS), pain catastrophization (PCS) and general health and well being(SF-36) data were also collected.

**Analysis:** Box plots (QST, sensori-motor and questionnaires) were generated. Kruskal-Wallis rank tests were used to determine if there was a significant difference between the WAD_RF and control group (WAD-C and HC) baseline measures.

**Results:** Both WAD groups demonstrated local and widespread hyperalgesia to pressure (*p <* 0.005) and thermal stimuli (*p <* 0.05). Both WAD groups demonstrated decreased neck ROM in all directions (*p <* 0.0001).WAD_C individuals demonstrated increased EMG levels throughout the CCFT(*p <* 0.05). Both WAD groups had lower activity levels (IPAQ) than HC (*p <* 0.05). Both WAD groups demonstrated higher levels of psychological distress (GHQ-28, SF-36, *p < *0.0001), post-traumatic stress symptoms, pain catastrophization and elevated sLANSS scores (*p <* 0.05).

**Conclusions:** All individuals in this study experiencing whiplash injury demonstrated a complex presentation of motor dysfunction, widespread sensory disturbance and psychological distress, with responders to FB procedures not readily identifiable via these measures.

# Abstract: A010 – UPPER TRAPEZIUS RECRUITMENT PATTERNS WITH A REPETITIVE UPPER LIMB TASK IN FEMALES WITH WHIPLASH ASSOCIATED DISORDER II AND HEALTHY WOMEN

**Published:** 2011-07

**Authors:** ME Belot, G Jull

**Affiliations:** * West 4th Physiotherapy Clinic, Vancouver, BC; † University of Queensland, School of Health and Rehabilitation Sciences, St. Lucia, Australia

**Purpose/Objectives & Rationale:** The objectives were to determine: a. differences in upper trapezius recruitment patterns between women with persistent neck pain and whiplash associated disorders (WAD) grade II, and healthy women; and b. differences between subgroups of women with WADII and healthy women.

**Relevance to Physiotherapy Practice:** This research could aid physical therapists in reducing and/or preventing chronic pain and disability.

**Materials and Methods:** Surface electromyography (EMG) was recorded from two sites over each upper trapezius before, during, and after a seated, repetitive unilateral upper extremity task. Women with WADII then underwent a physical examination by a physiotherapist in order to confirm their WADII classification as well as assign a Sterling classification.

**Analysis:** A three-way mixed ANOVA (group by trial by time in trial) was performed for each muscle site for median frequency and mean EMG amplitude. This was repeated, grouping by WADII vs. healthy, subgrouping by disability level and subgrouping using a modified Sterling Classification.

**Results:** Women with WADII demonstrated lower levels of activation of upper trapezius of the dominant limb during the repetitive task relative to the healthy group. In addition, signs of fatigue were evident in the posterior fibres of upper trapezius of the dominant limb, particularly, in the group with the highest disability level (NDI Score ≥30).

**Conclusions:** Upper trapezius motor impairments were associated with persistent pain in women with WADII but were not reliably predicted by level of disability or Sterling classification. Surface electromyography of upper trapezius in WADII may be a useful clinical tool, however, further research is indicated to guide clinical practice.

# Abstract: A012 – A SNAPSHOT OF IN-PATIENT ONCOLOGY REHABILITATION IN TORONTO

**Published:** 2011-07

**Authors:** SE McEwen, S Elmi, G Liu, M Bishev

**Affiliations:** St. John's Rehab Hospital, Toronto

**Purpose/Objectives & Rationale:** 1. Develop a profile of patients discharged from a specialized, inpatient oncology rehabilitation unit; and 2. Explore the relationships between patient characteristics and clinically important functional improvements.

**Relevance to Physiotherapy Practice:** The role of cancer rehabilitation is transitioning from palliative care to more active complex rehabilitation designed to remediate functional loss. However, there have been limited studies exploring inpatient oncology rehabilitation. Exploratory research is required to help physiotherapists build the oncology rehabilitation evidence base.

**Materials and Methods:** We conducted a retrospective review of health records of all patients discharged from an inpatient oncology rehabilitation unit between November 2008 and March 2010. Data elements included sex, age, diagnosis, living arrangements, living setting, informal support received, discharge destination, comorbidities, Functional Independence Measure (FIM™) scores, and length of stay (LOS).

**Analysis:** Descriptive statistics were compiled for all variables. Logistic regression was conducted to determine the variables associated with FIM™ improvements meeting a minimal clinically important difference (MCID) of at least 17 points.

**Results:** Data were obtained from 144 patients. Age ranged from 38 to 97 years (mean = 74.1, SD = 12.2), 60.4% were women, and 42% lived alone. The average LOS was 20.8 days (SD=11.0). The most common diagnoses were colorectal cancer (*n =* 44, 30.6%), metastatic disease (*n =* 17, 11.8%), and bladder cancer (*n =* 11, 7.6%). Being male, less than 65 years of age, and living with another were all associated with MCID FIM™ scores, although only living with another was an independent predictor.

**Conclusions:** These findings provide a foundation for comparison with other inpatient oncology rehabilitation programs and for future research to build the oncology rehabilitation evidence base.

# Abstract: A014 – NEW CONCEPTS IN THE PATHOGENESIS AND REHABILITATION OF ROTATOR CUFF TEARS: AN IN VIVO ULTRASOUND STUDY

**Published:** 2011-07

**Authors:** S Kim, E Boynton, R Bleakney, T Rindlisbacher

**Affiliations:** * School of Physical Therapy, University of Saskatchewan; † Graduate Department of Rehabilitation Science, University of Toronto; ‡ Musculoskeletal Division, Joint Department of Medical Imaging, University Health Network, Mount Sinai Hospital and Women's College Hospital, University of Toronto; § Cleveland Clinic Canada and Sunnybrook Health Sciences Centre, University of Toronto

**Purpose/Objectives & Rationale:** The musculotendinous architecture of supraspinatus is complex with architecturally distinct regions and parts. To date, however, the architecture of the distinct regions and its relation to joint position has not been investigated. Thus, the purpose of the study was to quantify the dynamic architecture of the distinct regions of supraspinatus using ultrasound.

**Relevance to Physiotherapy Practice:** Rehabilitation is dependent on an accurate understanding of muscle function. Since muscle architecture is a primary determinant of muscle function, investigating the detailed architecture of supraspinatus is essential to optimal management.

**Materials and Methods:** Seventeen subjects (8M, 9F) mean age 36.4±12.7 years without tendon pathology were recruited. Supraspinatus was scanned in relaxed and contracted states. For the contracted state, supraspinatus was scanned with the shoulder actively abducted to 60° with neutral glenohumeral rotation; 80° external rotation; 80° internal rotation.

**Analysis:** Fiber bundle lengths (FBL) and pennation angles (PA) of distinct regions and muscle thickness were computed. Parameters between regions and changes between relaxed and contracted states were analyzed using paired *t-*tests and repeated measures ANOVA (*p <* 0.05).

**Results:** On contraction in the anterior region, mean percentage of FBL shortening ranged between 9% and 21%. In the posterior region, shortening of approximately 2% only occurred in two of the three positions. For the anterior region, mean PA increased the least in the externally rotated position and mean PA of the middle part was smaller than the deep part for all states.

**Conclusions:** Findings suggest changes in the architecture are not uniform throughout the supraspinatus and joint position may play an important role in force production.

# Abstract: A015 – ULTRASOUND INVESTIGATION OF THE MUSCULOTENDINOUS ARCHITECTURE OF THE PATHOLOGICAL SUPRASPINATUS: AN IN VIVO PILOT STUDY OF FULL-THICKNESS TENDON TEARS

**Published:** 2011-07

**Authors:** S Kim, B Rosser, R Bleakney, T Rindlisbacher, E Boynton

**Affiliations:** * School of Physical Therapy, University of Saskatchewan; † Department of Anatomy, University of Saskatchewan; ‡ Musculoskeletal Division, Joint Department of Medical Imaging, University Health Network, Mount Sinai Hospital and Women's College Hospital, University of Toronto; § Cleveland Clinic Canada and Sunnybrook Health Sciences Centre, University of Toronto; ¶ Graduate Department of Rehabilitation Science, University of Toronto

**Purpose/Objectives & Rationale:** Supraspinatus tendon tears are prevelant and can lead to architectural changes of the muscle. The purpose of the study was to quantify the fiber bundle architecture within the distinct regions of supraspinatus in subjects with a full-thickness tendon tear using an ultrasound protocol previously developed in our laboratory, and to compare findings with age and gender matched normal controls.

**Relevance to Physiotherapy Practice:** Novel rehabilitative approaches that may prevent and/or reverse pathological changes in musculotendinous architecture are dependent on an accurate understanding of the morphological changes that occur with tendon tears.

**Materials and Methods:** Twelve supraspinatus muscles were scanned (12 MHz transducer) from eight subjects (6M/2F) with full-thickness tendon tears (mean age 57±6.0 y). For the contracted state, supraspinatus was scanned with shoulder in neutral rotation and 60° of active abduction.

**Analysis:** Fiber bundle length (FBL) and pennation angle (PA) were computed for each distinct region. Wilcoxon Signed Rank and Mann-Whitney Tests were carried out to compare means between relaxed and contracted states and for comparison between the pathologic subjects and normal controls respectively.

**Results:** For the anterior region, mean FBL of the pathologic supraspinatus was similar with normal controls. Mean PA, however, was smaller in the pathologic muscle compared to normal controls; the difference was significant in the contracted state (*p <* 0.05). Mean FBL in the posterior region in both relaxed and contracted states was significantly shorter in the pathologic muscle compared to normal controls (*p <* 0.05).

**Conclusions:** Large differences in changes of parameters in subjects with tendon tears and controls suggest that evaluation of muscle changes is important when planning treatment.

# Abstract: A016 – STANDARD ERROR OF MEASUREMENT AND MINIMAL CLINICAL DIFFERENCE ASSOCIATED WITH OVERHEAD SHOULDER ROTATION AND HORIZONTAL ADDUCTION

**Published:** 2011-07

**Authors:** JC Chepeha, DJM Magee, L Beaupre

**Affiliations:** Department of Physical Therapy, Faculty of Rehabilitation Medicine, University of Alberta, Edmonton, AB

**Purpose/Objectives & Rationale:** To determine the standard error of measurement (SEM) and minimal clinical difference (MCD) associated with measuring internal rotation, external rotation and horizontal adduction at the glenohumeral joint.

**Relevance to Physiotherapy Practice:** Goniometry is the most commonly used method for measuring range of motion (ROM). However, few studies have determined the reliability of goniometry, particularly in regards to overhead shoulder rotation; an important functional position for overhead athletes and workers.

**Materials and Methods:** Using a cross-sectional design, 30 men and women (22–51 years old) with 47 shoulders underwent standard goniometric assessment of internal and external rotation in 90° of abduction and horizontal-adduction by 2 examiners blinded to measurement.

**Analysis:** Intra- and inter-rater intraclass correlation coefficient (ICC) values were calculated using a 2-way random effects analysis of variance (ANOVA). SEM was calculated to quantify magnitude of error. MCD, estimated from the SEM, was also calculated to represent the smallest amount of ‘true meaningful or important’ change rather than measurement error.

**Results:** ICC values revealed excellent reliability both within and between therapists' shoulder rotation ROM measures (0.915–0.966) and good reliability for horizontal adduction measures (0.761–0.834). SEM ranged from 2.3°–4.1° for ICCintra and 2.2°–3.6° for ICCinter. MCDintra values for rotation ranged from 10°–11.3° and 6.4°–7° for horizontal adduction. MCDinter values were comparable at 8.5° and 10° for internal and external rotation and 6.2° for horizontal adduction.

**Conclusions:** Shoulder internal and external rotation ROM can be reliably measured in 90° abduction; an important, functional position for overhead athletes and workers. The MCD values for all three movements indicate that small changes in measurement represent meaningful clinical change.

# Abstract: A019 – EVALUATION OF THE SPECIALIZED COMMUNITY STROKE REHABILITATION TEAMS: MAKING POSSIBILITIES COME TO LIFE

**Published:** 2011-07

**Authors:** DA Willems, G Tomaszewski, P Gilmore, D Noble, M Crites

**Affiliations:** * Southwestern Ontario Stroke Network, London ON; † Parkwood Hospital, St Josephs Health Care, London, ON

**Purpose/Objectives & Rationale:** Report the outcomes of a new interprofessional team model of care delivery for stroke rehabilitation in the community.

**Relevance to Physiotherapy Practice:** According to the Canadian Stroke Strategy Best Practice Recommendations for Stroke Care, “After leaving hospital, stroke survivors must have access to specialized stroke care and rehabilitation services appropriate to their needs.” Yet, multiple stakeholders identified access to rehabilitation services for stroke in the community as a gap. A new model of care, Specialized Community Stroke Rehabilitation Teams (CSRT), was created to address this need in one health region.

**Materials and Methods:** Functional Independence Measure (FIM), Stroke Impact Scale (SIS), Hospital Anxiety and Depression Scale and Caregiver Assistance Scale data were collected on admission, discharge and 6-month follow-up. Qualitative data was also collected.

**Analysis:** Changes in outcome measures were analyzed using paired *t*-tests.

**Results:** Admission and discharge data was available for 130 clients; mean age 68 years. Follow-up data was available for 30 of those clients. CSRT clients made statistically significant gains on the FIM (*p <* 0.001), and the physical (*p =* 0.01), psychosocial (*p <* 0.001) and recovery (*p <* 0.001) domains of the SIS between admission and discharge, that were maintained at follow-up. Clients also displayed fewer symptoms of depression (*p <* 0.001) and required less caregiver assistance (*p =* 0.01) at discharge compared to admission. A high percentage (> 88%), were satisfied with team care reporting improved quality of life and ability to stay at home.

**Conclusions:** Both quantitative and qualitative results confirm the effectiveness of this new model of a specialized interprofessional team providing rehabilitation and community reintegration, for improving function and quality of life for stroke survivors.

# Abstract: A020 – EFFECT OF REHABILITATION STRATEGIES ON AFFECTED LIMB WEIGHT-BEARING IN PEOPLE WITH STROKE DURING SIT-TO-STAND: A PILOT STUDY

**Published:** 2011-07

**Authors:** CK Gray, KY Lui, E Culham

**Affiliations:** School of Rehabilitation Therapy, Queen's University, Kingston, ON

**Purpose/Objectives & Rationale:** To investigate strategies designed to alter affected limb weight-bearing in people with stroke when rising to stand from sitting.

**Relevance to Physiotherapy Practice:** Inability to weight bear effectively through the affected limb during sit-to-stand leads to balance impairment and increased risk for falls. (Cheng et al., 2001). The effect of strategies designed to increase affected limb weight-bearing during sit-to-stand remains unclear.

**Materials and Methods:** Five people with stroke participated in this study (57 + 8.9 y). Forces under each foot and the chair were collected using three force plates. Subjects were instrumented with clusters of infrared light emitting diodes for motion analysis. Strategies included placement of solid (2.54, 5.08, 7.62 and 10.16 centimeters) or foam (2.72 and 6.63 kg density) blocks under the unaffected limb, and placing the unaffected limb ahead (one quarter and one half foot length) of the affected limb. Baseline measures were also obtained. Three trials were collected for each condition for a total of 27 trials.

**Analysis:** Repeated measures ANOVA.

**Results:** The 10.16-cm block, 2.72-kg foam and the two foot positions significantly increased affected limb weight-bearing at seat-off compared to baseline (*p <* 0.05). Only the four inch block significantly increased the average amount of affected limb weight-bearing from seat-off until the end of the task compared to baseline (*p <* 0.05).

**Conclusions:** Preliminary results suggest that the most effective strategy for increasing affected limb weight-bearing is placement of the unaffected foot on a 10.16-cm block. The 2.72-kg foam and the two altered foot positions may also be effective strategies for increasing affected limb weight-bearing.

# Abstract: A021 – PHYSICAL ACTIVITY AND EXERCISE INTENSITY POST STROKE

**Published:** 2011-07

**Authors:** EK Stokes, E O'Grady, D O'Gorman

**Affiliations:** * Department of Physiotherapy, Trinity College Dublin; † School of Health and Human Performance, Dublin City University, Ireland

**Purpose/Objectives & Rationale:** To measure and compare habitual physical activity levels in people post stroke with age and gender matched controls, and to quantify the exercise intensity at various step rates for individuals post stroke.

**Relevance to Physiotherapy Practice:** Stroke has been shown to be a major cause of death and disability in every society in which it has been studied. Physical activity is a modifiable risk factor for stroke and is key to both prevention and treatment of stroke. Understanding step rate and intensity will assist in exercise prescription.

**Materials and Methods:** Samples of convenience of 34 ambulant, community-dwelling, individuals post stroke and 25 healthy age and gender matched controls were recruited. A subset of 10 individuals participated in the study of exercise intensity. Participants wore a StepWatch activity monitor for 7 days. Intensity of exercise was measured in terms of VO2peak using an Oxycon Mobile system.

**Analysis:** Activity data was calculated from mean values over 7 days. Analysis included 2-sample *t-*tests and multiple regression.

**Results:** A statistically significant difference (*t* = 9.52, *p <* 0.001) in mean daily steps was evident between individuals post stroke and controls. Measured VO2peak among individuals post stroke ranged from 11.1-22.8 mL/kg/min (mean 15.91 mL/kg/min). All individuals were exercising at moderate intensity at a step rate of 30 steps/leg/min.

**Conclusions:** Individuals post stroke were significantly less active than their healthy peers. Due to cardiovascular deconditioning and increased energy cost of hemiparetic gait, individuals post stroke achieved moderate intensity exercise at relatively low step rates, which may be sufficient to induce cardiovascular training.

# Abstract: A022 – DOES THE MCGILL PAIN SUBSCALE DIFFERENTIATE BETWEEN NEUROPATHIC AND NON-NEUROPATHIC CHRONIC PAIN IN THE TOTAL JOINT ARTHROPLASTY POPULATION?

**Published:** 2011-07

**Authors:** D Boljanovic-Susic, M Rachevitz, J Gollish, I MacRitchie, J de Beer, P Stratford, LJ Woodhouse

**Affiliations:** * Holland Orthopaedic & Arthritic Centre, Toronto; † Department of Surgery, Univeristy of Toronto; ‡ Sunnybrook HS, Toronto; § Orthopaedic Surgery Hamilton Health Sciences; ¶ School of Rehabilitation Science, Institute of Applied Health Sciences, McMaster University, Hamilton, ON

**Purpose/Objectives & Rationale:** Recent European studies (Nikolajsen et al., 2006, 2009) indicate that 28% of total joint arthroplasty (TJA) recipients experience chronic pain following surgery. The purpose of this study was to examine the ability of the McGill Pain subscale to identify individuals with neuropathic pain (NP) following TJA.

**Relevance to Physiotherapy Practice:** Escalating arthroplasty volumes will increase the prevalence of those who experience post-surgical NP symptoms. Few studies have evaluated chronic neuropathic pain in patients following total hip (THA) or knee (TKA) joint arthroplasty.

**Materials and Methods:** A cross sectional survey included 41 individuals within 2 years post primary, unilateral THA or TKA. Participants reported their post surgical pain to be the same or worse than pre operatively. All participants completed SLANSS and MPQ-2 SF questionnaires.

**Analysis:** Receiver Operating Characteristic (ROC) analysis was performed to evaluate the ability of the NP subscale of the MPQ-2 SF to correctly identify patients with neuropathic pain following TJA. Kappa analyses were used to measure the agreement between the MPQ-2-SF and S-LANSS scores.

**Results:** Cohen's Kappa between the two measures was 0.53 (95% CL: 0.27, 0.80). The ROC analysis yielded an area under the curve of 0.88 (95% CI, 0.78-0.99). The MPQ-2-SF NP subscale cut off score that maximized sensitivity [0.82 (95% CI, 0.48-0.97)] and specificity [0.83 (95% CI, 0.65-0.94)] was 1.08 points.

**Conclusions:** The MPQ-2 SF neuropathic subscale is a moderately adequate measure for discriminating between neuropathic and non-neuropathic chronic pain following TJA.

# Abstract: A023 – EFFECTS OF PREHABILITATION ON PAIN AND PHYSICAL FUNCTION OF PATIENTS WITH SEVERE DISABILITY AWAITING TJA: RESULTS OF A PILOT STUDY

**Published:** 2011-07

**Authors:** F Desmeules, J Hall, M Bellavia, G Manson, J Gillem, M Hannah, LJ Woodhouse

**Affiliations:** * School of Rehabilitation, University of Montreal, Montreal; † Hamilton Health Sciences Corporation; ‡ School of Rehabilitation Science, McMaster University, Hamilton, ON; § Holland Orthopaedic & Arthritic Centre of Sunnybrook Health Sciences, Toronto

**Purpose/Objectives & Rationale:** Purpose/Rationale: The demand for total hip and knee joint arthroplasty (TJA) is escalating. Establishing prehabilitation programs may ensure fitness for TJA surgery. The purpose of this study was to evaluate the effects of prehabilitation on pain and physical function of patients with severe disability who are awaiting TJA.

**Relevance to Physiotherapy Practice:** Previous research suggests that prehabilitation may shorten hospital stay and improve clinical outcomes (pain, function and quality of life) of individuals undergoing TJA.

**Materials and Methods:** A subset (*n =* 28) of individuals with severe disability was identified from a group of 650 patients with hip or knee osteoarthritis awaiting TJA and attended a clinic for a prehabilitation (exercise and education) program. All participants completed self-report (Lower Extremity Functional Scale:LEFS, Visual analog scale for pain:VAS), and performance (40 m self-paced walk:SPW, stair, and timed-up-and-go:TUG) measures.

**Analysis:** Participant characteristics were examined using univariate analyses. Paired Student's *t-*tests and Wilcoxon signed rank tests were used to compare pre vs. post prehabilitation pain and function.

**Results:** The 28 individuals (16 females) with mean age of 67±10 years and BMI 33±8 awaiting TJA (10 hips/18 knees) participated in the prehabilitation program of 9±6 weeks duration. A significant improvement was seen in LEFS score [7.6; 95% CI, 1.7–13.5] after prehabilitation and in terms of performance measures, a significant improvement was seen on the SPW (-11 s.±21.5; *p < *0.001) and on the TUG (-4.2s.±5.6; *p =* 0.005) tests.

**Conclusion:** Prehabilitation improves physical function even in the most severely compromised patients awaiting TJA.

# Abstract: A024 – DIFFERENCES IN TIBIAL ROTATION DURING WALKING IN PATIENTS WITH ANTERIOR CRUCIATE LIGAMENT DEFICIENCY AND KNEE OSTEOARTHRITIS

**Published:** 2011-07

**Authors:** RF Moyer, TB Birmingham, SM Robbins, IC Jones, TR Jenkyn, JR Giffin

**Affiliations:** * Wolf Orthopaedic Biomechanics Laboratory, Fowler Kennedy Sport Medicine Clinic, The University of Western Ontario, London, ON; † School of Physical Therapy, The University of Western Ontario, London, ON; ‡ School of Physical Therapy, Dalhousie, Halifax; § Department of Mechanical and Materials Engineering, The University of Western Ontario, London, ON; ¶ Department of Surgery, The University of Western Ontario, London, ON

**Purpose/Objectives & Rationale:** To compare tibial rotation during walking in both limbs of patients with unilateral anterior cruciate ligament deficiency and mild or severe knee osteoarthritis.

**Relevance to Physiotherapy Practice:** Greater tibial rotation during walking in the anterior cruciate ligament reconstructed knee compared to the healthy contra-lateral knee is suggested to predispose patients to knee osteoarthritis. It is presently unclear whether this abnormal knee motion results from injury or surgery, and we are unaware of previous research directly testing whether excess tibial rotation exists in patients who have developed knee osteoarthritis after an anterior cruciate ligament tear.

**Materials and Methods:** 68 patients with unilateral anterior cruciate ligament deficiency and knee osteoarthritis underwent 3-dimensional gait analysis. We assessed transverse plane kinematics in both limbs at 4 points in stance.

**Analysis:** We used a 2-factor repeated measures ANOVA to compare limbs at heel-strike, mid-stance, terminal extension and toe-off. Based on that analysis, we then compared differences between limbs (external rotation offset) in patients with mild and severe osteoarthritis using independent *t-*tests.

**Results:** There was a limb by point-in-stance interaction (*p =* 0.003). External rotation was greater in the affected limb at heel-strike (*p <* 0.001) and mid-stance (*p <* 0.001). External rotation offset at heel-strike was greater (*p =* 0.013) in patients with severe osteoarthritis.

**Conclusions:** A between-limb tibial external rotation offset exists at heel-strike and mid-stance in patients with anterior cruciate ligament deficiency and knee osteoarthritis, and is greater in patients with more severe degeneration. Findings are consistent with the hypothesis that abnormal transverse plane kinematics are involved in the progression of knee osteoarthritis after an anterior cruciate ligament tear.

# Abstract: A026 – MEASURING WALKING CAPACITY AFTER TOTAL JOINT ARTHROPLASTY: 2- VERSUS 6-MINUTE WALK TESTS

**Published:** 2011-07

**Authors:** M Lung, B Mahy, L Edmison, SL Yasin, M Gomez, D Brooks, N Salbach

**Affiliations:** * St. John's Rehab Hospital, Physiotherapy, Toronto; † Department of Biology, McMaster University, Hamilton, ON; ‡ Department of Clinical Research, St. John's Rehab Hospital, Toronto; § Department of Physical Therapy, University of Toronto, Toronto

**Purpose/Objectives & Rationale:** The purpose is to examine the construct validity of the two-minute (2MWT) and six-minute (6MWT) walk tests among in-patients receiving rehabilitation following total joint arthroplasty (TJA) by evaluating the correlations of 2MWT and 6MWT distances with ratings of health-related quality of life, pain, stiffness, and physical function.

**Relevance to Physiotherapy Practice:** In a fast-paced in-patient unit, time and space may be limited. Furthermore, not all patients are able to tolerate the 6MWT; therefore, the 2MWT may offer a practical alternative.

**Materials and Methods:** Fifty participants underwent both the 2MWT and 6MWT. During the one-hour rest period between the walk tests, participants completed two validated health scales: the EuroQoL-Visual Analogue (EQ-VAS) measure of quality of life, and the Western Ontario and McMaster University (WOMAC) Osteoarthritis Index.

**Analysis:** We correlated walking distance scores with EQ-VAS and WOMAC sub- and total scores using Spearman's Correlation.

**Results:** Both the 2MWT (mean: 86.7 ± 21.0 m) and 6MWT (mean: 250.1 ± 59.7 m) scores were significantly correlated with EQ-VAS scores (rho = 0.43 and 0.42 respectively, *p <* 0.01), WOMAC Stiffness scores (rho = -0.32 and -0.28 respectively, *p <* 0.05), WOMAC Physical Function scores (rho = -0.43 and -0.38 respectively, *p <* 0.01), and WOMAC Total scores (rho = -0.42, *p <* 0.01 and -0.36, *p < *0.05 respectively). The 2MWT and 6MWT were significantly correlated with each other (rho = 0.86, *p <* 0.01).

**Conclusions:** This study supports the validity of the 2MWT and 6MWT as measures of walking capacity in the TJA in-patient rehabilitation population. Given the fast-paced hospital environment, the 2MWT may be the preferred test, as it requires less test administration time.

# Abstract: A027 – MEASURING CHANGE IN LOWER EXTREMITY FUNCTION IN PATIENTS ATTENDING AN OUTPATIENT POSTOPERATIVE KNEE REPLACEMENT CLASS

**Published:** 2011-07

**Authors:** AV Wainwright, V van Osnabrugge, DM Kennedy, P Stratford, M Rachevitz

**Affiliations:** Holland Orthopaedic & Arthritic Centre of Sunnybrook Health Sciences Centre, Toronto; Department of Physical Therapy, University of Toronto; School of Rehabilitation Science, McMaster University, Hamilton, ON

**Purpose/Objectives & Rationale:** To describe change in lower extremity function in participants post primary total knee replacement (TKR) attending a group class and to examine the relative responsiveness of the Patient Specific Functional Scale (PSFS), Lower Extremity Functional Scale (LEFS) and a timed Stair Test (ST).

**Relevance to Physiotherapy Practice:** No agreement exists concerning the best form of rehabilitation following TKR; higher quality trials with standardized outcome assessment methods are needed.

**Materials and Methods:** Participants attended an outpatient physiotherapy class 2 times per week for 5-6 weeks. The class (10-15 patients) focused on strength, mobility, balance and functional retraining. 73 consecutive patients, mean age 68.7 (SD 7.9), were administered the LEFS, PSFS, P4 pain measure and ST at admission and discharge.

**Analysis:** Using Stata version 10.1, we calculated change scores, 95% confidence intervals (CI) and the Standardized Response Mean (SRM) to estimate responsiveness. To account for dependency in data among the outcome measures, we applied a bootstrap procedure to estimate the CI on the SRM differences between the measures.

**Results:** Statistically significant changes were observed for the outcome measures (mean change scores: LEFS 20.89; PSFS 22.51; P4 8.25 and Stair Test 32.04 seconds). The SRMs for the measures were PSFS 4.35, LEFS 2.00, and Stair test 2.00. The PSFS SRM was 2.17 times more responsive than either the LEFS (95% CI, 1.71, 2.71) and Stair test (95% CI, 1.57, 2.37).

**Conclusions:** Patients attending a short group based outpatient TKR class demonstrated clinically important changes in pain and lower extremity function. The PSFS is a feasible patient-centred responsive measure to use in this population.

# Abstract: A028 – THE IMPACT OF NINTENDO WII™ FIT ON LOWER EXTREMITY FUNCTION OF OUTPATIENTS FOLLOWING TOTAL KNEE REPLACEMENT: A PHASE II TRIAL

**Published:** 2011-07

**Authors:** V Fung, J Shaffer, E Chung, A Ho, M Gomez

**Affiliations:** St. John's Rehab Hospital, Toronto, ON

**Purpose/Objectives & Rationale:** This study investigated whether Nintendo WiiTM Fit (WF) was an acceptable adjunct to outpatient rehabilitation following total knee replacement (TKR).

**Relevance to Physiotherapy Practice:** Video games are innovative therapy adjuncts in physical rehabilitation, which may improve functional outcomes. There is limited research investigating benefits of video games in musculoskeletal rehabilitation.

**Materials and Methods:** This was a randomized controlled trial. Fifty outpatients following TKR, attending physiotherapy (PT) twice weekly, were randomized to either a study (*n =* 27) or control (*n =* 23) group. The study group received 15 minutes of WF activity, and the control group 15 minutes of lower extremity (LE) exercise, additionally to each PT session. Participants were assessed on admission and bi-weekly until discharge.

**Analysis:** *T-*test analyses were used to compare changes between initial assessment and discharge between groups, with a *p < *0.05 considered significant. Mean change of Lower Extremity Functional Scale (LEFS) scores was used to calculate Cohen's d for effect size of WF intervention on LE function.

**Results:** There were 17 males (34%) and 33 females (66%), mean age of 68.0±11.1 years. There were no significant difference between groups in demographics, admission timelines or change in LEFS score from admission to discharge (*p =* 0.079). Cohen's d (0.48) indicates a medium effect size for WF intervention on LE function.

**Conclusions:** Although results were not significant in this phase II study, change in LEFS scores between groups might be significant in a similar trial with a larger sample. These results indicate that as a physiotherapy adjunct, WF can potentially improve LE functional outcomes in outpatients following TKR.

# Abstract: A030 – DETERMINING THE EFFECTS OF WALKING POLES ON THE KNEE ADDUCTION MOMENT USING INSTRUMENTED NORDIC WALKING POLES

**Published:** 2011-07

**Authors:** DJ Bechard, TB Birmingham, A Zecevic, TR Jenkyn

**Affiliations:** * Wolf Orthopaedic Biomechanics Laboratory; † School of Health Studies, Faculty of Health Science, The University of Western Ontario, London, ON

**Purpose/Objectives & Rationale:** (1) To determine how walking poles affect dynamic knee joint loading (knee adduction moment), (2) To determine the magnitude of the moment created by the pole about the knee.

**Relevance to Physiotherapy Practice:** Assistive walking devices are commonly used by individuals with disability in attempts to maintain independence, improve function, enhance safety, and protect joints. Nordic walking poles are a popular recreational activity suggested as an option for individuals with knee osteoarthritis to unload the joint and help maintain reasonable levels of physical activity.

**Materials and Methods:** We assessed 3-dimensional kinematics and kinetics during walking of two instructors deemed experts in the use of Nordic walking poles. We randomly allocated the order of walking trials with and without poles and controlled the walking speed to ±2.5%. We considered the external knee adduction moment and pole moment the primary variables. We also analysed frontal plane lever arm, trunk lean, and ground reaction forces.

**Analysis:** We compared the individual changes in knee adduction moments to previously reported minimal detectable changes. We also compared averages and standard deviations of all variables.

**Results:** The pole provided a substantial moment about the knee (1.64±0.22 BW*Ht). This moment was not met by reduction in the knee adduction moment (1.85±0.87 vs. 2.63±1.40 BW*Ht). The altered body mechanics caused by walking with the poles increase both lever arm (4.1±1.6 vs. 4.6±1.0 cm) and medial GRF (0.06±0.01 vs. 0.08±0.01 %BW).

**Conclusions:** These findings suggest that although walking with Nordic poles can affect the moments about the knee, they do not directly lead to decreases in dynamic knee joint load.

# Abstract: A031 – TOE-OUT AND TRUNK LEAN DURING PROLONGED WALKING IN PATIENT WITH MEDIAL COMPARTMENT KNEE OSTEOARTHRITIS AND HEALTHY CONTROLS

**Published:** 2011-07

**Authors:** DJ Bechard, TB Birmingham, A Zecevic, RR Giffin, TR Jenkyn

**Affiliations:** * Wolf Orthopaedic Biomechanics Laboratory, The University of Western Ontario; † School of Health Studies, Faculty of Health Science, The University of Western Ontario, London, ON

**Purpose/Objectives & Rationale:** (1) To compare time-varying behaviour of maximum toe-out angle and maximum lateral trunk lean during 30 minutes of prolonged treadmill walking in patients with medial compartment knee osteoarthritis and healthy adults, and (2) To explore correlations with perceived level of pain.

**Relevance to Physiotherapy Practice:** Increasing toe-out angle and lateral trunk lean towards the stance limb are proposed as potential compensatory gait mechanisms adopted by patients with medial compartment knee osteoarthritis in response to high mechanical knee joint loading.

**Materials and Methods:** We evaluated 3-dimensional kinematics during 30 minutes of treadmill walking in 20 patients with knee osteoarthritis and 20 healthy adults with no knee complaints. We assessed maximum toe-out angle, maximum lateral trunk lean angle, and perceived levels of pain intermittently.

**Analysis:** We compared groups over time using a 2-way repeated measure ANOVA.

**Results:** Toe-out was lower (*p =* 0.04) in patients with knee osteoarthritis (6.7 ± 5.8) than controls (10.3 ± 5.0). Toe-out changed over time (*p =* 0.02), but not in a systematic way, and there was no significant interaction between group and time. Trunk lean was higher (*p =* 0.03) in patients with knee osteoarthritis (2.0 ± 2.2) than controls (0.6 ± 1.3). However, it did not change over time and there was no interaction between group and time. Only trunk lean was significantly correlated to pain (*r* = 0.64 to 0.65).

**Conclusions:** Although toe-out and trunk lean during walking are different than healthy controls, they do not serve as acute compensatory mechanisms during a single bout of prolonged walking in patients with knee osteoarthritis.

# Abstract: A032 – COMPARISON OF NORDIC WALKING DURING TREADMILL AND OVER-GROUND WALKING CONDITIONS

**Published:** 2011-07

**Authors:** J Appleby, M Carr, M Haire, C Newman

**Affiliations:** School of Physiotherapy, Dalhousie University, Halifax

**Purpose/Objectives & Rationale:** The purpose of the study was to compare the physiologic responses of Nordic Walking on a specially designed treadmill and Nordic Walking on a level over-ground surface.

**Relevance to Physiotherapy Practice:** A specially designed treadmill with designated poling decks would allow people to Nordic Walk indoors to avoid inclement weather.

**Materials and Methods:** Thirteen subjects performed two, 1600 m over-ground Nordic Walking trials at a self-selected pace. Subjects then completed two, 1600 m trials on the Hammer Nordic Walking Treadmill at the walking speed of their over-ground walking trials. Oxygen uptake, heart rate and perceived exertion were measured during each trial.

**Analysis:** Caloric expenditure was calculated using the mean oxygen uptake. Analysis of variance showed no differences in any variable between the trails in each condition. Therefore, the mean and standard deviation for a particular variable was calculated using the observations from both trials. A Mann-Whitney *U* test was used to analyze the RPE scores. Statistical significant was accepted as *p < *0.05.

**Results:** The mean heart rate, oxygen uptake and caloric expenditure during treadmill walking were significantly lower than during the over-ground condition. Perceived exertion for over-ground walking was greater than that for treadmill walking. The narrow walking and poling decks on the treadmill made it difficult for subjects to place their poles correctly and maintain a consistent walking pattern. This would decrease the contribution of the arm muscles to overall oxygen consumption.

**Conclusions:** The Hammer Nordic Walking XTR Treadmill^®^ does not replicate the physiologic stress of over-ground Nordic Walking. Increasing the width of the decks could eliminate the discrepancy.

# Abstract: A034 – COMPUTATIONAL MODELING OF THE WEIGHT-BEARING JOINT CONTACT FORCES AT THE KNEE: TOWARD AN UNDERSTANDING OF THE ALTERED KNEE JOINT MECHANICS IN CHILDREN WITH CEREBRAL PALSY

**Published:** 2011-07

**Authors:** T Flynn, C Mechefske, L Pelland

**Affiliations:** Queen's University, School of Rehabilitation Therapy and Department of Mechanical and Materials Engineering, Kingston, ON

**Purpose/Objectives & Rationale:** Early onset of knee pain with weight-bearing activities is common in children with cerebral palsy. To investigate this clinical issue, we use a computational model to simulate the dynamic interplay between muscle coordination and joint contact forces at the knee. The goal of this study was to demonstrate the predictive validity of the model.

**Relevance to Physiotherapy Practice:** Reliable estimates of joint contact forces would provide a meaningful outcome to inform therapeutic strategies that would optimize both lower limb function and knee joint articular development.

**Materials and Methods:** The model includes tibio- and patello-femoral joints, 47 muscular and 12 directional joint contact forces. Ground reaction force, motion capture data, and anthropometric geometry are used to satisfy a force, moment, and geometry balance at each joint. This solution is found by minimizing the energy output of the system. Arthrokinematic accuracy of the model was validated against published data. Reliability of joint contact forces was validated against in vitro measurements reported for adults.

**Analysis:** Curve fitting analysis to discriminate knee joint contact profiles, and associated muscle moments, over normalized gait cycles for 3 adults: 1 healthy, 1 with moderate radiographical knee osteoarthritis (OA) and 1 with severe OA.

**Results:** Large compression (3.2xBW) and anterior-posterior shear (0.8xBW) forces in severe OA during stance, with large knee extensor (1.5xBW) and medial hamstring (0.75XBW) moments. Respective joint contact values were between 0.6-0.8xBW and 0.4xBW for healthy and moderate OA knees. Lateral gastroc moments was low (0.125xBW) in both OA knees, as compared to healthy knee (0.5xBW).

**Conclusions:** The model reliably discriminates between knee status and associated muscle activity profiles. Next steps will be to implement in paediatric populations.

# Abstract: A036 – “YOU HAVE TO THINK ABOUT WHY YOU'RE DOING IT”: PEDIATRIC PHYSIOTHERAPISTS' USE OF THE NINTENDO WII/WIIFIT

**Published:** 2011-07

**Authors:** D Levac, P Miller

**Affiliations:** * School of Rehabilitation Science, Institute of Applied Health Sciences, McMaster University; † School of Social Work, McMaster University and School of Rehabilitation, McMaster University Institute of Applied Health Sciences, McMaster University, Hamilton, ON

**Purpose/Objectives & Rationale:** The Nintendo Wii and WiiFit are entertaining and accessible virtual reality (VR) gaming consoles that allow the user to control games by means of movement and posture. The purpose of this study is to explore and describe physiotherapists' perspectives on use of the Wii/WiiFit within their interventions for children with acquired brain injury (ABI).

**Relevance to Physiotherapy Practice:** The potential of VR video games to improve motor skill performance is a rapidly developing area of practice and research.

**Materials and Methods:** Participants included six physiotherapists on the Brain Injury Rehabilitation Team at a children's treatment centre, each with approximately 18 months experience using the Wii/WiiFit. A descriptive qualitative approach using 1:1 in-depth semi-structured interviews was employed.

**Analysis:** Qualitative content analysis was used to analyze the interviews. Two investigators independently read and coded interview transcripts to capture key thoughts and concepts, and collaboratively developed themes based on related codes. Member checking was conducted to establish credibility of the findings.

**Results:** The following five themes are identified and explored: establishing intervention goals, consideration of child characteristics, perceived value of the technology as a therapeutic intervention, challenges related to integration in practice, and impacts of technology use on physiotherapist behaviour. Using the Wii/WiiFit adds a new dimension to therapy, altering what the physiotherapist says and does with the child.

**Conclusions:** VR video games are increasingly being integrated into pediatric rehabilitation. This research is unique in exploring physiotherapist perspectives on the impact of their use on clinical practice, informing our understanding of the role of this emerging technology within physiotherapy.

# Abstract: A037 – TWO ARE BETTER THAN ONE: MULTIMODAL STIMULI ALTER MOVEMENT PERFORMANCE

**Published:** 2011-07

**Authors:** CM Glazebrook, C Ko, L Tremblay, TN Welsh

**Affiliations:** * Faculty of Kinesiology and Recreation Management, University of Manitoba, Winnipeg; † Faculty of Physical Education and Health, University of Toronto, Toronto

**Purpose/Objectives & Rationale:** To assess movement planning and execution in the presence of combined visual-auditory information when the secondary modality provides either valid (spatially coincident) or invalid (spatially discordant) information.

**Relevance to Physiotherapy Practice:** Physiotherapists use multiple modalities to engage patients and enhance the learning and re-learning of movements. While combined visual, auditory, and tactile cues are commonly utilized in practice, the impact of a secondary modality on movement processes is poorly understood.

**Materials and Methods:** Twelve healthy young adults performed reaching movements to either visual or auditory targets. On most trials, a secondary stimulus in the alternate modality was presented concurrently with the primary (target) stimulus. The secondary stimulus either provided valid or invalid target location information (0, 8, or 16 degrees visual angle of separation). Spatiotemporal characteristics of the movements were recorded using three-dimensional motion analysis.

**Analysis:** A 2 Modality (Visual, Auditory) by 4 Condition (Alone, Valid [0 degrees], Invalid-near [8 degrees], Invalid-far [16 degrees]) repeated measures ANOVA was used to assess performance and kinematic measures.

**Results:** Participants initiated responses earlier with concurrent visual and auditory information, regardless of spatial location. Movement trajectories and endpoints were biased towards the secondary stimulus only when aiming to auditory target locations.

**Conclusions:** A secondary modality influenced movement parameters only when the primary modality provided less precise spatial information. This finding supports the notion that a secondary stimulus modality may provide benefit for patients with sensory disturbances who are in the process of learning and re-learning movements. Future research will directly examine the impact of multiple modalities on reaching movements in these populations.

# Abstract: A039 – SHOULD CORRECTED OR CHRONOLOGICAL AGE BE USED AT 18 MONTHS TO IDENTIFY PRETERM INFANTS AT RISK OF LONGTERM MOTOR IMPAIRMENT?

**Published:** 2011-07

**Authors:** MJ Rogers, A Synnes, A Butt

**Affiliations:** Children's and Women's Health Center of BC, Neonatal Follow Up Clinic, Vancouver

**Purpose/Objectives & Rationale:** To correlate Chronological Age (CA) versus Chronological Corrected Age (CCA) motor test scores at 18 months and 3 years with motor outcomes at 4.5 years in Extremely Low Gestational Age (ELGA) children and evaluate the clinical consequences.

**Relevance to Physiotherapy Practice:** Children born ELGA are at significantly increased risk for longterm motor coordination impairment.Early identification of children at greatest risk for motor impairment will facilitate appropriate and timely referrals for physiotherapy intervention.

**Materials and Methods:** 122 children born ELGA (62 boys; 60 girls,born less than 29 weeks gestational Age (GA); median 25.5 weeks) completed motor assessments between 2002 and 2009 using the Peabody Developmental Motor Scales (PDMS II) at 18 months and 3 years and the Movement Assessment Battery for Children (MABC) at 4.5 years.

**Analysis:** Spearman Correlations were used to compare CCA and CA PDMS II Total Motor Quotient (TMQ) scores at 18 months and 3 years of age to the MABC percentile score at 4.5 years. An MABC cutoff score of less than 5th percentile was considered clinically significant. Sensitivity, specificity, positive and negative predictive values were calculated.

**Results:** PDMS II scores using CA at 18 months and 3 years correlated slightly better than CCA scores(18m: *r* = 0.582 vs. *r* = 0.512; 3y: *r* = 0.693 vs. *r* = 0.640). Sensitivity: 18 months (CCA) 0.26 vs. (CA) 0.76. Specificity: 18 months (CCA) 0.97 vs. (CA) 0.70. PPV: (CCA) 0.83 vs. (CA) 0.54. NPV: (CCA) 0.74 vs. (CA) 0.87.

**Conclusions:** PDMS II scores using CA at 18 months and 3 years correlate better with 4.5 MABC outcomes than CCA. The low sensitivity of using CCA, especially at 18 months, may result in children not receiving necessary physiotherapy intervention if there is not routine follow up beyond 3 years.

# Abstract: A040 – DEVELOPMENTAL PROFILES OF MULTI-JOINT UPPER LIMB POSITION SENSE

**Published:** 2011-07

**Authors:** L Pelland, C Henderson, T Herter, S Scott

**Affiliations:** School of Rehabilitation Therapy and Center for Neuroscience Studies, Queen's University, Kingston, ON

**Purpose/Objectives & Rationale:** The goal of this study was to quantify the typical change in multi-joint position sense of the upper limb (UL) in children, 5 to 18 years old. This profile will provide a developmental reference to reliably quantify the performance deficits in children with hemiplegia.

**Relevance to Physiotherapy Practice:** Measurement of position sense in a large cohort of able-bodied children will provide clinicians with robust data to reliably identify children with UL proprioceptive deficits and to design intervention plans that are developmentally supportive.

**Materials and Methods:** The KINARM exoskeleton, a bilateral robot for planar arm movements, was used. One UL was passively moved to one of 9 spatial positions and participants were asked to match this position with the other arm without visual feedback. All spatial positions were tested in each trial block, and 6 blocks were completed. Three outcome parameters were calculated: trial-to-trial variability of the active hand, contraction/expansion of the overall spatial area matched by the active hand, and systematic shifts, or constant errors in position between the active and passive hands.

**Analysis:** Growth curves were constructed for each parameter, plotting mean individual scores as a function of chronological age (CA). Linear regression models were used to assess influence of CA on each parameter; effects of sex and handedness were evaluated as co-variates.

**Results:** Variability and spatial contraction decreased with CA (*p <* 0.001), with significant effects still present at 18 years. No systematic errors, or effects of sex and handedness were observed.

**Conclusions:** Results provide evidence of the continued development of multi-joint position sense into late adolescence.

# Abstract: A042 – HEALTHY PHYSICAL AGING: NORMATIVE DATA FOR THE DE MORTON MOBILITY INDEX (DEMMI)

**Published:** 2011-07

**Authors:** NA de Morton, EM Macri, MC Ashe, KM Khan

**Affiliations:** * Musculoskeletal Research Centre, La Trobe University, Northern Health, Melbourne, Victoria, Australia; † Centre for Hip Health and Mobility, Vancouver Coastal Health Research Institute, University of British Columbia, Vancouver

**Purpose/Objectives & Rationale:** The de Morton Mobility Index (DEMMI) is a valid and reliable instrument that measures the mobility of older adults across clinical settings. The DEMMI consists of 15 hierarchical items with scoring from 0-100 where 100 denotes the highest mobility level. Establishing norms is vital in health instrument development. Therefore, our aim was to obtain age- and gender-stratified normative data for the DEMMI for healthy community dwelling older adults.

**Relevance to Physiotherapy Practice:** The management of mobility is a key domain within the physiotherapy scope of practice. Normative data provides a reference when measuring mobility and in directing therapeutic interventions.

**Materials and Methods:** Eligible participants were aged 60 years+ and living independently in Metro Vancouver, British Columbia with no known condition affecting mobility. A physiotherapist conducted the DEMMI assessment. Questionnaires were also administered regarding quality of life, disability and falls risk.

**Analysis:** We provided descriptive data of the sample and mean (SD) DEMMI scores by gender and age categories: 60-69, 70-79, 80-89 and 90+ years.

**Results:** 64 participants were assessed, 14 men and 50 women. Mean score for men was 84.6 (10.2) and women, 83.5 (11.0). Mean DEMMI scores systematically declined with increasing age: 88.6 (12.2), 83.5 (9.1) and 79.0 (11.5) for 60-69 years (*n =* 15), 70-79 years (*n =* 35) and 80-89 years (*n =* 14) respectively. These differences in DEMMI scores were not statistically significant.

**Conclusions:** Declining DEMMI mobility scores with increasing age are consistent with the known physiological effects of aging. Further data collection is planned to increase confidence in these normative data point estimates.

# Abstract: A043 – COMPARING THE CONTENT OF PARTICIPATION INSTRUMENTS USING THE INTERNATIONAL CLASSIFICATION OF FUNCTIONING, DISABILITY AND HEALTH

**Published:** 2011-07

**Authors:** VK Noonan, JA Kopec, J Singer, L Noreau, MF Dvorak

**Affiliations:** * Rick Hansen Institute, Vancouver; † School of Population and Public Health, University of British Columbia, Vancouver; ‡ School of Population and Public Health, University of British Columbia, Vancouver; § Rehabilitation Department, Laval University, Quebec City; ¶ Division of Spine, Department of Orthopaedics, University of British Columbia, Vancouver

**Purpose/Objectives & Rationale:** Instruments have recently been developed to measure participation using the International Classification of Functioning, Disability and Health (ICF). Few studies have examined the content of these instruments to determine how participation has been operationalized. The purpose of this study was to compare the content of participation instruments using the ICF classification.

**Relevance to Physiotherapy Practice:** Participation is an important rehabilitation outcome and information on the content can assist physiotherapists in selecting an appropriate instrument.

**Materials and Methods:** A systematic literature search identified instruments that assess participation according to the ICF. Instruments were included if the domains contain content from a minimum of three ICF chapters in the activities and participation (A/P) component. Meaningful concepts in each question were identified and then linked to ICF categories.

**Analysis:** A descriptive analysis was conducted and included reporting the ICF chapters covered and the context in which the A/P component categories were operationalized.

**Results:** Eight instruments were included and 1351 meaningful concepts were identified. All the instruments cover six to eight of the nine chapters in the A/P component. Two instruments have questions which do not contain any meaningful concepts related to the A/P component. The instruments differ in whether the A/P component categories are asked in the context of other ICF components (body functions, environmental factors) and health.

**Conclusions:** Linking the meaningful concepts in the participation instruments to the ICF classification provided an objective and comprehensive method for analyzing the content. There are differences in how participation is operationalized which need to be considered when selecting an instrument.

# Abstract: A044 – ASSESSING THE INTERNATIONAL CLASSIFICATION OF FUNCTIONING, DISABILITY AND HEALTH (ICF) CONCEPT OF PARTICIPATION: COMPARING 3 INSTRUMENTS IN INDIVIDUALS WITH SPINAL CORD INJURY

**Published:** 2011-07

**Authors:** VK Noonan, JA Kopec, J Singer, L Noreau, MF Dvorak

**Affiliations:** * Rick Hansen Institute, Vancouver; † School of Population and Public Health, University of British Columbia, Vancouver; ‡ School of Population and Public Health, University of British Columbia, Vancouver; § Rehabilitation Department, Laval University, Quebec City; ¶ Division of Spine, Department of Orthopaedics, University of British Columbia, Vancouver

**Purpose/Objectives & Rationale:** Participation is defined as the involvement in life situations and participation restrictions contribute to disability. Instruments assessing participation have not been compared. This study compared the reliability and construct validity of 3 participation instruments in individuals with spinal cord injury (SCI).

**Relevance to Physiotherapy Practice:** Participation is a meaningful outcome following rehabilitation but more information is needed to help physiotherapists select an appropriate instrument.

**Materials and Methods:** Patients (*n =* 145) were contacted by mail and completed 3 participation instruments: World Health Organization Disability Assessment ScheduleII (WHODASII); Impact on Participation and Autonomy (IPA); and Participation Measure-Post Acute Care (PM-PAC). A second questionnaire was mailed 7 days later.

**Analysis:** Floor and ceiling effects described the proportion of subjects with the worst and best scores. Test-retest reliability was evaluated using intraclass correlation coefficients (ICC) and the minimal detectable change (MDC) was calculated. Validity was assessed by correlating similar domains among the 3 instruments using Spearman's rho. Regression models were used to test hypotheses regarding group-effects (e.g. demographics).

**Results:** Ceiling effects were common among all instruments. The IPA had the highest internal consistency and ICCs, with all the values > 0.70. The MDCs as a percentage of the total scale score range were primarily 20% to 30% for the 3 instruments. Correlations among similar domains ranged between rho = 0.50 to 0.69. The IPA and PM-PAC had the most hypotheses supported.

**Conclusions:** The WHODASII, IPA and PM-PAC have similar measurement properties in individuals with SCI. Ceiling effects suggest these instruments should be used in persons with moderate/severe participation restrictions. Future research should establish the minimal clinically important differences.

# Abstract: A049 – MRI-BASED 3D SHAPE ANALYSIS OF THIGH MUSCLES: PATIENTS WITH CHRONIC OBSTRUCTIVE PULMONARY DISEASE VERSUS HEALTHY ADULTS

**Published:** 2011-07

**Authors:** B Ghanbari, G Hamarneh, N Changizi, AD Ward, WD Reid

**Affiliations:** * Research Graduate Programs in Rehabilitation Sciences, University of British Columbia, Vancouver; † School of Computing Science, Simon Fraser University, Burnaby, BC; ‡ Robarts Research Institute, University of Western Ontario, London, ON; § Muscle Biophysics Laboratory, Vancouver Coastal Health Research Institute, Department of Physical Therapy, University of British Columbia, Vancouver

**Purpose/Objectives & Rationale:** Muscle weakness and atrophy is well-recognized in people with COPD, however, its regional distribution within a muscle has not been studied. The purpose of this study was to perform 3D shape analysis of images obtained from magnetic resonance imaging (MRI) in order to detect shape abnormalities of individual thigh muscles in people with COPD compared to healthy people.

**Relevance to Physiotherapy Practice:** MRI imaging usually provides cross-sections of thigh muscles. Reconstruction of these cross-sections to 3-D shaped muscles can potentially reflect regional atrophy.

**Materials and Methods:** Twenty COPD patients and twenty healthy adults (55-79 years) underwent MRI that provided 100 cross-sections of the thigh. Segmentation of individual knee extensor and flexor muscles on each cross-section was performed. Using specialized software, the 3D shape of each muscle was constructed. Eight shape descriptors were determined for each thigh muscle and regionally (for portions of the muscle).

**Analysis:** Two-tailed *t-*tests with modified Bonferroni correction were used to compare group differences.

**Results:** Global analysis showed differences between groups (*p <* 0.01) for vastus intermedius (5 descriptors), semimembranosus (4 descriptors), and rectus femoris (1 descriptor). Regional shape differences (*p <* 0.01) between groups were found for all knee extensors and semimembranosus. A support vector machine classifier provided 95% to 100% accuracy in differentiating healthy from COPD muscles.

**Conclusions:** We found that coincident with atrophy, muscle shape is altered; knee extensors were affected more than knee flexors in COPD. Further research is required to explore the underlying mechanisms of muscle shape discrepancies in COPD, which will facilitate the design of more specific therapeutic interventions that address muscle weaknesses and imbalance.

# Abstract: A050 – CLINICAL REASONING AND PRACTICE PATTERNS OF CANADIAN PHYSIOTHERAPISTS MOBILIZING PATIENTS WITH EXTERNAL VENTRICULAR DRAINS

**Published:** 2011-07

**Authors:** C Hale, K Wong, A Calvin, A Rnic, B Tobali, C Duncan, C Hawke, J Brown, C Heck, C Cott

**Affiliations:** * University of Toronto, Department of Physical Therapy; † University Health Network, Toronto

**Purpose/Objectives & Rationale:** To describe mobilization practices of Canadian physiotherapists (PTs) treating patients with EVDs and to determine if practice patterns are influenced by clinical experience.

**Relevance to Physiotherapy Practice:** Data may be used for development of best practice mobility guidelines with this population. Differences in clinical reasoning skills based on level of experience may direct the focus of education towards reducing gaps in knowledge base.

**Materials and Methods:** A quantitative, descriptive, cross-sectional study design was utilized. An online questionnaire sent to participants in March 2010 collected information on demographics, clinical practices, and practice barriers specific to patients with EVDs. Additionally, subjects were asked to analyze three case studies and identify rationale for their mobilization treatment choices.

**Analysis:** Descriptive statistics were used to describe demographics. Mean ± standard deviations were calculated for ranking factors. *T*-tests were performed to determine between group differences.

**Results:** The majority of respondents (*n =* 29) agreed that medically stable patients with EVDs should be mobilized in-bed (96%), edge-of-bed (78%) and out-of-bed (74%). Clinical experience and safety concerns were most commonly cited as factors guiding practice. More experienced PTs were more likely to use aggressive mobilization practices. Regardless of experience, intracranial pressure was ranked most important and saturation of oxygen as the least important factor considered. A higher percentage of more experienced PTs ranked the Glasgow Coma Scale and mean arterial pressure as important factors that would guide their mobilization practices.

**Conclusions:** Conservative mobility options were more readily employed by PTs for borderline or medically unstable patients. Level of experience appears to be related to clinical reasoning and aggressiveness of mobilization practices for patients with EVDs.

# Abstract: A052 – CARE FOR RURAL PATIENTS WITH A HIP FRACTURE: WHAT DOES INFORMATION FLOW REVEAL ABOUT PRESSURES IN THE HEALTH CARE SYSTEM?

**Published:** 2011-07

**Authors:** H Johnson, BM Chesworth, D Forbes, J Elliott, JL Wells, P Stolee

**Affiliations:** * Health and Rehabilitation Sciences, University of Western Ontario, London, ON; † University of Alberta, Edmonton, AB; ‡ University of Western Ontario, London, Ontario; § St. Josephs Health Care, London, Ontario; ¶ University of Waterloo, Ontario

**Purpose/Objectives & Rationale:** Transitions across the care continuum are of increasing interest to clinicians, researchers and policy makers. Patients with hip fracture typically transition through several care environments during recovery, as their care is handed off by numerous health care professionals. While care providers strive to make the journey smooth and seamless, communication and information flow can become fragmented.This study examined information exchange at care transitions and the associated care hand offs of older patients following a hip fracture in rural Ontario.

**Relevance to Physiotherapy Practice:** Physiotherapists are integral members of the health care team and intimately involved in the flow of information during patient transitions through the health care system.

**Materials and Methods:** Utilizing an ethnographic approach, 11 patients were followed from acute care through each post-acute care setting. Semi-structured interviews, observation and document review were completed with patients, family caregivers and health care providers to examine expressed information needs and actual information exchange.

**Analysis:** Data were analyzed through a critical, gerontological focused lens for themes relevant to information exchange.

**Results:** The discharge destination proved to be a key decision requiring information exchange. This decision occurred in a health care system under pressure. There were facilitators and challenges associated with the rural health care context.

**Conclusions:** While systems exist to provide for information flow, rural elderly hip fracture patients and their families can experience challenges in care transitions due to pressures in the current health care system. An environment of trust and respect with health care providers facilitates transitions.

# Abstract: A055 – PELVIC FLOOR MUSCLE MORPHOLOGY AND FUNCTION IN WOMEN WITH AND WITHOUT PROVOKED VESTIBULODYNIA EVALUATED USING 3D/4D ULTRASOUND IMAGING

**Published:** 2011-07

**Authors:** S Thibault-Gagnon, L McLean, C Pukall, C Goldfinger, S Chamberlain

**Affiliations:** * Queen's University, School of Rehabilitation Therapy; † Queen's University, Department of Psychology; ‡ Queen's University, Department of Obstetrics and Gynaecology, Kingston, ON

**Purpose/Objectives & Rationale:** Although alterations in pelvic floor muscle function are thought to be associated with provoked vestibulodynia (PVD), no study has investigated differences in the morphology of the PFMs between women with and without PVD. The aim of this study was to investigate differences in PFM morphology and function between women with and without PVD using ultrasound imaging (USI).

**Relevance to Physiotherapy Practice:** Currently, clinical assessment of the PFMs in women with PVD is subjective and discrete. USI may be a useful tool for PFM assessment and training.

**Materials and Methods:** Ten women with PVD and 10 aged-matched controls participated in this case-control study. Transperineal USI of the levator hiatus (LH) while participants kept their PFMs relaxed, performed a maximal PFM contraction, and performed a maximal Valsalva. Measurements of the dimensions of the LH (anteroposterior and transverse diameters, area, and circumference) and the length of the puborectalis muscle were made off-line.

**Analysis:** Separate two-way ANOVAs including group and task main effects and group by task interactions were performed for each measure (α = 0.05).

**Results:** In both groups, compared to measures obtained at rest, significant reductions in all measures were seen during contraction, and significant increases were seen during Valsalva. The anteroposterior and transverse diameters, and the area of the LH were significantly smaller in women with PVD as compared to women in the control group. No group by task interactions were found.

**Conclusions:** Women with PVD may have smaller levator hiatuses than women without vulvar pain, but they do not appear to demonstrate differences in muscle shortening or lengthening.

# Abstract: A056 – THE IMPACT OF PELVIC FLOOR MUSCLE EXERCISE ON THE MORPHOLOGY AND FUNCTION OF THE CONTINENCE SYSTEM IN WOMEN

**Published:** 2011-07

**Authors:** L McLean, E Gentilcore-Saulnier, K Baker, MA Harvey, E Sauerbrei

**Affiliations:** Queen's University, School of Rehabilitation Therapy, Kingston, ON

**Purpose/Objectives & Rationale:** Pelvic floor muscle (PFM) training is effective at alleviating symptoms of stress urinary incontinence in approximately 50% of women however its mechanism is not clear.

**Relevance to Physiotherapy Practice:** By determining the morphological and functional changes in the continence system induced by PFM exercise, we may be able to streamline interventions by identifying women who will optimally benefit from PFM strengthening.

**Materials and Methods:** Twenty women with stress urinary incontinence volunteered to participate in a twelve-week program of PFM strengthening exercises. Ultrasound imaging was used to measure the morphology of the levator hiatus and urethral sphincters, the resting position of the bladder neck, and urethral mobility during cough and Valsalva maneuvers. These measures, along with the administration of the Urogenital Distress Inventory (UDI) and Incontinence Impact Questionnaire (IIQ), were performed before and after 12 weeks of exercise training.

**Analysis:** Separate repeated-measures analyses of variance (α = 0.05) were performed for each measure.

**Results:** Compared to pre-treatment measures, after treatment women demonstrated significant improvements on the IIQ but not on the UDI. With training, the women demonstrated significant increases in the cross-sectional area of the urethral sphincter, and significant reductions in urethral excursion during coughing. Surprisingly, after treatment the resting position of the bladder neck was lower and there was an increase in the length of the puborectalis muscle. There was no change in urethral excursion on Valsalva.

**Conclusions:** PFM training increased the size of the urethral sphincter and reduced urethral mobility during coughing while reducing the impact of incontinence on the quality of life of women in our sample.

# Abstract: A057 – DOES THE PRESENCE OF A VAGINAL PROBE ALTER PELVIC FLOOR MUSCLE ACTIVATION IN YOUNG, CONTINENT WOMEN?

**Published:** 2011-07

**Authors:** C Auchincloss, L McLean

**Affiliations:** Queen's University, School of Rehabilitation Therapy, Kingston, ON

**Purpose/Objectives & Rationale:** The purpose of this study was to determine whether pelvic floor muscle (PFM) recruitment changes due to changes in sensory feedback, muscle length or tissue position caused by different vaginal probes used to record surface electromyography (EMG).

**Relevance to Physiotherapy Practice:** Vaginal probes with embedded EMG electrodes may induce changes in muscle recruitment by the very nature of the shape of the probes. Fine-wire electrodes allow us to detect muscle activation parameters without altering the natural position and shape of the PFMs.

**Materials and Methods:** Twelve continent women (30.1±5.4 years), performed PFM maximal voluntary contractions (MVCs) in supine while fine-wire EMG was recorded bilaterally from the PFMs under three conditions: a) fine-wire EMG only, b) fine-wire EMG while a Femiscan™ probe was in situ, and c) fine-wire EMG while a Periform™ vaginal probe was in situ.

**Analysis:** First, the reliability of the fine wire EMG data was tested using intraclass correlation coefficients (ICCs) and coefficients of variation (CV) to ensure that the data were consistent across conditions. Next, an analysis of variance (ANOVA) model was used to detect differences in EMG amplitude recorded between the conditions.

**Results:** The between-trial reliability was excellent, ICC (3,k) = 0.98, (*p <* 0.001) and CV = 13.6%. There were no differences in EMG amplitude across the three conditions (fine-wire 63.4±48.4µV, fine-wire with Femiscan™ 55.3±42.4 µV, fine-wire with Periform™ 59.4±42.2µV, *p =* 0.178).

**Conclusions:** These results suggest that women produce consistent MVCs over time, and that the results are not affected by measurement devices inserted into the vagina.

# Abstract: A058 – SURFACE ELECTROMYOGRAPHY OF THE PELVIC FLOOR MUSCULATURE: VALIDITY OF A NOVEL ELECTRODE DESIGN

**Published:** 2011-07

**Authors:** N Keshwani, L McLean

**Affiliations:** Queen's University, School of Rehabilitation Therapy, Kingston, ON

**Purpose/Objectives & Rationale:** Current intravaginal probes used for recording surface electromyography (sEMG) from the pelvic floor muscles (PFMs) feature large detection surfaces, which increases the risk of recording unwanted activity from nearby muscles (crosstalk), threatening the validity of results. This study compared crosstalk contamination of sEMG recordings from the PFMs using a novel electrode to a commercially available intravaginal probe, the Femiscan.

**Relevance to Physiotherapy Practice:** sEMG is a commonly used tool in women's health physiotherapy, both for the assessment of the PFMs in the research realm, and for biofeedback purposes in the clinical realm.

**Materials and Methods:** Subjects (*n =* 20, average age: 25.9 ± 7.4 years) performed isolated unilateral hip external rotation at 25% of their maximal contractile effort (MVC) with each vaginal electrode in situ.

**Analysis:** Repeated measures ANOVAs (α = 0.05) were performed to investigate differences in sEMG amplitudes between contraction intensities and electrodes. Separate analyses were performed for the right and left PFMs.

**Results:** Effect of Ipsilateral Hip Rotation: At 25% MVC, the Femiscan recorded significantly higher sEMG amplitudes compared to what was recorded with the hip muscles relaxed, whereas the novel electrode did not. Effect of Contralateral Hip Rotation: At 25% MVC, neither the Femiscan nor the novel electrode recorded significantly higher EMG amplitudes compared to what each recorded when the hip was relaxed.

**Conclusions:** When using sEMG biofeedback to assist with PFM training or when studying functional tasks, crosstalk is likely an issue when ipsilateral concurrent hip external rotation is performed at submaximal intensities. This is not the case with our novel electrode.

# Abstract: A059 – PHYSIOTHERAPY SIGNIFICANTLY REDUCES LEAKAGE IN POSTMENOPAUSAL WOMEN WITH OSTEOPOROSIS AND URINARY INCONTINENCE: RESULTS OF A RANDOMIZED CONTROLLED TRIAL

**Published:** 2011-07

**Authors:** MM Sran, P Wilson, P Lieblich, C Dumoulin

**Affiliations:** * BC Women's Hospital & Health Centre, Vancouver; † Université de Montréal, Montreal

**Purpose/Objectives & Rationale:** To assess the effectiveness of physiotherapy for urinary incontinence (UI) compared with a control intervention, for reducing the number of UI episodes and severity of UI, in postmenopausal women aged 55-85 years with osteoporosis or low bone density, and stress, urge or mixed UI.

**Relevance to Physiotherapy Practice:** A recent study found that near 40% of patients presenting to a specialized osteoporosis service reported having UI at least once per week(1). This is important because UI can significantly limit a woman's ability to be physically active and is an independent risk factor for falls and low trauma fractures in older women.

**Materials and Methods:** Participants (*n =* 48) were randomly allocated to either the physiotherapy group (once/week for 12 weeks) or the control group. All measurements were completed at baseline, 14 weeks and 1 year. Outcome assessors were blind to group allocation.

**Analysis:** Intention to treat analysis was conducted. Between-group differences were analyzed using the Mann-Whitney *U* test.

**Results:** At 14 weeks there was a significant difference between groups in the number of leakage episodes on the 7-day bladder diary (*p =* 0.04) and total Urogenital Distress Inventory (UDI) score (*p = *0.04), in favour of the physiotherapy group. At 1 year (46/48 participants completed study to date) there was a significant difference between groups, in favour of the physiotherapy group, for the number of leakage episodes (*p =* 0.04), 24 hour pad test (*p =* 0.01), and UDI (*p =* 0.03).

**Conclusions:** Physiotherapy was effective in reducing the amount and severity of UI in postmenopausal women with osteoporosis and UI. Results were maintained 1 year later.

(1) Sran MM. J Obstet Gynaecol Can 2009.

# Abstract: A071 – EVALUATING PARENTAL STRESS LEVEL AND SATISFACTION AFTER RECEIVING A NEW COMPLEMENT TO PHYSIOTHERAPY INTERVENTION FOR POSITIONAL TORTICOLLIS IN INFANTS

**Published:** 2011-07

**Authors:** P Lopes, E Kennedy, I Gagnon, L Grilli, S McShane, B Mazer, R Damignani, D Feldman

**Affiliations:** Montreal Children's Hospital Physiotherapy Department, Montreal

**Purpose/Objectives & Rationale:** Providing written information about exercises has been shown to reduce stress levels and increase satisfaction with the treatment received. The purpose of this study was to evaluate parental stress level and satisfaction after receiving a new Torticollis Intervention Booklet (TIB).

**Relevance to Physiotherapy Practice:** There are few educational tools with comprehensive information about physical therapy exercises for infants with torticollis. This project was meant to generate evidence on how to best complement physiotherapists' intervention.

**Materials and Methods:** This is a randomized clinical trial conducted at the Montreal Children's Hospital (MCH). Seventy-Three infants referred to physiotherapy for a positional torticollis were recruited and randomized to the Physiotherapy + TIB group (*n =* 37) or to the Physiotherapy + Standard Exercise Sheet group (*n =* 36). Infants received standard physiotherapy treatment and verbal instructions delivered by their own physiotherapists and were assessed 1 and 3 months after their initial visit by a blind evaluator. Outcomes measured were cervical ROM, parental stress level (Parental Stress Index) and parental satisfaction (Measure of Processes of Care).

**Analysis:** Intention to treat analysis was performed. *T-*tests on the change between assessment sessions were performed for each variable.

**Results:** Parents receiving the TIB reported lower stress level (*p =* 0.01) and greater satisfaction related to provider partnership and information delivered (*p <* 0.001) than those receiving standard care.

**Conclusions:** A thorough and well-illustrated booklet accompanying verbal instructions for home exercises in the treatment of PT leads lower parental stress and greater parental satisfaction and may lead to earlier restitution of ROM.

# Abstract: A072 – THE BAYLEY SCALES OF INFANT AND TODDLER DEVELOPMENT IN EXTREMELY LOW BIRTH WEIGHT SURVIVORS AT 18 MONTHS CORRECTED AGE

**Published:** 2011-07

**Authors:** MJ Rogers, A Synnes, J Petrie-Thomas, A Butt

**Affiliations:** Children's and Women's Health Center of BC, Vancouver

**Purpose/Objectives & Rationale:** The Bayley Scales of Infant and Toddler Development 3rd edition (BSITD-III),is commonly used in evaluation of infants at risk for developmental delay. This recent edition includes composite scores (Cognitive, Language and Motor) with subtest scores for Receptive (RC) and Expressive Communication (EC) and Fine (FM) and Gross motor (GM). The Bayley-I (BSID, 1969) and BSID-II(1993) results used Mental (MDI) and Psychomotor Development Index(PDI). The objective of this study is to Describe BSITD-III, BSID-II and BSID-I results in 3 cohorts of extremely low birth weight (ELBW, less than 800g) children seen at 18 months corrected age.

**Relevance to Physiotherapy Practice:** BSITD-III is used for research and clinical evaluation of infants. This study will enable clincians and researchers to compare current and previous studies of high risk populations.

**Materials and Methods:** ELBW toddlers free of severe cerebral palsy, visual impairment able to complete test items between 1984 and 2009 were included. Period 1(1984-1993) included 179 subjects (median birthweight (bwt) 720g (range 480-800g); median gestational age (GA) 25.7 weeks (range 23-33). Period 2(1994-2006) had 163 subjects (median bwt 700 g (425-800g); GA 25.7 wks (range 22-32).Period 3 (2006-2009) had 92 subjects (median bwt 658 g (range 385-800 g); GA 25 wks (range 23-29).

**Analysis:** Paired *t-*tests were used to compare Bayley-III profiles.

**Results:** BSID-I MDI and PDI scores are higher than BSID-II scores consistent with the literature. The Bayley-III profile of Canadian non-impaired ELBW infants shows better cognitive vs. language and better fine vs. gross motor skills, both *p* < 0.0001). RC=EC.

**Conclusions:** In ELBW subjects, a median cognitive score of 100 and motor score of 94, both within the normal range, highlights that results should be verified against Canadian term controls.

# Abstract: A074 – PAIN IN PEOPLE WITH CHRONIC OBSTRUCTIVE PULMONARY DISEASE (COPD)

**Published:** 2011-07

**Authors:** B Ghanbari, L Holsti, DW Reid

**Affiliations:** * Research Graduate Programs in Rehabilitation Sciences, University of British Columbia; † Centre for Community Child Health Research, Child and Family Research Institute, Vancouver; and Department of Pediatrics, University of British Columbia; ‡ Muscle Biophysics Laboratory, Vancouver Coastal Health Research Institute, Department of Physical Therapy, University of British Columbia, Vancouver

**Purpose/Objectives & Rationale:** Pain can adversely affect the health related quality of life (HRQoL) in people with COPD, however; it has not been systematically studied in this patient group. The purpose of this study was to investigate the intensity and interference of pain and its association with physical activity and HRQoL in people with COPD.

**Relevance to Physiotherapy Practice:** Physiotherapists' awareness of factors that limit exercise and activity in people with COPD will facilitate more specific prescription of interventions to improve performance and function.

**Materials and Methods:** Forty seven people with moderate to severe COPD and 47 matched healthy controls (Age: 51-86 years) participated in a cross-sectional study that examined: 1) pain descriptors by the McGill Pain Questionnaire (MPQ); 2) Brief pain Inventory (BPI); 3) modified Tampa Scale for Kinesiophobia (TSK); 4) HRQoL (SF-36); and 5) Community Health Activities Model Program for Seniors (CHAMPS).

**Analysis:** Two-tailed *t-*tests with a modified Bonferroni correction were used to compare groups.

**Results:** Compared to the healthy subjects, people with COPD had higher values of: pain intensity on the MPQ (*p* < 0.001); pain interference on the BPI (*p* < 0.001); bodily pain (*p* < 0.05) and role emotional (*p* < 0.001) domains of SF-36. Energy expenditure for overall and moderate intensity activities were lower in people with COPD compared to the healthy group (*p* < 0.001).

**Conclusions:** People with COPD experience significant pain that should be considered in addition to other factors that limit physical activity. Therapeutic interventions to alleviate pain need to be included in rehabilitation programs.

# Abstract: A075 – THE DURATION OF ANALGESIA INDUCED BY DIFFUSE NOXIOUS INHIBITORY CONTROLS

**Published:** 2011-07

**Authors:** Y Tousignant-Laflamme, S Racette, J Roy, S Marchand

**Affiliations:** Faculty of medicine, School of rehabilitation, University of Sherbrooke, Sherbrooke, QC

**Purpose/Objectives & Rationale:** While the magnitude of diffuse noxious inhibitory controls (DNIC) analgesia, an endogenous pain inhibitory mechanism, has already been documented, no study measured its duration.

**Relevance to Physiotherapy Practice:** Pain inhibitory mechanisms, such as DNIC, are of particular interest in rehabilitation since it is responsible for the analgesia induced by many nonpharmacological treatments such as TENS-acupuncture and manual therapy.

**Materials and Methods:** 20 subjects (10 men and 10 women) underwent a tonic heat pain stimulation with a contact thermode for 2 minutes. Afterwards, each particpant underwent a 2-min cold pressor test (CPT) to activate DNIC (7°C and 12°C). This was followed by another series of tonic heat pain stimulations until pain ratings returned to pre-CPT level. Pain ratings were obtained with a numeric scale.

**Analysis:** In order to quantify the duration DNIC analgesia, we used Kaplan-Meier survival analysis, with median duration as well as 95% confidence intervals. Mean DNIC duration for both CS were compared using student *t-*test.

**Results:** Results showed that DNIC median duration was of 35 min. following the 7°C CPT and 10 min. following the 12°C (*p = *0.01). Maximal DNIC analgesia duration was 115 min. and 75 min. (7°C and 12°C CPT respectively). Furthermore, despite that the 7°C CPT provoked significantly more pain, the magnitude of DNIC analgesia was comparable following both sessions.

**Conclusions:** Overall, the duration of DNIC analgesia was significantly longer following the 7°C CPT. Although some literature suggest that DNIC strength is proportional to the intensity of the conditioning stimulus, we found comparable DNIC analgesia following the two CPT. The implications for rehabilitation are discussed.

# Abstract: A076 – A SURVEY OF CHRONIC MUSCLE PAIN AND OTHER SYMPTOMS IN INDIVIDUALS SUSCEPTIBLE TO MALIGNANT HYPERTHERMIA

**Published:** 2011-07

**Authors:** JK Kozack, DL MacIntyre

**Affiliations:** University of British Columbia, Vancouver

**Purpose/Objectives & Rationale:** Prior to this study, individuals susceptible to malignant hyperthermia were reported to be usually asymptomatic in the absence of inhalation anaesthetics.This study was undertaken to investigate reports of chronic muscle pain, muscle cramping, headaches, and heat stroke in individuals susceptible to malignant hyperthermia, and to determine if there is a difference in the proportion of susceptible versus non susceptible individuals reporting these symptoms.

**Relevance to Physiotherapy Practice:** Physiotherapists may encounter clients with susceptibility to malignant hyperthermia. It is important for physical therapists to understand how susceptibility predisposes them to chronic muscle pain and other symptoms.

**Materials and Methods:** Questionnaires were mailed to all individuals in the North American Malignant Hyperthermia Registry that underwent diagnostic testing between 1995 and 1999. Of those meeting the selection criteria, 106 susceptible and 62 non-susceptible individuals were mailed questionnaires, with response rates of 58 and 45 percent respectively. The questionnaire included the presence and location of chronic muscle pain, and the occurrence of headaches, muscle cramps, and heat stroke.

**Analysis:** The Fisher Exact Test was used to analyze the data.

**Results:** Statistically significant differences (*p <* 0.05) were found in the percent reporting chronic muscle pain between the susceptible and non-susceptible groups: 38 percent versus 14 percent respectively. Muscle cramping was also reported more in the susceptible group (77 percent with *p <* 0.05). No significant differences were found between the two groups reporting headaches or heat stroke.

**Conclusions:** Individuals susceptible to malignant hyperthermia may be symptomatic in the absence of inhalation anaesthetics. They are more likely to report chronic muscle pain and muscle cramping than non-susceptible individuals.

# Abstract: A077 – REPORTED SYMPTOMS OF MUSCLE PAIN AND WEAKNESS FOLLOWING MALIGNANT HYPERTHERMIA REACTIONS

**Published:** 2011-07

**Authors:** JK Kozack, DL MacIntyre

**Affiliations:** University of British Columbia, Vancouver

**Purpose/Objectives & Rationale:** To investigate reported symptoms, location, and duration of muscle pain and muscle weakness following an anaesthetic-induced malignant hyperthermia reaction.

**Relevance to Physiotherapy Practice:** Physiotherapists typically assess and treat post-operative patients experiencing muscle pain and/or muscle weakness. Knowledge of the expected presentation of these symptoms in patients affected with malignant hyperthermia is essential to effective practice.

**Materials and Methods:** Questionnaires were mailed to 106 individuals in the North American Malignant Hyperthermia Registry who were confirmed susceptible to malignant hyperthermia on both the caffeine and halothane components of the Caffeine Halothane Contracture Test between 1995 and 1999. A response rate of 58 percent was achieved, with 21 percent reporting a reaction to an anaesthetic. The questionnaire included questions and body diagrams on the presence, location, and duration of muscle pain and muscle weakness following a reaction.

**Analysis:** Percentages and overlays of body diagrams were used to analyze the data.

**Results:** Muscle pain was reported in 77 percent of the respondents, and muscle weakness was reported in 62 percent of the respondents following a malignant hyperthermia reaction. The median duration of both muscle pain and weakness was 14 days, with the most common reported sites being the calves and thighs. Fifteen percent reported muscle pain and weakness that persisted indefinitely.

**Conclusions:** Physiotherapists can expect patients recovering from a malignant hyperthermia reaction to experience muscle pain and muscle weakness, most often in the calves and thighs for a period of approximately two weeks. In some individuals, the muscle pain and weakness may not resolve.

# Abstract: A079 – ARE DXA-BASED MEASURES OF VERTEBRAL HEIGHT RELIABLE?

**Published:** 2011-07

**Authors:** AM Bonnyman, CE Webber, PW Stratford, NJ MacIntyre

**Affiliations:** * School of Rehabilitation Science, McMaster University; † Department of Nuclear Medicine, Hamilton Health Sciences, Hamilton, ON

**Purpose/Objectives & Rationale:** Bone geometry adapts to mechanical loading, however, outcome measures for assessing vertebral body geometry are limited. Although noninvasive measures of vertebral height can be obtained using dual-energy x-ray absorptiometry (DXA), estimates of reliability of these measures are lacking. The purpose of this study was to estimate the intra-rater reliability of vertebral height measures in postmenopausal women with osteoporosis.

**Relevance to Physiotherapy Practice:** Vertebral height measures may provide insight into adaptations of the spine to altered mechanical loading whether increased due to exercise or reduced due to disability.

**Materials and Methods:** Images were acquired for 32 postmenopausal women with osteoporosis (mean (SD) age: 71 (7) y). Images were analyzed in duplicate (4 weeks apart) by a novice rater using a standardized protocol and the manufacturer's software to position markers identifying the anterior, middle and posterior points on the superior and inferior endplates of each visible vertebra between T4 and L4.

**Analysis:** Relative and absolute reliability was assessed using the Intraclass Correlation Coefficient type 2,1 (ICC) and the standard error of measurement (SEM), respectively.

**Results:** Heights could be measured for T11 to L4 vertebrae in all 32 women. ICCs for average measures varied from 0.87 to 0.98 for the posterior aspect of L2 and the anterior aspect of L1. SEM varied from 0.47 to 1.17mm for the middle aspect of T12 and anterior aspect of L4, respectively.

**Conclusions:** Duplicate analyses of vertebral height by a single rater provide reliable measures of T11 to L4 in postmenopausal women with osteoporosis. These measures may be useful in examining adaptations of the spine to altered mechanical loading.

# Abstract: A082 – Decline in Function and Physiological Measures in Individuals with Hip Osteoarthritis Awaiting Joint Replacement

**Published:** 2011-07

**Authors:** K Coriolano-Da Silva, A Aiken

**Affiliations:** Queen's University, School of Rehabilitation Therapy, Kingston, ON

**Purpose/Objectives & Rationale:** The purpose of this study was to investigate the negative impact of waiting times for surgery, on individuals with hip osteoarthritis (OA), who were in the continuum of care for hip replacement, using objective measures to assess their function and physiological status.

**Relevance to Physiotherapy Practice:** Physiotherapists, who work in the orthopedic sector, diagnosing patients with hip OA, need to be informed about the impact of waiting times for hip replacement on the elderly population.

**Materials and Methods:** Subjects were selected based on their position in the continuum of care. These groups were: referral (REF) (*n =* 7), consultation (CON) (*n =* 7) and preoperatively (PREOP) (*n =* 7). Individuals were evaluated using functional (6MWT and TUG) and physiological (hip muscle isokinetic strength - Biodex and VO2peak) outcome measures.

**Analysis:** A one-way ANOVA compared differences between groups' characteristics, and then a Multivariate Analyses of Variance (MANOVA) investigated differences in functional and physiologic measures.

**Results:** The 6 minute walk test (6MWT), timed up and go (TUG) and VO2peak were significantly different between the three groups (*p* < 0.001, *p* = 0.005 and *p* = 0.001). No significant difference was observed in hip flexion and extension muscle strength between groups.

**Conclusions:** This study brought a new insight into understanding the differences in functional and physiological outcome measures in individuals with hip OA and also its impact may advance physiotherapist's knowledge, improve delivery of orthopedic services and potentially decrease surgical waiting lists.

# Abstract: A084 – THE RELATIONSHIP BETWEEN KNEE PAIN AND BODY WEIGHT IN EARLY-ONSET KNEE OSTEOARTHRITIS

**Published:** 2011-07

**Authors:** J Takacs, J Leiter, J Peeler

**Affiliations:** * Department of Human Anatomy & Cell Science; † Department of Surgery, Faculty of Medicine, University of Manitoba; ‡ Pan Am Clinic, Winnipeg, MB

**Purpose/Objectives & Rationale:** Funding was provided by the Canadian Institutes of Health Research and the Dr. Paul H.T. Thorlakson Foundation Fund.

To examine the effect of “anti-gravity” exercise on knee pain and joint forces in an overweight early-onset knee osteoarthritis population.

**Relevance to Physiotherapy Practice:** Body weight is the number one modifiable risk factor associated with the onset and progression of knee osteoarthritis. However, the ideal amount of weight loss needed to reduce knee pain and optimize joint function is unknown. The introduction of a new anti-gravity treadmill now enables our team to study the relationship between “weight loss” and knee pain/function with instantaneous feedback.

**Materials and Methods:** Twenty patients (BMI 33.6 kg/m^2^) with early-onset knee osteoarthritis walked for a period of 25-minutes at a speed of 1.4 m/s on the G-trainer (Alter-G Inc., Menlo Park CA). Subjects completed two randomized walking sessions (full weight-bearing and un-weighted). Measures included knee pain (Visual Analog Scale) and knee joint forces (tri-axial accelerometry).

**Analysis:** Paired *t-*tests and one- and two- way analyses of variance were used to detect differences.

**Results:** Mean pain during full weight-bearing and un-weighted walking was 30.2 mm and 20.9 mm, respectively. A mean un-weighting of 13.3% provided significant and immediate reduction in pain (*p* < 0.05). Initial results indicate significant differences in knee joint forces between affected and unaffected knees.

**Conclusions:** A mean un-weighting of 13.3% of body weight was sufficient to decrease subjects' pain, and this is a realistic level of weight loss for overweight patients. Subjective feedback indicates that the G-trainer is a viable rehabilitation device. Future research should be directed at establishing the limitations of this technology in the management of knee osteoarthritis and other musculoskeletal disorders.

# Abstract: A088 – A FOCUSED ETHNOGRAPHIC STUDY: UNDERSTANDING THE PERSPECTIVES AND EXPERIENCES OF RURAL COMMUNITY-DWELLING OLDER ADULTS RELATED TO EXERCISE SELF-CARE

**Published:** 2011-07

**Authors:** LJ Graham, D Connelly, D Rudman, CA Sheilds, R Stadnyk

**Affiliations:** * School of Physical Therapy, University of Western Ontario; † School of Occupational Therapy, University of Western Ontario, London, ON; ‡ School of Recreation Management and Kinesiology, Acadia University, Wolfville, NS; § School of Physiotherapy, Dalhousie University, NS

**Purpose/Objectives & Rationale:** To connect the context and culture of rural [Province Name] to older adults' values, beliefs and behaviours related to exercise self-care

**Relevance to Physiotherapy Practice:** Encouraging behaviour change in older adults who statistically are not sufficiently active to reap health benefits may relate to poor adherence or response to physiotherapy treatment given the widespread use of this modality in physiotherapy.

**Materials and Methods:** A focused ethnographic study, involving semi-structured, audio-recorded interviews and participant observation, was conducted with 20 elderly individuals, ages 65 and older. Each participant engaged in two interviews: the first focused on the shared values, beliefs, and behaviours related to exercise self-care among elderly residents of this rural community and the second (follow-up) interview which served as an additional method of data analysis to ensure the sense of co-construction resonated with the participants.

**Analysis:** Information from the initial audio-recorded interviews was transcribed, coded and constructed into themes related to values, beliefs and behaviours concerning exercise self-care. Demographic information was grouped, averaged, and identifiers removed. This information was taken into consideration for the final analysis which included repeated reading and coding of transcripts, as well as dialogue with the principle and co-investigators as a means to group analysis.

**Results:** Data from the interviews demonstrates similarities in perceptions about types of exercise as a means to self-care given passed shared experiences and values. Elements of exercise regimens and modes are discussed.

**Conclusions:** Taking the time to understand exercise self-care as your elderly patient understands it may help to inform the design and implementation of home exercise programs.

# Abstract: A090 – WEEKEND PHYSIOTHERAPY PRACTICE IN COMMUNITY HOSPITALS IN CANADA

**Published:** 2011-07

**Authors:** CA Ottensmeyer, S Chattha, S Jayawardena, K McBoyle, C Wrong, C Ellerton, S Mathur, D Brooks

**Affiliations:** Department of Physical Therapy, University of Toronto, Toronto

**Purpose/Objectives & Rationale:** The objective was the analysis of weekend physiotherapy services in acute care community hospitals across Canada.

**Relevance to Physiotherapy Practice:** Physiotherapy is an essential component of healthcare delivery in many settings. However, no national data exist on the nature of and access to weekend physiotherapy services in acute care community hospitals.

**Materials and Methods:** Questionnaires were mailed to eligible community hospitals (> 100 inpatient beds, excluding psychiatric, paediatric, rehabilitation, tertiary and long term care facilities) across Canada from January to April, 2010. Information was requested on patient referral criteria, staffing, workload, and compensation for weekend physiotherapy services, and the availability of other allied health professionals.

**Analysis:** Data were analyzed using the statistical program SPSS. Comparisons between Saturdays, Sundays and holidays included Kruskal-Wallis tests; characteristics of hospitals that provided weekend physiotherapy or did not were examined by logistic regression.

**Results:** Of 146 community hospitals deemed eligible, 71% responded. Nationally, weekend physiotherapy was offered at 69%; by region, ≥75% offered weekend care, except in Quebec (30%). Hospitals with high proportions of acute care beds were more likely to offer weekend physiotherapy services (*p <* 0.025). Services differed between Saturdays, Sundays and holidays, both in number of physiotherapists and hours worked (Kruskal-Wallis, *p* < 0.02, for each); physiotherapists were predominantly compensated via time-in-lieu. Social work services were offered on the weekend at 24% of hospitals, occupational therapy at 16%.

**Conclusions:** Substantial variation exists in regional access to weekend physiotherapy services in acute care community hospitals. To address the importance of this variation, research is required on the efficacy and cost-effectiveness of such services.

# Abstract: S001 – PHYSICAL LITERACY AND PARTICIPATION IN CHILDREN

**Published:** 2011-07

**Authors:** T Kozera, D Kriellaars

**Affiliations:** Department of Physical Therapy, School of Medical Rehabilitation, Faculty of Medicine, University of Manitoba, Winnipeg, MB

**Purpose/Objectives & Rationale:** Physical literacy (PL) is the ability to move confidently, skilfully and knowledgeably within an activity setting. Being physically literate is believed be an important component of a physically active lifestyle and programs are being developed to foster it in children. It is unknown how physical literacy, relates to participation in leisure time physical activity or performance in children. Objectives: to 1) determine the characteristics of physical literacy of children in grade 3 and 4 by sex, grade and BMI, 2) determine the relationship between physical literacy and self reported participation in leisure activity and running performance.

**Relevance to Physiotherapy Practice:** Elucidation of the relationship between physical literacy, activity and performance is critical for program development and treatment planning.

**Materials and Methods:** Cross sectional assessment of physical literacy using Physical Literacy Assessment of Youth (PLAY) developed in our lab collected from 200 youth in grade 3/4. Leisure time participation obtained through a participation checklist. Run performance (10m sprint time) was assessed.

**Analysis:** Statistical analysis using Systat (Systat Software, Inc, v13). Differences between bivariate means were evaluated by independent *t-*tests and multivariate means using ANOVA. Post hoc univariate tests were conducted to identify specific between group differences. Stepwise regression assessing contribution of sex, grade, BMI.

**Results:** Significant differences between grade (*p <* 0.01) Running performance (10m timed sprint) significantly correlated (*n =* 98, *r* = 0.44, *p <* 0.01) with physical literacy. PL was significantly correlated (*r *= 0.37, *p <* 0.01) with the number of activities a child partakes in.

**Conclusions:** This study is the first to show the association between physical literacy and participation.

# Abstract: S002 – USING PHOTOVOICE TO UNDERSTAND THE IMPACT OF A HEALTH-PROMOTING SCHOOL INTERVENTION IN A MÉTIS COMMUNITY

**Published:** 2011-07

**Authors:** SN Oosman, KE Chad, J Smylie

**Affiliations:** * University of Saskatchewan, Saskatoon; † University of Toronto

**Purpose/Objectives & Rationale:** To understand participating student perspectives on the impact of a health-promoting school program on their physical activity and nutrition knowledge, attitudes and behaviours.

**Relevance to Physiotherapy Practice:** Physical therapists can play a pivotal role in promoting healthy lifestyle choices at an individual and population health level. Implementing physical activity and nutrition programs within school settings can positively impact lifestyle choices of children and their families thereby preventing the development of chronic conditions. Implementing such interventions builds more evidence and capacity for the physical therapist role in primary health care.

**Materials and Methods:** A participatory action research framework was used to design this health promoting intervention. Sixteen Grade 3-4 students living in northern Saskatchewan participated in in-class and take-home lessons and activities promoting physical activity and healthy eating. Interviews and photovoice, a qualitative research method that uses photography, were used to collect information before and after the intervention.

**Analysis:** Photographs and transcripts of the interviews were qualitatively analyzed using the Atlas-ti computer software program which stores, codes, retrieves and analyzes text, photographs and audio files.

**Results:** Student awareness, knowledge and attitudes towards healthy lifestyle choices improved over the duration of the intervention. Students were able to speak specifically about what changes they have made in their lives to promote their health and prevent such conditions as type 2 diabetes.

**Conclusions:** This project clarifies the role a health-promoting school has on influencing physical activity and nutrition knowledge, attitudes and behaviours of elementary school children. Furthermore, physical therapists are well situated to conduct such community-based health interventions thereby playing a significant role in health promotion and long-term prevention of chronic conditions.

# Abstract: S004 – FACTORS AFFECTING TOE CLEARANCE POST-TOTAL HIP ARTHROPLASTY: A PILOT STUDY

**Published:** 2011-07

**Authors:** T Burgess, C Armstrong, S Fleming, K Foley, A Singh, S Robarts, D Kennedy, K Zabjek

**Affiliations:** * University of Toronto, Physical Therapy Department; † Sunnybrook Holland Orthopaedic and Arthritic Centre, Rehabilitation, Toronto

**Purpose/Objectives & Rationale:** To examine operative hip abductor strength, range of motion and non-operative toe clearance and the associations between these variables in subjects three to fifteen months post total hip arthroplasty.

**Relevance to Physiotherapy Practice:** Long-term physical impairments can persist after total hip arthroplasty, however there is a lack of knowledge regarding whether these impairments predispose individuals to increased risk of falls.

**Materials and Methods:** Twelve consecutive, eligible participants (mean age 69.4, standard deviation 5.4) post- primary total hip arthroplasty were recruited and randomized in order of evaluated operative hip range of motion, hip abductor and extensor strength, and toe clearance when walking. Strength was assessed using hand held dynamometry, range of motion with goniometry, and toe clearance with motion capture. Medical charts were reviewed retrospectively to abstract sample characteristics.

**Analysis:** Using statistical software, cross-sectional quantitative analysis was performed using non-parametric Spearman's rho correlation tests (The *p*-value was set at 0.05).

**Results:** Range of motion and strength had a weak and non-significant correlation with toe clearance (*p > *0.05). Two outliers were subsequently identified and removed with hip range of motion continuing to have a weak correlation to contralateral toe clearance. The correlation between peak hip abductor and extensor strength was strengthened and displayed a trend towards a negative correlation.

**Conclusions:** There is a trend towards a negative correlation between operative hip abductor and extensor strength and contralateral toe clearance post total hip arthroplasty. Further investigation with a larger sample size is warranted.

# Abstract: S005 – GLUTEAL MUSCLE STRENGTH AND ACTIVATION IN A CHRONIC LOW BACK PAIN GROUP

**Published:** 2011-07

**Authors:** TL Penney, M Ploughman, M Austin, JM Byrne, DG Behm

**Affiliations:** * Eastern Health /Memorial University; † Eastern Health; ‡ Memorial Univeristy; St. Clare's Physiotherapy, Eastern Health, St. John's, NL

**Purpose/Objectives & Rationale:** To compare the strength and timing of the gluteal muscles in a chronic low back pain group compared to controls.

**Relevance to Physiotherapy Practice:** Asymmetry of hip range of motion and trunk muscle strength and timing deficits are associated with chronic low back pain. However, it is unknown if gluteal strength and motor control deficits are related to chronic low back pain.

**Materials and Methods:** We recruited 22 male and female subjects with chronic low back pain and 21 age and gender matched healthy subjects. Maximum bilateral hip abductor and extensor strength was measured using a handheld dynamometer. We recorded the onset of the gluteus medius and maximus contraction with electromyography of the stance side during single leg stance.

**Analysis:** A repeated measure analysis of the variance was performed to compare strength and timing of four hip muscles during maximal muscle testing and single leg stance.

**Results:** The mean hip strength of each gluteal muscle in the low back pain group was lower than the control group, but only significant in the left gluteus maximus (*F* = 1.94, *p <* 0.05). A post-hoc comparison test showed no significant group difference for the onset of the gluteal muscle activation (*p >* 0.05).

**Conclusions:** Asymmetry of gluteal muscle strength is demonstrated in a chronic low back pain group while motor control deficits are not identified. These findings support the inclusion of gluteal muscle strength assessment in the chronic low back pain patient.

# Abstract: S006 – ARE PRESEASON REPORTS OF NECK PAIN, DIZZINESS AND HEADACHE RISK FACTORS FOR CONCUSSION IN MALE YOUTH ICE HOCKEY PLAYERS?

**Published:** 2011-07

**Authors:** K Schneider, C Emery, J Kang, G Schneider, W Meeuwisse

**Affiliations:** * University of Calgary, Faculty of Kinesiology; † University of Calgary, Faculty of Medicine

**Purpose/Objectives & Rationale:** The purpose of this study was to determine the risk of concussion in youth male hockey players associated with reports of neck pain, headaches and dizziness.

**Relevance to Physiotherapy Practice:** Concussion is a commonly encountered injury by physiotherapists. Identification of risk factors will contribute to the prevention of concussion.

**Materials and Methods:** This was a secondary data analysis from two cohort studies examining the risk of concussion associated with body checking among ice hockey players (Pee Wee (11-12 years) and Bantam (13-14 years). Each player completed a pre-season demographic, injury history questionnaire and Sport Concussion Assessment Tool (SCAT). Concussions were recorded during the season using a validated injury surveillance system.

**Analysis:** Incidence rate ratios (IRR) were estimated using Poisson regression, adjusting for cluster by team, exposure hours and covariates.

**Results:** A total of 3832 males (280 teams) participated. Preseason reports of neck pain and headache were risk factors for concussion (IRR=1.68 (95% CI, 1.16-2.44 and 1.47 (95% CI, 1.02-2.13 respectively)). Dizziness was a risk factor in the Pee Wee non-body checking cohort (IRR=3.11 (95% CI, 1.33, 7.25)). A combination of any two symptoms was a risk factor in the Pee Wee non-body checking cohort (IRR = 3.65 (95% CI, 1.2, 11.0)) and the Bantam cohort (IRR = 2.38 (95% CI, 1.12, 5.07)).

**Conclusions:** Male youth athletes reporting headache, dizziness and/or neck pain at baseline were at an increased risk of concussion. Baseline testing may aid in identifying individuals at a higher risk for concussion who might benefit from treatment or other preventive measures.

# Abstract: A035 – VIRTUAL REALITY SYSTEMS IN PEDIATRIC REHABILITATION: MAKING SENSE OF THE MANY OPTIONS

**Published:** 2011-07

**Authors:** D Levac, J Galvin

**Affiliations:** * School of Rehabilitation Science, Institute of Applied Health Sciences, McMaster University, Hamilton, ON; † Murdoch Children's Research Institute, Victorian Paediatric Rehabilitation Service, The Royal Children's Hospital, Department of Paediatrics, University of Melbourne, Australia

**Purpose/Objectives & Rationale:** Although multiple virtual reality (VR) systems are under evaluation within pediatric motor rehabilitation, little information supports decisions related to addressing individual therapeutic goals. This work describes the specific qualities of VR systems reported in the paediatric motor rehabilitation literature and proposes a classification framework to assist therapists in making informed clinical decisions.

**Relevance to Physiotherapy Practice:** VR is increasingly being integrated into clinical practice, yet clinicians are challenged to understand the similarities and differences between VR systems when making choices related to system purchase or use.

**Materials and Methods:** A comprehensive database search was undertaken to explore VR systems used in motor rehabilitation for children. Description of these systems, motor learning literature and expert opinion informed development of a classification framework.

**Analysis:** Reflection and review of theoretical, practical and logistical factors relevant to clinical decision-making in pediatric motor rehabilitation informed the descriptive analysis and development of categories that enabled differentiation between VR systems.

**Results:** The descriptive analysis details six VR systems according to 12 user, system and context variables. The systems included in the framework require the user to move their body through three-dimensional space, provide multi-sensory augmented information, and offer the potential for consistent task repetition under identical conditions, with subsequent categorization by specific movement, measurement and challenge options.

**Conclusions:** This study organizes available information to facilitate clinical understanding and decision-making about VR systems. Additional research directions are identified to further support the use of VR in clinical practice.

# Abstract: A045 – EMPLOYMENT PREFERENCES AMONG INTERNATIONALLY EDUCATED PHYSICAL THERAPISTS (IEPTS) IN ONTARIO, CANADA

**Published:** 2011-07

**Authors:** MD Landry, S Baptist, M Verrier, A Baumann, S Mandoda, E Stokes

**Affiliations:** * Department of Physical Therapy, University of Toronto, Toronto; † Faculty of Health Science, McMaster University, Hamilton, ON; ‡ Trinity College Dublin, Dublin, Ireland

**Purpose/Objectives & Rationale:** The demand for physical therapy services is growing, and there is an increasing proportion of IEPTs entering the workforce. Some evidence suggests that IEPTs from low-income countries are over represented in specific sectors. The purpose of this study was to explore employment perceptions and preferences among IEPTs.

**Relevance to Physiotherapy Practice:** Understanding perception of workplace preferences provides valuable data for human resource planning.

**Materials and Methods:** A qualitative research methodology was employed using telephone semi-structured key informant interview with IEPTs (*n =* 11). Informants were recruited from a cohort enrolled in a university-based bridging program.

**Analysis:** Interviews were recorded and transcribed verbatim. Qualitative data software was used to code transcriptions for themes.

**Results:** The majority of the participants were trained in low-income countries; most were currently employed as rehabilitation assistants. The major themes included: alignment between future employment preference and expectation, credentialing experiences, and employment barriers and facilitators. Overall, participants were optimistic about employment opportunities, and perceived the ethnic diversity of urban centers of the province as an opportunity. Many described challenges navigating the credentialing process. The majority reported that their workplace preference is a hospital, but there was a strong perception that hospitals were more likely to employ Canadian trained physical therapists.

**Conclusions:** IEPTs from low-income countries describe important challenges during the credentialing and pre-employment phases. Our analyses signal a need to further consider the degree to which these data have implications for recruitment and retention within a complex and evolving rehabilitation workforce.

# Abstract: A046 – IMPACT OF CULTURAL CHANGE ON CUSTOMER CARE: A CRITICAL ETHNOGRAPHY

**Published:** 2011-07

**Authors:** R Brander, M Paterson, Y Chan, M Ruffolo

**Affiliations:** * Providence Care and Queen's University; † Queen's University, School of Rehabilitation Therapy; ‡ Queen's School of Business and The Monieson Centre; § Providence Care, Kingston, ON

**Purpose/Objectives & Rationale:** To explore organizational culture and to impact positive change in customer service with interprofessional health providers and leaders.

**Relevance to Physiotherapy Practice:** Physiotherapists maintain essential competencies that position them as leaders in customer service and partnership in the provision of care which enhance health outcomes.

**Materials and Methods:** Phase 1: Semi-structured focus groups explored concepts related to customer service prior to, immediately after, and 6-months after the completion of patient and family centred care education. Phase 2: Middle mangers completed surveys exploring their views on customer service. Results from phase 1 were presented in a semi-structured focus group to identify cultural change ideas. Phase 3: Change ideas were discussed with senior leaders as key informants. Strategies for directed change were identified.

**Analysis:** A domain analysis of focus group transcripts led to the description of organizational cultural categories and significant cultural themes. Field observations and confirming analysis with experts enhanced rigour. Demographic and survey data were tabulated. Open-ended survey responses were analyzed for additional cultural themes.

**Results:** The value attributed to client relationships demonstrated positive change after the educational intervention and was sustained for 6 months. Identification of cultural categories led to description of major cultural themes. Themes were used to generate change ideas to impact quality improvement in customer service.

**Conclusions:** Customer service holds different meanings for different health providers. Focussed education can lead to positive changes in views. Ethnographic analysis enabled the generation of concrete change ideas to positively impact partnerships. The impact of these will be further examined on the culture of partnership within the organization.

# Abstract: A053 – RURAL REHABILITATION PRACTICE

**Published:** 2011-07

**Authors:** RK Roots, LC Li, L Bainbridge, H Brown

**Affiliations:** University of British Columbia, Vancouver

**Purpose/Objectives & Rationale:** The objective of this research was to construct an understanding of rural rehabilitation practice from the perspectives of physiotherapists (PTs) and occupational therapists (OTs) in rural British Columbia.

**Relevance to Physiotherapy Practice:** A better understanding of rural rehabilitation practice will help refine the entry-level physiotherapy curriculum and inform recruitment/retention strategies for rural areas.

**Materials and Methods:** A purposive sample of PTs and OTs was recruited by mail to rural workplaces. Participants were selected to ensure a broad variety of work experiences, roles and practice settings. Interview questions asked participants to describe their practice, the skills and knowledge perceived as unique to rural, challenges faced, and mitigating strategies.

**Analysis:** Guided by Interpretive Description, transcripts were analysed inductively to extrapolate practice concepts. The researcher's interpretations considered the implications for policy, practice and education, and were presented to participants for comment.

**Results:** Thirteen PTs and six OTs were interviewed. The central theme that emerged was the influence of the rural context, such as geographical distance and limited health human resources/health-related services, on the definition of health and professional practice. Participants described stretching their roles and specializing as generalists to overcome resource shortages and meet client needs. Skills in reflective practice, networking and collaboration were deemed essential to maintaining proficiency. Clinical placements in rural areas, mentoring and improving access to continuing education were regarded as critical to recruitment and retention.

**Conclusions:** Understanding the influence of the rural context on rehabilitation practice is fundamental to preparing students to develop a career in rural practice and to supporting PTs working in rural areas.

# Abstract: A054 – INUIT COMMUNITY THERAPY ASSISTANTS: A UNIQUE EDUCATION PROGRAM AND INNOVATIVE SERVICE DELIVERY MODEL FOR A REMOTE PRACTICE CONTEXT IN NUNAVUT

**Published:** 2011-07

**Authors:** T Miller Mifflin, C McNeil, D Driedger, M Fricke, M Acthemichuk, M Bzdell, J Robison

**Affiliations:** * Government of Nunavut, Department of Health and Social Services, Iqaluit, NU; † University of Manitoba, Winnipeg, MB

**Purpose/Objectives & Rationale:** To increase the quantity and cultural relevance of rehabilitation services across Nunavut by training and employing Inuit as multidisciplinary assistants in remote communities, and implementing an innovative service delivery model using remote supervision.

**Relevance to Physiotherapy Practice:** Physiotherapists working in remote, under-serviced areas with aboriginal populations require novel service delivery methods to meet client needs.

**Materials and Methods:** A feasibility study determined the need for Inuit rehabilitation assistants and recommended a service delivery model involving assistants working remotely from supervisors. Curriculum consultants developed the Community Therapy Assistant diploma program for assisting physiotherapists, occupational therapists, speech-language pathologists and audiologists. The program was delivered by Arctic College in Iqaluit. The practice role and supervision guidelines, including communication via Telehealth, were revised throughout the first year of employment.

**Analysis:** The numbers of students enrolled in and graduating from the Community Therapy Assistant program were observed, as were the number of graduates employed in rehabilitation one year later. Interviews with assistants and supervisors determined the success of project objectives and identified areas for further training.

**Results:** Seven of eight enrolled students completed the diploma program. One year after graduation, four were employed as rehabilitation assistants. Community Therapy Assistants were successful in delivering rehabilitation services to Inuit clients in their own communities and language. Further training was identified for specific clinical skills and advanced Inuktitut/English interpreting.

**Conclusions:** Training and employing community members as multidisciplinary assistants is an effective way to increase the quantity and cultural relevance of physiotherapy services in remote, cross-cultural contexts.

# Abstract: A060 – DIRECT ACCESS AND PATIENT SELF-REFERRAL – DEVELOPING A GLOBAL MAP

**Published:** 2011-07

**Authors:** E Stokes, T Bury

**Affiliations:** * Department of Physiotherapy, Trinity College, Dublin, Ireland; † World Confederation for Physical Therapy, London, UK

**Purpose/Objectives & Rationale:** In October 2009 the Canadian Physiotherapy Association co-hosted, an international summit on Direct Access (DA) in Physical Therapy. One action from this summit was a global mapping exercise; this study reports on the results.

**Relevance to Physiotherapy Practice:** Understanding the extent, nature, barriers and facilitators of DA to physical therapy within the health services of the member organisations (MOs) of the World Confederation for Physical Therapy may inform advocacy for and by physical therapists, and users of their services, as well as facilitate policy development.

**Materials and Methods:** The initial iteration of the survey was piloted at the European Region of WCPT in May 2010 to seek a common understanding of concepts regarding DA. The second iteration was evaluated by an international reference group. The final online survey was circulated to all MOs in August 2010.

**Analysis:** Descriptive statistics were used to analyse the data.

**Results:** The response rate was 60.3% (*n =* 61). 82% of MOs reported having national legislation that regulates practice. In 47% of MOs, this legislation defines scope of practice and in 50% of MOs it allows users of services to self-refer to physical therapy. 30% of MOs reported, even with DA, private insurance providers will not provide reimbursement. The top 4 barriers to advancing DA were: reimbursement models, views of politicians/medical colleagues and legislation.

**Conclusions:** There are many similarities across MO's. This first global map of DA may be used to inform national and international strategy and policy.

# Abstract: A061 – ESSENTIAL COMPETENCIES FOR INTRAPROFESSIONAL PRACTICE IN PHYSICAL THERAPY

**Published:** 2011-07

**Authors:** W Jelley, N Larocque

**Affiliations:** * University of Ottawa; † La Cité collégiale, Ottawa

**Purpose/Objectives & Rationale:** The purpose of this research project was to determine the essential competencies involved in intraprofessional collaborative practice in physical therapy.

**Relevance to Physiotherapy Practice:** Although there is increased demand to successfully incorporate support personnel into clients' care plans, the essential competencies necessary for effective intraprofessional collaboration are not well established. In fact there is very little literature regarding competencies related to effective integration of support personnel in any health care profession.

Although there is increased demand to successfully incorporate support personnel into clients' care plans, and the essential competencies necessary for effective intraprofessional collaboration are now well established, in fact there is very little literature regarding competencies related to effective integration of support personnel in any health care profession

**Materials and Methods:** An electronic survey using a 5-point scale was developed in English and French. The survey was then distributed by email to all registered physical therapists in the province of Ontario by the College of Physiotherapists of Ontario. Physiotherapists were encouraged to share the survey with support personnel in order to receive their feedback as well. Over 1100 physiotherapists and support personnel completed and returned the survey. All replies were anonymous.

**Analysis:** Each item in the survey was scored and the results compared based on demographoc information. Text responses were coded using NVivo 8.

**Results:** Analysis of the data identified 5 competency elements perceived by the respondants as essential to effective intraprofessional practice. The 5 elements identified were active listening, expressing ideas concisely and respectfully, recognizing and communicating all significant changes in client's status, completing actions based on one's own role constraints, and building trust and respecting others knowledge and skills. Sorting the data on area of practice or type of practice i.e. public or private did not change the outcome.

**Conclusions:** Consensus concerning the essential elements of intraprofessional practice was clear in the 1100 responses received from the Ontario based survey of physiotherapists and support personnel.

# Abstract: A063 – THE DEVELOPMENT OF STANDARDS FOR AN OTA & PTA EDUCATION ACCREDITATION PROGRAM

**Published:** 2011-07

**Authors:** D Burnett, C Beggs, C DesLauriers

**Affiliations:** * OTA & PTA Education Accreditation Program; † Physiotherapy Education Accreditation Canada; ‡ Canadian Association of Occupational Therapists

**Purpose/Objectives & Rationale:** The need for a national accreditation program for occupational therapist assistant (OTA) and physiotherapist assistant (PTA) education has been identified to promote ongoing educational quality. The purpose of this project was to develop accreditation standards for PTA and OTA education that are relevant and responsive and will serve as the framework for a process of continuous quality assessment and improvement.

**Relevance to Physiotherapy Practice:** A national accreditation program will ensure minimum standards of education in Canada, promote national portability of credentials for OTAs and PTAs, and support the increased access of Canadians to rehabilitation services.

**Materials and Methods:** Draft 1 of the standards was developed based on the accepted 5 + 1 standards model, and with reference to competency standards for OTAs and PTAs, and accreditation standards for occupational therapist and physiotherapist education. Standards were subjected to stakeholder consultations including fourteen country-wide focus groups; an online survey generating over 2000 responses, and consultations with a Standards Development Working Group and Advisory Committee.

**Analysis:** Three iterations of the standards were developed according to themes identified at each phase of the consultation process.

**Results:** A robust and relevant set of accreditation standards has been developed to serve as the framework for evaluation of OTA and PTA education programs. The standards will be piloted in three sites across the country in 2011.

**Conclusions:** A complex, multi phase process with wide stakeholder input ensures that the accreditation standards successfully serve as the framework through which quality in OTA and PTA education can be assessed.

# Abstract: A064 – INTEGRATING PHYSIOTHERAPISTS WITHIN ONTARIO PRIMARY HEALTH CARE TEAMS: PERSPECTIVES OF FAMILY PHYSICIANS AND NURSE PRACTITIONERS

**Published:** 2011-07

**Authors:** SP Dufour, SD Lucy

**Affiliations:** * Graduate Program in Health & Rehabilitation Sciences; † School of Physical Therapy, Faculty of Health Sciences, The University of Western Ontario, London, ON

**Purpose/Objectives & Rationale:** To explore perspectives of family physicians (FPs) and nurse practitioners (NPs) regarding the integration of physiotherapists within Ontario primary health care (PHC) teams.

**Relevance to Physiotherapy Practice:** The international literature suggests a number of benefits related to integrating physiotherapists into PHC teams, yet remain only sparsely so within Ontario. The perspectives of “core” team members (FPs and NPs) on this issue are currently unknown.

**Materials and Methods:** Following qualitative description method, FPs and NPs practicing within Ontario PHC teams were purposefully sampled. Twenty individual semi-structured in-depth interviews were conducted. Eight interviewees worked with a physiotherapist.

**Analysis:** All interviews were transcribed verbatim and inputted in to NVIVO-8 to facilitate analysis. In the first phase transcripts were reviewed and coded by three independent researchers. The second analytic stage involved generation of reports leading to higher order codes and themes. This content analysis was an iterative, interactive and reflexive process.

**Results:** Five key themes emerged: (1) lack of physiotherapists, a critical gap; (2) high perceived demand for and value of physiotherapists; (3) possible beneficial outcomes; (4) more appropriate use of health human resources (HHR) and capacity building; and (5) lack of funding a key barrier to inclusion

**Conclusions:** The mandate of PHC in Ontario articulates a shift to a comprehensive perspective of health requiring a diverse team of providers, inclusive of physiotherapists. The lack of presence physiotherapists have within Ontario PHC teams appears to be rooted at the health care system level, rather than at that of current “core” providers (FPs and NPs).

# Abstract: A065 – ENACTING PHYSIOTHERAPISTS' ROLE WITHIN ONTARIO PRIMARY HEALTH CARE TEAMS

**Published:** 2011-07

**Authors:** SP Dufour, SD Lucy

**Affiliations:** * Graduate Program in Health & Rehabilitation Sciences; † School of Physical Therapy, Faculty of Health Sciences, The University of Western Ontario, London, ON

**Purpose/Objectives & Rationale:** To develop a theoretical framework in support of understanding the enactment of physiotherapists' role within Ontario primary health care (PHC) teams.

**Relevance to Physiotherapy Practice:** The mandate of PHC teams in Canada requires a broad range of providers inclusive of physiotherapists. Yet physiotherapists are integrated into Ontario PHC teams to a limited degree and how they are delivering service is currently unknown.

**Materials and Methods:** Drawing from a pragmatic grounded theory approach, 12 physiotherapists working within community health centers (*n =* 8) and family health teams (*n =* 4) participated in 16 (11 purposefully, five theoretically sampled) semi-structured in-depth in-person interviews.

**Analysis:** Interviews were transcribed verbatim, and inputted into NVIVO-8. In the first phase of analysis, three independent researchers coded transcripts. The second iteration of analysis involved generation of higher order codes, leading to the emergence of key themes. This constant comparative analysis was an iterative, interactive process as the research team accommodated new data and insights.

**Results:** Four key themes emerged as a framework impacting the enactment of physiotherapists' role: (1) Ontario's PHC mandate and funding models; (2) community and population served; (3) interprofessional team; and (4) physiotherapist themselves. The role was varied, dynamically evolving, a balancing act between individual and group care, bridging care across different provider groups.

**Conclusions:** Physiotherapists take on a variety of roles within Ontario PHC teams. This study demonstrated that role enactment reflects a broad holistic perspective of health, impacted by the community served and the team with which the physiotherapist works.

# Abstract: A069 – WHAT TYPE OF CONTINUING EDUCATION IS PREFERRED BY THERAPISTS WORKING IN PAEDIATRICS?

**Published:** 2011-07

**Authors:** J Gordon

**Affiliations:** Provincial Paediatric Therapy Recruitment and Retention Coordinator

**Purpose/Objectives & Rationale:** The Provincial Paediatric Therapy Recruitment and Retention Coordinator (PTRRC) performed a survey of BC therapists working with children and youth with special needs to determine the nature of professional development events considered most important to the paediatric therapy community.

**Relevance to Physiotherapy Practice:** Limited fiscal resources require the PPTRRC to focus on planning events therapists feel are of the highest priority.

**Materials and Methods:** An online survey was emailed to paediatric therapists (OTs, PTs, and SLPs) across BC. Therapists were asked to rank various education opportunities in their order of preference.

**Analysis:** The PPTRRC analyzed the results of the survey to determine the continuing education preference of paediatric therapists. The literature review results were analyzed to determine if there were themes within the survey data supported by the literature.

**Results:** Face-to-face opportunities ranked as the highest priority to receive PPTRRC support, while videoconferencing and web-based opportunities were ranked as the least preferred priority. Face-to-face opportunities were ranked highest regardless of the population of the therapist's community, although continuing education bursaries/grants were equal in ranking with face-to-face opportunities as the preferred medium for continuing education amongst therapists from communities of less than 30,000.

**Conclusions:** This survey suggests that administrators and managers responsible for continuing education budgets and the development of continuing education events ensure that face-to-face learning opportunities remain available for therapists.

# Abstract: A070 – ESTABLISHMENT OF RELIABILITY IN A POINTS-BASED CASELOAD MEASURE FOR PAEDIATRIC REHABILITATION THERAPISTS

**Published:** 2011-07

**Authors:** K Davidson

**Affiliations:** Faculty of Medicine, Department of Physical Therapy, University of British Columbia, Vancouver

**Purpose/Objectives & Rationale:** There are few guidelines available to inform manageable caseload size in paediatric rehabilitation nationally or within British Columbia. Phase 2 of a three phase study, the current project investigated the intrarater reliability of a previously piloted points-based measure, and of its workload manageability scale.

**Relevance to Physiotherapy Practice:** Workload is an issue in all contexts of Physiotherapy and rehabilitation; this points-based caseload measure was developed in order to provide an alternative to the traditional caseload numbers approach to caseload measurement. The broader goal of the three phase study is to establish statistically supported points-based guidelines for paediatric therapists.

**Materials and Methods:** Ethics approval was received from the Behavioural Research Ethics Board at the University of British Columbia. Participants (Paediatric OTs, PTs and SLPs) completed the online questionnaire twice in two weeks, using instructions to assign points to each client on their caseload and to rate the manageability of their workload.

**Analysis:** Data was analyzed with support from the Clinical Research Support Unit at the Child and Family Research Institute (CFRI). We determined test-retest reliability using Pearson correlation coefficients.

**Results:** Correlations between Total Scores A&B were significant at the *p <* .01 level. Correlations between Manageability A&B were significant at the *p <* .01 level for the Total and for the PT group. Correlations between each component score A&B were significant at the *p <* .01 level. A consistently positive correlation between component scores and total score was observed. All significant correlation values were in the excellent or adequate range.

**Conclusions:** This measure can be confidently used in further research.

# Abstract: P001 – CASELOAD MANAGEMENT TOOL — A STRUCTURED APPROACH TO DETERMINING EFFECTIVE WORKLOADS IN PHYSIOTHERAPY, OCCUPATIONAL THERAPY AND SPEECH-LANGUAGE PATHOLOGY

**Published:** 2011-07

**Authors:** D Burnett, C Miller, C DesLauriers, S Fotheringham

**Affiliations:** * Caseload Management Planning Tool Project; † Canadian Physiotherapy Association; ‡ Canadian Association of Occupational Therapists; § Canadian Association of Speech-Language Pathologists and Audiologists

**Learning Objectives and Session Content:** Upon completion of this session participants will:

1. Understand the factors and processes which lead to the development of the Caseload Management Tool (Tool).
2. Understand how to use the Tool at their workplace.
3. Discuss the potential application of the Tool to caseload management, allocation of human resources and guideline development for caseload management.

This session will begin with an overview of the background and rationale for the project followed by a brief description of the process for development of the Tool: a literature review, formation of a multi-stakeholder advisory committee, broad member consultation and a pilot of the Tool in a variety of clinical contexts and settings. Lessons learned from these steps will be integrated into the content.

The presentation will then describe the Tool's three steps which include:

1. Establishing human resources time available for comprehensive client care;
2. Determining client intervention time required; and
3. Matching client intervention times to human resources time.

Reference will be made to the educational tools which have been developed and identified to assist therapists with the Tool's application.

Open participant discussion will follow related to:

- The application of the Tool in a variety of clinical practice areas.
- The use of the Tool for individual caseload management and reflection.
- The utility of the Tool for policy makers and administrators for health human resources planning and resource allocation.
- The value of the Tool in caseload guideline development.

The session will conclude with a summary of next steps to promote widespread use of the Tool, report lessons learned and ensure the sustainability of the initiative.

**Relevance to Physiotherapy Profession:** Caseload/workload management in physiotherapy has and continues to be determined primarily by economic factors, that is, the funding allocations available for physiotherapist positions. With global budgets steadily diminishing, little consideration is given to patient and population health needs in workforce planning. The status quo, or existing numbers of funded positions, is frequently used as the gold standard despite reports of therapists facing increased volume of patients with complex health issues. This situation has resulted in patients experiencing lengthy wait times and unmet health needs and growing concerns for therapist recruitment and retention. The Tool provides an evidence-informed approach to caseload/workload management that has potential benefits for both physiotherapists and the clients they serve.

**Target Population:** Physiotherapist clinicians, administrators and policy makers working in a variety of practice areas.

**Description of Supporting Evidence:** The human resources data on physiotherapists reveal that their numbers have steadily increased over the past 20 years. Despite this growth, findings from the recent literature indicate that physiotherapists continue to face unmanageable caseloads. Excessive work intensity leads to workplace stress with subsequent sick leave and burnout, which in turn places increased pressures on remaining workers. The informed deployment of human resources must become a major priority for employers in order to minimize attrition and encourage the retention of skilled workers. Caseload management has been an ongoing issue of concern for physiotherapists, occupational therapists and speech-language pathologists. Substantial work has been carried out to date by the professional associations representing these professions which set the foundation for this project.

An evaluation framework suggested by the Human Capital Alliance (2006) describes four categories of caseload management tools including: ratio-based, procedure-based, categories of care based, and diagnostic case mix-based methodologies. Using this framework, analysis and synthesis of the literature for physiotherapy, occupational therapy and speech-language pathology as well as other professions, notably nursing, reveals ongoing efforts and multiple approaches to promote effective caseload management. Despite these diverse and innovative methodologies, there is clearly no one superior model that suits all practice contexts and service delivery systems. The evidence confirms that effective caseload management remains a highly complex and variable process in which many factors must be considered including: client classification system, flexibility, client complexity, population health perspective, simplicity, evidence-based, provider experience, and organizational factors. A hybrid approach which combined elements from several models seemed the most promising and appropriate for the purposes of this project.

**Description:** The session will consist of a lecture slide presentation on the development of the Tool and description of the Tool processes (approximately 30 minutes). This will be followed by an open discussion on issues such as application of the Tool to various clinical contexts, its use in deployment of health human resources and suggestions for promotion of its use. Any participants from the pilot will be encouraged to share their impressions and experience with the Tool, its applicability in a variety of service settings and suggestions for promoting its use.

**Conclusions:** The Tool matches human resource time available to the time requirements of clients based on the complexity of the interventions in order to meet health needs. It provides a systematic, evidence-informed approach for determining manageable and effective caseloads and the efficient allocation of available human resources within a service. The Tool will be globally useful and relevant to the profession when its applications are reported and shared. The Tool is a living document which will evolve according to changing practice, professional and service trends. Clinicians and administrators are strongly encouraged to use the Tool, report their findings and share their experiences to ensure its relevance, appropriateness and continued usefulness to advance the physiotherapy profession and contribute to the informed and effective utilization of these health professionals.

# Abstract: P002 – BACK TO THE GYM AFTER HIP AND KNEE REPLACEMENT

**Published:** 2011-07

**Authors:** MD Westby, G Gill

**Affiliations:** Mary Pack Arthritis Centre, Vancouver Coastal Health, Vancouver

**Learning Objectives and Session Content:** More than 62,000 Canadians undergo total hip or knee replacement surgery each year to relieve the pain and functional limitations associated with arthritis. A growing body of research shows that many individuals are not reaching their full potential following surgery and continue to have muscular weakness, gait abnormalities and functional difficulties more than two years later. A fitness facility or gym provides an ideal community setting to address long term physical impairments and functional limitations once post-acute rehabilitation is complete. It is important to provide evidence-based and consistent guidance on appropriate use of fitness equipment to ensure patient safety and exercise effectiveness.

**Objectives:**

By the end of this session, participants will:

1. Be familiar with the evidence on the benefits, risks and biomechanical issues associated with strength training and aerobic exercise equipment found in a typical gym setting.
2. With the use of two case studies, describe the physical impairments and functional limitations commonly seen following these surgeries that can be addressed in a gym setting.
3. Appropriately advise patients, health and fitness professionals on safe and effective use of exercise equipment following hip and knee replacement.

**Relevance to Physiotherapy Profession:** Many adults undergoing a total hip or knee replacement will choose to continue their rehabilitation process in a fitness or gym facility after discharge from the physiotherapy practice setting. While some will receive health and/or fitness professional guidance or supervision, others will exercise independently with no further professional support. The quality of the supervision will vary based on the professional's clinical and fitness knowledge, experience with hip and knee replacement clients and familiarity with the ever changing array of fitness equipment. This interactive session will give participants the opportunity to integrate their knowledge of biomechanical considerations (joint kinematics and kinetics), clinical experience and the related research evidence and apply these to common strength training and aerobic exercise equipment. Two case studies will allow for clinical problem solving and group discussion.

**Target Population:** This session will be of interest to health and exercise professionals including clinicians, program designers, educators and researchers interested in total joint replacement rehabilitation and exercise.

**Description of Supporting Evidence:** Adults undergoing total joint replacement surgery exhibit marked functional impairments and activity limitations in the period leading up to surgery and in some cases, several years after (Meier 2008, Trudelle-Jackson 2002). Cardiovascular deconditioning puts these individuals at risk for coronary artery disease (Philbin 1995) and surgical complications. Prolonged lower extremity weakness is a major risk factor for falls in older adults.

Regular, moderate intensity physical activity is recommended for older adults and has numerous health benefits including weight control, bone health and improving aerobic capacity (http://www.csep.ca). Long-standing strength deficits can be addressed through progressive strength training using appropriate forms of resistance. A growing body of biomechanical data and descriptive and pilot studies suggest that many popular types of exercise equipment are safe and effective for individuals with arthritis and joint replacement surgery (Westby 2001) and thus should be considered an option for individuals in the later phases of joint replacement rehabilitation.

**Description:** This session will be an interactive, practical format with opportunity for participant discussion and clinical reasoning.

**Conclusions:** With knowledge of the evidence and biomechanical factors associated with common types of exercise equipment found in fitness or gym settings, physiotherapists will be better equipped to provide safe and effective advice on community-based exercise and thus promote regular physical activity in the joint replacement population. A long-term exercise program beyond the relatively short post-operative therapeutic intervention will help individuals reach their full functional potential and reduce the risk of falls and other health issues after hip and knee replacement.

# Abstract: P003 – TRAINING SPEED OF MOVEMENT POST STROKE

**Published:** 2011-07

**Authors:** SJ Garland, VL Gray

**Affiliations:** * Department of Physical Therapy and Pollock CL, Rehabilitation Sciences, University of British Columbia, Vancouver; † Health and Rehabilitation Science, University of Western Ontario, London, ON

**Learning Objectives and Session Content:** This presentation will provide an overview of the principles of motor control for movement production and their application to the retraining fast movements that are necessary for balance and community reintegration following stroke. Specifically, this presentation has the following objectives: 1) to provide an overview of the motor control required to produce fast movement; 2) to discuss the importance of, and evidence for, retraining speed of movement following stroke and 3) to provide examples of exercises to retrain speed of movement after stroke.

**Relevance to Physiotherapy Profession:** Balance impairments and mobility restrictions are common after stroke; for example, there are reports that community ambulators post-stroke have fall rates of 73% and falls occur most frequently during walking. Considerable attention has been paid, therapeutically and in research, to some of the motor impairments following stroke including spasticity, muscle weakness and poor coordination of muscle activation. Less is known about impairments in the speed of movement or the ability to produce power. However, speed or power is imperative to the ability to take an effective step to regain balance.

**Target Population:** This session will be of interest to physiotherapists and researchers interested in mobility and neurological rehabilitation.

**Description of Supporting Evidence:** Motor control impairments following stroke result in force production that is slow, weak, and lacking in precision. Remodelling of the hemiparetic muscle leads to slower contractile properties and weakness. Impaired coordination of muscle activity reduces the efficiency of force production in movements and functional tasks. In terms of postural control, these impairments make it difficult to produce force with sufficient speed and magnitude to be effective for postural responses to perturbations. Gait and balance do not require large force contractions but rather require fast bursts of muscle activation. Research has demonstrated that exercise focusing on agility (fast-paced dynamic movements) resulted in faster step reaction times and earlier muscle onset latencies to a force platform translation perturbation than stretching and weight shifting exercises. This suggests that stroke rehabilitation would benefit from the incorporation of fast muscle contractions. Research has been conducted that examined whether exercise geared to improving the speed and pattern of muscle activation was effective in retraining the muscle activation patterns required for standing balance. A single session of exercise retraining with voluntary closed kinetic chain exercise that emphasized speed of movement induced short-term changes in the bursts of muscle activation which transferred to standing balance tasks. Preliminary findings from a 12 session closed kinetic chain protocol on four subjects post-stroke revealed that muscle activation and speed of movement improved over the 4 weeks (12 sessions). The central nervous system used a variety of mechanisms to improve muscle activation patterns needed for postural responses following stroke; this variety would be important, given the inherent heterogeneity in stroke impairments across patients. Therefore, there is a growing body of evidence to support the incorporation of the retraining of speed and power into neurorehabilitation.

This session will be an interactive lecture format, with video observation and participant discussion.

**Conclusions:** This session aims to advance the rehabilitation of mobility and balance impairments following stroke to decrease the risk of falls and increase community mobility for individuals post-stroke. The session will explore the effects of stroke on the ability to produce fast movements required for mobility and dynamic balance. Research which has been conducted to investigate the impact of retraining fast movements required for balance and mobility following stroke supports the implementation of this additional aspect of rehabilitation. Suggestions for treatment techniques to retrain fast movements will be provided to facilitate application of this current research to the neurorehabiliation setting.

# Abstract: P004 – DEFINING THE EARLY INTERDISCIPLINARY REHABILITATION PROGRAM FOR ADULTS FOLLOWING SEVERE AND MODERATE BRAIN INJURY

**Published:** 2011-07

**Authors:** EA Swain, M Neiforth

**Affiliations:** * Neuro-Ability Rehabilitation Services; † Vancouver Coastal Health, Vancouver, BC

**Learning Objectives and Session Content:** 1) To discuss the foci of the rehabilitation program provided to adults during the first 3 months following severe or moderate brain injury based on the results of a national interdisciplinary survey; 2) To collaborate regarding evaluation of patient progress pertaining to the nine functional components of this early intervention program; 3) To facilitate interdisciplinary collaboration integral to successful implementation of the early intervention program.

**Relevance to Physiotherapy Profession:** Standardized practice guidelines do not exist for the rehabilitation of individuals during the first 3 months following severe and moderate brain injury. In addition, standard measurement tools traditionally used to evaluate rehabilitation intervention have a floor effect with this population. Evaluation of client progress, and hence access to services and potential future function, is therefore dependent upon both the identification of the functional components defining foci for assessment and identification of suitable measurement tools. An interdisciplinary national survey identified nine functional components as the foci of the early intervention program. This preliminary step can provide the basis for the identification or development of appropriate measurement tools sensitive to recovery in functional components relevant to this phase of recovery and for the development of national standards of practice.

**Target Population:** All therapists working with individuals following moderate and severe brain injury throughout the care continuum.

**Description of Supporting Evidence:** There is a paucity of research related to rehabilitation intervention of individuals during the first 3 months following severe and moderate brain injury. Research pertaining to treatment of this population is generally focused on medical issues related to mortality and morbidity. Rehabilitation research has explored questions more relevant further along the care continuum. However, during the first 3 months many are unable to participate at a level sensitive to measurement by traditional functional independence measurement tools and issues pertinent to community integration are not yet of prime relevance. This session examines the findings of an interdisciplinary national survey of paired comparisons that establish the foci of the early rehabilitation program.

**Description:** This session will be presented by a physical therapist and occupational therapist. A lecture component will present the national survey findings and include video clips to provide insight into the family and client perspectives of early rehabilitation. This will be followed by facilitated discussion regarding the findings, their implications, and future action.

**Conclusions:** Measuring change for individuals with severe and moderate brain injury is often very difficult especially during the earlier stage of recovery and yet can significantly impact access to appropriate services. A national survey of physical therapists, occupational therapists, and speech-language pathologists determined consensus on nine functional components of the early rehabilitation program during the first 3 months following severe and moderate brain injury. Evaluation of recovery can now be facilitated by identification of measurement tools for these functional components that are sensitive to change by this population. The survey secondarily indicated the importance of the interdisciplinary team to consider all functional components, including those that are not discipline specific.

# Abstract: P005 – SEDENTARY BEHAVIOUR: WHAT IS IT AND HOW DO WE MEASURE IT?

**Published:** 2011-07

**Authors:** E Gorman, EM Macri, MC Ashe

**Affiliations:** Centre for Hip Health and Mobility and UBC Department of Family Practice, Vancouver, BC

**Learning Objectives and Session Content:** This session will provide an overview of the available evidence related to sedentary behaviour in adults and older adults. There will be a demonstration to highlight the use of activity monitors and their potential use in physiotherapy research and practice. The learning objectives for this session are:

1. To define key concepts related to sedentary behaviour;
2. To report the relation between time spent in sedentary behaviour and key health outcomes; and
3. To describe two ways to measure sedentary time and physical activity.

**Relevance to Physiotherapy Profession:** Physiotherapists play an important role in health promotion through active living. New evidence highlights that too much sitting can result in detrimental health outcomes, independent of participation in physical activity. Physiotherapists therefore play a key role in translation of this important knowledge.

**Target Population:** This presentation is relevant to physiotherapists working in research and/or clinical practice. In particular, it is relevant for physiotherapists who work in the areas of health promotion, exercise prescription, and chronic disease management.

**Description of Supporting Evidence:** Physical activity is an essential part of healthy living, yet many Canadians do not meet guideline recommendations. Further, emerging literature from large cohort studies suggests that independent of physical activity levels, time spent in sitting can have adverse consequences on health outcomes. Owen and colleagues define sedentary behaviours as activities that involve low energy expenditure (<1.5 METs) and which include prolonged sitting in transit, at work or for leisure (Owen, 2009). This is distinct from not meeting physical activity guidelines. Unfortunately, there is often confusion in the literature, with many of the same terms used interchangeably to describe the absence of physical activity and time spent engaging in sedentary behaviours. These behaviours are distinct, as it is possible to be both active (meeting physical activity guidelines) and to still spend a large proportion of time in sedentary behaviours. Research suggests that there may be different physiological mechanisms for how these two distinct behaviours influence cardio metabolic health (Hamilton, 2007).

In research, sedentary time is often operationalized as self-reported sitting time or television time. Although self-report measures of activity still play an important role, advances in technology now permit the objective measurement of patterns of physical activity and sedentary behaviour. Accelerometers are devices worn at the waist to capture movement and to provide very detailed time-stamped activity patterns, resulting in a more comprehensive description than self-report alone. Accelerometry analysis software provides valuable information on the intensity of the activity, the length of time of the activity, how many “bouts” the person spent in light or moderate activity [>10 minutes is considered a bout for accumulation of moderate to vigorous physical activity (MVPA) to meet guideline recommended levels], as well as how long the person remained stationary. Further, using this technology researchers have been able to show that the pattern of sedentary time accrual is important for health, in addition to the total time spent in sedentary behaviours (Healy, 2008). This provides evidence for not only the importance of an overall reduction in sedentary time but also the need for breaking up sedentary time. This new technology therefore provides a better characterization of time spent in different types of activity, and thus allows us to draw associations between activity patterns and health outcomes.

**Description:** We will provide an overview of the current evidence and highlight the relevance to physiotherapists; we will provide a practical demonstration of accelerometers and a description of the type of information drawn from these instruments. We will also have smaller breakout sessions to discuss strategies to reduce sedentary time and encourage activity in two age groups: working adults and older adults.

**Conclusions:** Time spent in sedentary behaviours is quickly emerging as an independent risk factor for adverse health outcomes. As the field emerges, physiotherapists are key to develop strategies to reduce overall sedentary time and provide exercise prescription to optimize health. This session will provide an understanding of the current literature, clarify terminology and discuss objective measurement of activity patterns, to assist in the future development of prevention programs.

# Abstract: P006 – DEVELOPMENT OF A STANDARDIZED LYMPHEDEMA ASSESSMENT AND SURVEILLANCE PROGRAM IN ALBERTA

**Published:** 2011-07

**Authors:** ML McNeely, CJ Peddle

**Affiliations:** University of Alberta, Calgary, AB

**Learning Objectives and Session Content:** Objectives 1. To provide a brief overview and update on breast cancer related lymphedema and its treatment. 2 To provide an overview of the methods used to develop a standardized assessment and surveillance program for breast cancer related lymphedema among participating rural and urban centres in Alberta. 3. To share the results of the first phase of the program that focused on the development of an evidence-based standardized assessment and surveillance program to be pilot tested (Phase II) in various sites in Alberta.

**Relevance to Physiotherapy Profession:** Lymphedema is a prevalent and often feared complication of breast cancer treatment. Lymphedema is a chronic swelling of the upper limb on the surgical side which may present immediately or many years after treatment (Petrek, 2001; Clark, 2005). The estimated incidence of breast cancer related lymphedema is reported between 6% and 30%, with higher incidence rates found in studies with longer follow-up (Sclafani, 2008). Lymphedema is a known consequence of surgical and radiotherapeutic techniques. Moreover, numerous factors such as older age, venapuncture to the limb and obesity have been implicated in lymphedema onset (Heyler, 2010). Recent research has demonstrated effectiveness of active surveillance programs to detect and treat lymphedema in the early stages (Stout-Gergich, 2008). When treated conservatively in the earliest stages, complications of lymphedema may be diminished or reversed (Stout-Gergich, 2008). Physiotherapists, as primary providers in the rehabilitation of breast cancer patients and survivors, are well positioned to serve as leaders in the area by providing evidence-based lymphedema assessment and treatment programs in clinical practice.

**Target Population:** This session will be of interest to physiotherapists, students, researchers and other health professionals interested in the rehabilitation of patients and survivors with breast cancer and individual suffering from lymphedema.

**Description of Supporting Evidence:** In Alberta, the need for a province-wide approach to the assessment and surveillance of breast cancer related lymphedema was identified. The primary purpose of the initiative was to support an evidence-based practice approach across urban and rural centres to improve the quality and effectiveness of care for breast cancer related lymphedema.

Key objectives of the initiative:

1. Develop a standardized and comprehensive assessment and follow-up program for Alberta patients presenting with breast cancer related lymphedema.
2. Improve access to comprehensive assessment services for breast cancer related lymphedema.
3. Develop an integration relationship with treatment providers for early intervention and ongoing management.
4. Disseminate information from the assessment and surveillance program to other healthcare providers who may be involved in the subsequent care of the patient/survivor.

Phase I of the project involved developing a standardized evidence-based approach to the assessment of lymphedema between the participating centres. Components of program that were deemed relevant for the program included:

1. Screening for lymphedema
2. Baseline assessment of lymphedema: methods and outcomes for upper limb size and volume, upper limb range of motion, pain assessment, appropriate outcome measures to assess upper limb function and quality of life
3. Regular follow-up (surveillance program) timelines and outcomes
4. Develop an assessment and surveillance tool for documentation of outcomes

The first step in Phase I of the process involved identifying existing centres/community partners and key stakeholders for the project. Key stakeholders interested in participating in the project were identified and these stakeholders included individuals from two urban and three rural centres:

1. Edmonton: Cross Cancer Institute, Rehabilitation Medicine Department.
2. Calgary: Tom Baker Cancer Centre, Lymphedema Program.
3. Medicine Hat: Hospital, Rehabilitation Medicine Department/ Community Cancer Centre.
4. Lethbridge/ Pincher Creek region: Hospital, Physical Therapy Department/ Community Cancer Centre.
5. Red Deer: Hospital, Physical and Occupational Therapy Departments/ Community Cancer Centre.

Moreover, a number of stakeholders agreed to serve as consultants on the project including surgeons, oncologist and administrators from various sites.

The second step involved performing literature searches on key topics related to the project (e.g., validity and reliability of outcome measures and measurement methods), synthesizing the information, and disseminating the information to participating team members. A meeting was held with the project researchers and representatives from the participating centres to determine the relevant components of the proposed assessment program and how these components could be best captured. Issues and barriers related to the implementation of assessment components as well as the standardized program were identified. The next step in the process involved finalizing components of the program and developing a draft assessment tool in preparation for the Phase II pilot testing of the program at the participating centres.

**Description:** The session will be a lecture format. The session will start with an overview of breast cancer related lymphedema and the treatments used in the management of this chronic condition. As well, I will discuss international and national initiatives that are currently underway to improve the care of individuals with lymphedema (20 minutes). Following the overview, I will describe the methods used to develop the standardized assessment and surveillance program among participating rehabilitation medicine departments across Alberta (20 minutes). The session will then focus on the evidence used to inform decisions on the assessment program including the assessment tool, the decision-making process, and the barriers identified, and the strategies put in place to facilitate implementation of the program in the clinical setting (20 minutes).

**Conclusions:** In the last couple of years, there has been increased recognition at international (International Lymphedema Framework) and national levels (Canadian Lymphedema Framework), for the need to 'advance comprehensive effective assessment and treatment for lymphedema and related disorders to all persons'. Thus, this project, although specific to the breast cancer population, is line with current international and national efforts to improve the care of individuals with lymphedema.

# Abstract: P007 – ACUPUNCTURE IN ACUTE CARE ONCOLOGY

**Published:** 2011-07

**Authors:** O Cheifetz

**Affiliations:** Hamilton Health Sciences, Hamilton, ON

Learning Objectives and Session Content:

- To broaden participants knowledge about the role of acupuncture for people diagnosed with cancer.
- To review evidence related to acupuncture and cancer.
- To review the challenges of utilizing acupuncture for people with cancer in the acute care setting.
- To engage the audience in a discussion on strategies to increase the utilization of acupuncture in the acute care setting.

**Relevance to Physiotherapy Profession:** As primary care providers, physiotherapist may encounter people diagnosed with cancer in most acute care settings. Since cancer treatments are improving, many patients will survive their cancer treatment and will have to manage the side effects of the primary disease, as well as, those created by cancer treatment. Evidence is available supporting the use of acupuncture to alleviate several side effects associated with cancer and its treatments. However, physiotherapists are not regularly requested to provide this service for people with cancer in the acute care setting. It is imperative that physiotherapists have a basic understanding of the potential benefits of acupuncture for people diagnosed with cancer, so that the most effective treatment can be utilized to assist in the management of observed symptoms. It is also important the physiotherapists are able to demonstrate leadership in managing patient's cancer related side effects.

**Target Population:** This workshop is appropriate for physiotherapists and other health care providers who interact with people diagnosed with cancer. This information is aimed at care providers primarily in acute care but also to those in the community (both private practice and home care).

**Description of Supporting Evidence:** Acupuncture is defined as “a therapeutic and/or preventive medical procedure performed by the insertion of one or more specially manufactured solid metallic needle(s) into specific location(s) on the body.” Since its introduction to the Western World from Ancient China, acupuncture has evolved to include various forms, including electro-acupuncture. While acupuncture is an invasive modality, adverse effects of acupuncture rarely occur. The risk of a serious adverse reaction is approximately 0.05 per 10,000 treatments. Reported side effects range from local skin irritation to pneumothorax and death, and can include central nervous system injury, infection (most commonly hepatitis B), and extreme drowsiness. Although the overall incidence of these side effects is very small, they can be avoided if proper precautions are taken, such as using appropriate sterile techniques and ensuring adequate acupuncturist training. Recent research has found that acupuncture may aid in reducing side effects of cancer treatment. Specifically, benefits were found in the management of nausea, pain, fatigue, and chemotherapy related hot flashes. Relevant literature will be presented during the pesentation and references will be provided to participants.

**Description:** This interactive workshop, presented by a physiotherapist, will include podium presentation and open discussion with the audience. Participants will be encouraged to participate throughout the session to help identify strategies to address issues discussed.

**Conclusions:** As therapies to manage cancer and its treatment side effects improve, physiotherapists need to have different modalities to assist patients who are having difficulties with complications such as pain, numbness, or other symptoms. Acupuncture is a modality that physiotherapists are able to utilize, however its use with people diagnosed with cancer may be challenging, especially in the acute care hospital setting. Physiotherapists participating in this workshop will have a better understanding of the potential benefits of acupuncture for people diagnosed with cancer. Therapists will also have a stronger understanding of the evidence supporting the use of acupuncture, safety recommendations, and challenges of using this modality in the acute care setting. Participants will contribute to the development of a “tool box” that can be used to educate health care providers, patients, and others on the use of acupuncture by physiotherapists in the acute care setting to manage cancer related side effects.

# Abstract: P008 – HOME CARE ASSESSMENT (RAI-HC): SUPPORTING POLICY, PRACTICE AND RESEARCH

**Published:** 2011-07

**Authors:** K Berg, J Arthur, C Beach

**Affiliations:** * University of Toronto,Toronto; † TC-CCAC, Toronto; ‡ VIHA, Victoria, BC

**Learning Objectives and Session Content:** The RAI-HC is a multi-dimensional assessment used in most Canadian provinces to determine eligibility for home care and service planning for individual patients. Organizations have used aggregated RAI-HC assessments to identify subgroups and inform new models of home care delivery. Home care aggregate provincial RAI-HC data have permitted analyses of the effect of receipt of PT or OT on multiple outcomes using Markov modeling. After adjusting for baseline multiple morbidity, falls, functional status, frailty/instability, age and gender, clients with ADL deficits were more likely to become more independent, less likely to be admitted to hospital or long term care, less likely to die and more likely to be discharged without services. Such analyses provide important evidence for the value of home care PT and can assist in advocating for increased resources. National or provincial RAI databases may provide evidence for other practice related questions. Greater familiarity with the content and potential uses of the assessment will assist PTs in optimizing the existing health information to advance and justify their practice.

The learning objectives of this session are:

1. To enhance PTs understanding of the RAI-HC and its potential uses
2. To enhance PTs understanding of population based models of service delivery
3. To enhance PTs use of existing health information and to consider system level outcomes

**Relevance to Physiotherapy Profession:** PTs have strong measurement skills but tend to focus on outcomes of single patients. Little attention has been placed on demonstrating value of PT to the health system. This session aims to stimulate this interest and challenge them to optimize use of existing home care data and contribute as needed to further develop the database with outcome information.

**Target Population:** Home and community PTs but those in LTC may also be interested given the close relationship between the RAI-HC and MDS 2.0.

**Description of Supporting Evidence:** In addition to the information provided in the content section above, there are numerous articles on the RAI-HC that address reliability of the items and the validity of various sub-scales. The interRAI.org website includes over 300 articles based on the RAI-HC and related assessments. CIHI uses the RAI-HC data for public reporting. There are manuals published explaining how to score the RAI-HC and Clinical Assessment Protocols (CAPS) suggesting treatment approaches. Population Health approaches developed by OACCAC and Toronto Central CCAC (Community Care Access Centre) continue to be explored in Ontario.

**Format:** We will present 3 fairly brief presentations: a description of the assessment and its uses and the results showing the benefit of the RAI-HC, a description of the TC-CCAC population based approach based informed by RAI-HC data and a new initiative on Vancouver Island to engage rehab professionals in completing RAI outcomes. The sessions will be interactive and participants will be actively engaged in exchanging practice ideas and suggesting future initiatives.

**Conclusions:** RAI-HC provides valuable information on client needs and outcomes and is used to support clinical and policy decisions. The more therapists are aware of the possibilities, the more they can influence decisions and use the data to advocate for patient care. They will also be able to take better advantage of existing RAI-HC databases to answer practice questions, particularly for relatively rare populations.

# Abstract: P009 – ELECTRONIC HEALTH RECORDS IN PHYSIOTHERAPY PRACTICE: WHY GOING GREEN ALSO MAKES GOOD BUSINESS SENSE

**Published:** 2011-07

**Authors:** K Houde, S Funk

**Affiliations:** University of Ottawa Health Services, Ottawa

**Learning Objectives and Session Content**: Electronic Health Records (EHRs) have been in existence since the 1960's. While there have been significant advances in both technology and electronic communication methods over the last five decades, most healthcare professions have been slow to make the move to paperless records.

This presentation will provide an overview of current EHR legislation and trends at the global, national, and provincial levels as they relate to the healthcare field, and more specifically, physiotherapy practice.

In addition, research-based guidelines will be proposed for the selection, implementation, and use of EHRs in the clinic setting. Presenters will outline the potential benefits to the clinic owner, health professional, and patient in using a paperless system in the clinic. Drawing from personal experience and from case studies, presenters will propose solutions to overcome the perceived barriers to implementing an EHR system in the workplace.

**Relevance to Physiotherapy Profession:** Physiotherapists are lagging behind other health professionals, i.e. doctors and pharmacists, in adopting EHR systems in their practices. As more and more governments consider mandating the use of EHRs in the coming years, physiotherapists should consider implementing EHR systems into their practice in order to stay on pace with current and future legislative requirements.

Implementing EHRs can be a challenge. However, this presentation will demonstrate that in addition to decreasing a clinic's environmental footprint and saving staff time and money, other benefits of implementing EHRs, for example, the ability to communicate effectively with other health professionals, monitor patient progress, and improve patient outcomes far outweigh the temporary inconveniences caused by the move to a paperless system.

**Target Population:** A prior level of exposure to this topic is not required. This session is targeted to anyone in healthcare with an interest in electronic health records, including private practice clinic owners, public and private administrators, employees, and contractors.

**Description of Supporting Evidence:** To provide participants with an overview of the current legislation regarding the use of EHRs by healthcare professionals, publications from healthcare associations and medical colleges, as well as governmental agencies such as the Canada Health Infoway will be cited.

Systematic reviews and case studies supporting the use of electronic health records in physiotherapy will be presented to assist clinicians to more clearly understand the benefits to EHR implementation in their practice.

Lastly, evidence-based guidelines in practice management will be proposed to assist participants in addressing and overcoming the barriers to EHR implementation.

**Description:** This session will be in lecture format and will include a question and answer period following the presentation that will allow participants to share their perspectives and experiences with EHRs.

**Conclusions:** Upon completion of this session, participants will:

- Gain a better understanding of the current global, national, and provincial legislation and trends in the use of EHRs in physiotherapy practice
- Make informed decisions regarding the selection and implemention of an EHR system in their clinic or department
- Understand how the use of EHRs in their practice will save them time and money, reduce their environmental footprint, facilitate communication with other health professionals, and improve patient outcomes.

# Abstract: P010 – INTRAPROFESSIONAL RELATIONSHIPS: SUPPORTING QUALITY PRACTICE

**Published:** 2011-07

**Learning Objectives and Session Content:** 1) To discuss how learning needs were defined and resources developed to support intraprofessional relationships 2) To describe how these resources are accessible to and utilized by physiotherapists and physiotherapist support personnel 3) To discuss potential ways in which patients will benefit from this effort.

**Relevance to Physiotherapy Profession:** Across Canada, it is recognized that health care dollars continue to stretch requiring facilities and providers to do more with less. At the same time the Canadian population is aging, with expectations that demands on health care will rise. Innovative models of care are sought out to deliver necessary health care services. With regards to physiotherapy, physiotherapist support personnel help to ensure that the public continues to have access to physiotherapy treatment in all practice settings.

As a regulatory organization we are aware of the growing importance of physiotherapist support personnel within the health care team and we recognize and aim to assure that patients receive quality care. This purpose includes minimizing risks and optimizing patient safety. By developing and providing resources for physiotherapists and physiotherapist support personnel, the College endeavours to strengthen and support these working relationships in the interests of the public.

**Target Population:** This session will be of interest to clinicians, professional practice leaders, facility managers, and physiotherapist support personnel committed to ensuring safe, quality care for the public.

**Description of Supporting Evidence:** In 2008 an extensive review of the material available through provincial, national, and international organizations related to the use of physiotherapist support personnel was completed. Following the analysis of this information, a focus group of physiotherapists and physiotherapist support personnel were consulted to find out how their current working relationships were perceived by team members. As a next step a survey was distributed to all physiotherapists within the province with a request that they share this survey with physiotherapist support personnel within their team. The survey yielded responses from over 700 physiotherapists and 400 physiotherapist support personnel. In addition to insights related to working relationships, this survey also helped inform the organization of what type of learning resources would be most valued as learning tools.

Additionally, data collected from various program areas within the organization indicated an increase in the number of questions and concerns related to the utilization of physiotherapist support personnel by physiotherapists. Questions and concerns were raised by patients, physiotherapists, physiotherapist support personnel, other team members, employers, and funders.

Based on this information, a program was developed to target resources to strengthen the working relationships of physiotherapists and physiotherapist support personnel at the point of care. The program is multi-year with an evaluation strategy that will enhance its ongoing development and implementation.

**Description:** This session will be a lecture format with opportunity for participants to ask questions and share experiences regarding their team dynamics.

**Conclusions:** Physiotherapist support personnel are valuable contributors to the team and they are increasingly used to deliver physiotherapy services. As physiotherapists assigning care to physiotherapist support personnel, it is important to ensure quality care and safety while minimizing the risk of harm. The information collected leading into this program emphasized focus on the relationship between the team members.

Through the initial phases of this program, feedback is extremely positive from physiotherapists, physiotherapist support personnel, and team leaders regarding the usefulness of the currently available tools and resources. Ongoing feedback linked to resources will be obtained in addition to collecting data from other College programs and questions raised through these programs. In the future, stakeholders will once again be surveyed.

# Abstract: P011 – PROFESSIONAL PRACTICE PORTFOLIOS: A MUST TO SUPPORT PHYSIOTHERAPISTS AND THEIR PRACTICE NEEDS

**Published:** 2011-07

**Learning Objectives and Session Content:** Four years ago, The Ottawa Hospital started its journey to remodel its professional practice structure. As a bi-product, these roles were put in place in addition to the Chief role: 5 Professional Practice Coordinators, an Advanced Practice Physiotherapist, 5 Senior Physiotherapists (change in role), Clinical Specialist and Clinical Experts. Once these new leaders were recruited, trained and were competent in their role, it had now become the time to align the new Physiotherapy Leadership team at the Ottawa Hospital to professional practice portfolios. These portfolios required a centralised alignment to ensure that all physiotherapist at the hospital, independent of their location of work (campus, site, unit) or status (full time, part time, casual) could have their professional practice needs met. These portfolios also needed a localised approach so that the delivery and final product would be tailored to meet the individual need relative to the clinical setting and the patient population. Following an extensive consultation process, 19 core professional practice portfolios were developed: service excellence; role development; partnerships; research strategy; caseload management strategy; performance management and leadership development; best practice/knowledge transfer; standards of care; CQI; orientation; the new staff experience; the support staff; the clinical council; the student experience; education; communication; equipment; casual employee experience; mentorship, link with orthopedics (triage, pre, post surgery).

Participants will be able to successfully navigate these learning objectives:

1. Be familiar with the new professional practice structure at the Ottawa Hospital
2. Understand the steps in determining corporate portfolios and core elements of the portfolios at the Ottawa Hospital
3. Understand the importance of centralised planning to standardise and tailor delivery to better meet the needs of all physiotherapists (all areas)
4. Understand how to set yearly targets/objectives with the portfolios and measurement to meet outcomes (practical application adapted to the participants workplace setting).

Participants will learn from the Ottawa Hospital experience how to develop clear professional practice portfolios to guide professional practice with reachable targets and benchmarks adapted to their workplace setting that raises the support provided to physiotherapists and allows more therapists to move from novice clinicians to proficient and expert clinicians more quickly.

**Relevance to Physiotherapy Profession:** The content of this presentation is very relevant to clinicians and organisations. Some therapists who have 15 years of experience for example, have in some case truly only 1 year of experience repeated 15 times. A solid professional practice program using portfolios allows clinicians to look within, identify gaps and develop strategies to improve performance. Smaller organisations or private clinics do not have the structure or the resources to successfully develop from scratch elements of professional practice which includes professional practice portfolios. The Ottawa Hospital has a total of 250 people working within the profession which includes 200 physiotherapists and 40 physiotherapy/rehabilitation assistants. To support these professionals, 7 leaders, most of whom do not carry a clinical load are aligned to the portfolios to support the staff's professional needs. As a result a higher number of our clinicians are able to progress quicker from novice physiotherapists to expert clinicians.

This presentation will benefit clinicians in both hospital and private settings, and this tool can easily be used by smaller organisation so elements can be incorporated within their organisation.

**Target Population:** The target populations include: 1) leaders from large academic centers that can use all of the elements discussed to successfully implement all of the portfolios; 2) leaders from smaller organisations or private clinical owners/leaders so they can use the elements of the portfolios that are the most relevant to their practice; 3) individual clinicians so they can be aware of all of the components that allow them to become proficient clinicians quicker; 4) researchers and academics so that further studies can be developed that look at cause and effect relationships between the level of professional practice support (portfolios) and actual clinical performance.

**Description of Supporting Evidence:** When we prepared for the re-structure in 2008, an extensive review of the literature was carried out using the internet and Medline search engines using the following keywords: review of program management, centralization, decentralization, Physiotherapy and Occupational Therapy. Through this review, it became evident that very few studies were conducted to compare measurable impact before and after decentralization. Most articles consisted of anecdotal commentary, case studies or the utilization of qualitative structured interviews to assess and publish the impact of decentralization on various Health Professional groups. Still we were able to extract some important common elements from the material reviewed.

The negative points of decentralization identified through the review of articles include a decrease in clinical involvement, fewer opportunities for professional support and leadership, lack of professional career laddering, lack of professional status, lack of professional development activities, sense of loss, limited resources for orientation and mentoring of new staff, less participation in research and publications, less flexibility in resource management, lack of standardized clinical policies and procedures, loss of the operational control of supplies and equipment, less efficiency at maintaining standards of practice, and decreased staff retention.

This is the case in many of our clinical settings today and a strong professional practice structure with clear portfolios allows for physiotherapists and support staff to be well supported independently of the management structure they are currently in.

The evidence is quite clear that within the clinical setting, health care professionals require a solid professional practice program that provides the needed practice support, links with therapists that are expert clinicians, helps further develop the clinical and non clinical skills required to be successful and to have an impact with patients/families, and other care providers.

**Description:** The first portion of the presentation will be delivered in a podium type fashion to address these various topics: 1) Summary of the new professional practice structure at the Ottawa Hospital; 2) Discussion of the process to engage staff, get feedback, formulate common themes and develop 19 core professional practice portfolios; 3) Give examples of the development of yearly goals with a few portfolios. The second part of the session will be interactive with the audience participating in priority setting in groups, developing key portfolios related to their own workplace and addressing a few goals within each portfolio. It is anticipated that each participant will be able to take back a high level plan to implement professional practice portfolios in their workplace.

**Conclusions:** The change in the Physiotherapy Professional Practice Structure at the Ottawa Hospital was presented at Congress 2008, but that was only the first pillar. The first tangible product that has been developed thanks to this new structure has been the professional practice portfolios.

As was true with the new structure itself, the methodology used to communicate, gather data and ideas was key to develop our 5 year strategic plan, develop the key professional practice portfolios and develop a process which consists of setting clear yearly objectives with targets for each of the portfolios. This work and sharing the data will help smaller organizations and clinics to utilize the data and processes developed at the Ottawa Hospital to adapt to their clinical setting. This will lead to greater professional practice support for physiotherapists in various settings and will help more clinicians progress more quickly from novice clinicians to proficient and expert clinicians and leaders.

# Abstract: P012 – THE SUM OF THE PARTS: STRUCTURE, ROLES AND OUTCOMES OF THE PHYSIOTHERAPY ASSOCIATION OF BC'S 'KNOWLEDGE TEAM'

**Published:** 2011-07

**Authors:** AM Hoens, R Tunnacliffe, D Monkman

**Affiliations:** Physiotherapy Association of British Columbia, Vancouver

**Learning Objectives and Session Content:**

1. To provide a brief review of the need and challenge to provide knowledge support and translation for evidence-informed practice.
2. To provide a description of structure, roles and outcomes of the Physiotherapy Association of BC's unique Knowledge Team
3. To provide examples of specific strategies and projects undertaken by the Knowledge Team.
4. To discuss the implications of the Physiotherapy Association of BC's Knowledge Team on evidence-informed practice for physiotherapists in BC.

**Relevance to Physiotherapy Profession:** The importance of knowledge translation in support of evidence-informed practice is well acknowledged by academics, decision-makers and clinicians. Given the complexities and challenges of knowledge translation and limited success of existing support strategies, novel approaches are required to enhance the adoption of best practices.

**Target Population:** This session will be of interest to physiotherapists and profession leaders who are seeking innovative strategies to enhance physiotherapy education, research and practice targeted to support evidence-informed practice.

**Description of Supporting Evidence:** Despite the well recognized need for and challenges to evidence-informed practice, physiotherapists, like all healthcare professionals, struggle with identifying and implementing strategies to successfully address these issues. Increasingly, there is evidence to support the combination of multiple strategies which address different aspects of the issue. Accordingly, the Physiotherapy Association of BC developed a ‘Knowledge Team’ – a librarian, a knowledge broker and a specialist in technological support. Collectively, these three positions encourage and support evidence-informed practice, promote greater technical literacy, and foster a sense of community amongst approximately 2,000 members. This session describes how the knowledge team successfully addresses, using new technologies like on-line tutorials, webinars and social media, many of the well known barriers to evidence-informed practice such as: access to evidence, evaluation of evidence and application of evidence. Extensive support of the supporting literature will be woven throughout the presentation.

**Description:** This session will involve a lecture format with specific examples of strategies and the results of the evaluation of their effectiveness. Active participation by attendees will be elicited through a variety of pedagogical techniques.

**Conclusions:** The Physiotherapy Association of BC Knowledge Team is a novel, unique and effective support for enhancing evidence-informed practice for BC Physiotherapists. Engaging members in the process and supporting uptake in practice has proven to be a successful member service. Sharing the structure, roles and outcomes of this team will provide participants with a framework for enhancing evidence-informed practice within their own settings.

# Abstract: P013 – PROVIDING EFFECTIVE FEEDBACK: EVERYTHING YOU'VE EVER WANTED TO KNOW

**Published:** 2011-07

**Authors:** B Mori, C Evans

**Affiliations:** Department of Physical Therapy, University of Toronto, Toronto

**Learning Objectives and Session Content:** The focus of this interactive session will be to define effective feedback and equip participants with a framework to provide effective feedback. Upon completion of this session, attendees will be able to:

1. Define and explain why feedback is important.
2. Describe the elements of effective feedback.
3. Employ behaviors used to give feedback.

**Relevance to Physiotherapy Profession:** As physiotherapists, we provide feedback in our everyday activities usually provided with the aim to improve someone else's performance. We do this with our patients, with our colleagues and with our students. Providing well delivered feedback is a skill, and just like any other technical skill, it needs to be developed and practiced. This workshop will help you develop those skills.

**Target Population:** This workshop would be beneficial to educators, clinical supervisors/instructors and those who are required to provide feedback to others on a regular basis.

**Description of Supporting Evidence:** The literature supports that well delivered feedback has many advantages such as promoting learning, developing skills, achieving competence, improving self-assessment skills and maintenance of competence (Veloski et al., 2006). The literature also tells us that most individuals demonstrate poor self-assessment skills, especially in those who are the least competent and those who are highly competent (Kruger and Dunning, 1999). This trend has also been demonstrated in health professionals (Davis et al., 2006). While being able to deliver effective feedback, especially with those who are lacking competence, has the potential to vastly improve performance, there is the idea that providing well delivered feedback takes time and can be emotionally taxing and create a sense of discomfort. There are several models to facilitate providing well delivered feedback such as the Six Stages of Effective Feedback (Brown et al., 1995), Hewson & Little's Feedback Model (1998), Chicago Model (Brukner et al, 1999), and the Relative Ranking Model (Regehr et al., 1996). These models aim to simplify the process of providing well delivered feedback to better enable the development of this skill. This workshop will focus on 2 of these models.

**Description:** This learning session will integrate a variety of didactic and interactive techniques including audience participation, sharing experiences, think-pair share, DVD teaching clip debriefing and role play activities. Participants will leave with teaching tip cards to facilitate applying content from the session to their everyday practice. It will conceptually combine elements of research, education and practice. Participants will draw upon their own practice to identify applications in their settings.

**Conclusions:** By providing this learning opportunity, we hope to enhance the knowledge, skills, attitude and practice of participants to provide effective feedback within and beyond the physiotherapy profession.

# Abstract: P014 – BREAKING DOWN THE MIND-BODY BARRIER: THE MECHANISMS OF PAIN CATASTROPHIZING AND CLINICAL IMPLICATIONS FOR PHYSIOTHERAPY

**Published:** 2011-07

**Authors:** TH Wideman, DM Walton

**Learning Objectives and Session Content:** Upon completion of this education session participants will:

1. Be able to describe and measure the construct of pain catastrophizing;
2. Understand the importance of pain catastrophizing for physiotherapy practice;
3. Understand psycho-neuro-endocrine mechanisms through which pain catastrophizing influences healing and the pain experience;
4. Be apprised of current evidence-based management strategies that physiotherapists can use for individuals with elevated levels of pain catastrophizing.

This education session will focus on providing participants with an overview of recently published research on pain catastrophizing. An emphasis will be placed on literature that addresses the psychological, neurophysiological and endocrinological mechanisms and clinical implications of elevated levels of pain catastrophizing in people with pain. Research that has linked levels of pain catastrophizing to the following maladaptive responses to pain will be discussed: dysfunctional immune/inflammatory response, amplified neural activation and inaccurate perceptions of ability. The session will link these influences to the development of chronic pain and/or pain-related disability across a variety of pain conditions. Clinical research examining the role, and measurement, of pain castastrophizing as a risk factor for problematic recovery will be highlighted. The evidence-based management of patients with elevated levels of pain catastrophizing will be discussed.

**Relevance to Physiotherapy Profession:** Over the past ten years, pain catastrophizing has emerged as one of the most robust predictors of prolonged pain and disability. Pain catastrophizing, defined as a maladaptive orientation to pain, is characterized by pain-related thoughts of helplessness (e.g. “There's nothing I can do to reduce the intensity of the pain”), symptom rumination (e.g. “I keep thinking about how much it hurts”), and threat-value magnification (e.g. “I wonder whether something serious may happen”). Previous research has linked elevated levels of pain catastrophizing to heightened levels of pain intensity, impaired physical function and increased pain behaviour. While pain catastrophizing was originally characterized as a purely psychological construct, emerging research suggests that this factor also plays an important role in the physiological mechanisms of pain. For example, levels of pain catastrophizing have been linked to physiological markers of the inflammatory response, activity in the hypothalamic-pituitary-adrenal axis, and function of higher-order brain functions. Together, this research suggests an intimate link between psychological and biological aspects of pain. This literature can therefore help physiotherapists understand the pathway from high catastrophizing to persistent pain and disability. By integrating knowledge of both the biological and psychological dimensions of pain, physiotherapists stand to improve both their prognostic accuracy and treatment outcomes.

**Target Population:** This education session is designed for all therapists that treat patients with pain, and researchers that are interested in the subject of pain. An emphasis will be placed on research relating to orthopedic pain conditions. No background knowledge in the field of pain research is required.

**Description of Supporting Evidence:** The clinical importance of pain catastrophizing is well supported in the literature. Pain catastrophizing has been linked to levels of pain intensity and pain-related disability across a wide range of patient populations. These relationships have been observed both in cross-sectional and prospective studies, and across different age groups. Clinical measures of pain catastrophizing have been shown to have excellent reliability and validity, and are widely used across healthcare disciplines. Interventions that target pain catastophizing have also been supported by evidence from randomized controlled trials.

Emerging research supports the links between pain catastrophizing and maladaptive neurophysiological responses to pain. Several studies exploring these relationships have been published in high impact, multidisciplinary journals such as Brain, Pain and Psychoneuroendocrinology. The majority of this research is cross-sectional. These findings have been supported with studies using both healthy participants with experimentally induced pain conditions, and people with clinical pain conditions.

**Description:** The format for this education session will include a slide show presentation and opportunities for audience participation. Mr. Wideman and Dr. Walton will present research findings from their own work as well as relevant findings from the work of others in an open seminar format aimed at improving the understanding of the importance of pain catastrophizing, and laying the groundwork for ethicial and judicious clinical behaviour change. Formal knowledge exchange approaches will be undertaken, taking both didactic and interactive forms. The hour will be broken into three broad sections: 1. An overview of pain catastrophizing, including a discussion of the usefulness, scoring and interpretation of the Pain Catastrophzing Scale; 2. A discussion of the mechanisms through which catastrophizing can lead to the development of persistent pain or disability, including psychological/perceptual, neural and endocrine processes; 3. A discussion on the state of the evidence in support of clinical intervention strategies that can be employed by physiotherapists. At least 5 minutes will be reserved for questions, and the floor will be open to discussion throughout the session.

**Conclusions:** Current models of pain emphasize the influential role of biological, psychological and social factors. Research addressing the role of pain catastrophizing effectively cuts across these three domains. This presentation aims to increase the participant's understanding of the clinical importance and physiological mechanisms of this influential factor, thereby improving the clinical management of pain, and improving outcomes for patients.

# Abstract: P015 – Protocol Guidelines for Rehabilitation After Anterior Shoulder Dislocation and Surgical Stabilization

**Published:** 2011-07

**Authors:** M Werstine

**Affiliations:** Fowler Kennedy Sport Medicine Clinic, University of Western Ontario, London, ON

**Learning Objectives and Session Content:** 1. To review the latest evidence on rehabilitation guidelines after anterior shoulder dislocation and surgical stabilization. 2. To apply evidence based rehabilitation guidelines for post operative anterior shoulder stabilization into clinical practice. 3. To use an evidence based anterior shoulder stabilization rehabilitation protocol to assist clinicians (knowledge translation) on decision making for safe return to activity/sport.

In this session, the latest evidence on key domains in anterior shoulder stabilization rehabilitation will be reviewed: Defining commonly used terms (i.e. bankart, Hill-Sachs, type of labral lesions), healing timelines, anatomical structures requiring protection post surgery, range of motion guidelines and precautions, force couples of the rotator cuff, proprioception and quality of movement execution. The protocol guidelines will also be outlined for each of the three phases (0-6 weeks, 6-12 weeks, 2-24 weeks) of rehabilitation post anterior shoulder stabilization. As well, clinical recovery curves for pain and function will be discussed using three different cases.

**Relevance to Physiotherapy Profession:** Full rehabilitation of patients with anterior shoulder instability and surgical stabilization whose vision is to return to sports/functional activities with minimal risk of re-injury can be a challenge. Due to the major advances in surgical techniques for anterior shoulder stabilization, a client's safe return to sport/activity is increasingly influenced by the post stabilization rehabilitation they receive. Despite new information and reviews on all topics related to shoulder instability and stabilization procedures, no standardized protocol has been universally accepted. To utilize best practice guidelines, knowledge translation of the literature and evidence based protocols are essential to the practicing clinician and to research studies.

**Target Population:** Physiotherapists in private practice/out patient orthopaedics, sports, orthopaedics, Kinesiologists/Physiotherapy assistants/support staff, Students and Primary care physicians.

**Description of Supporting Evidence:** A complete search for all articles was performed using Pubmed, Medline, Cinahl and Sport Discus. Keywords used in the search included: shoulder, instability, dislocation, stabilization, Bankhart, capsular shift, labrum, proprioception, EMG, strength, ROM, rehabilitation, surgery, exercise, biomechanics surgical technique, outcome measure. Numerous stabilization protocols already in existence through post graduate courses and from various treatment facilities internationally were attained.

**Description:** Lecture with question/answer at the end.

**Conclusions:** An evidence based rehabilitation protocol for shoulder stabilization after anterior shoulder dislocation has been developed. This will assist clinicians to use best practice guidelines and assist in clinical decision making to optimize safe return to activity/sport in this specific patient population.

# Abstract: P016 – THE PECTORALIS MAJOR MUSCLE: INJURY CLASSIFICATION AND REHABILITATION IMPLICATIONS

**Published:** 2011-07

**Learning Objectives and Session Content:** After completing this education session, participants should be able to:

1. Be conversant in the most recent anatomic and biomechanical evidence on the structure and function of the pectoralis major muscle and the most common patterns and location of injury.
2. Be able to perform a thorough clinical examination to distinguish cases of pectoralis major muscle injury that can be treated conservatively versus those that are appropriate for surgical referral.
3. Be knowledgeable about the alternative surgical repair techniques for rupture of the pectoralis major and their impact on post-operative rehabilitation.
4. Be familiar with a standardized post-operative rehabilitation protocol (types of exercises and phased progression) that has been used successfully to treat patients undergoing surgical repair for ruptures of the pectoralis major.

**Relevance to Physiotherapy Profession:** Once considered to be a rare injury, ruptures of the pectoralis major have increased significantly, with more than half of all reported cases in the literature occurring within the last decade. Over 45% of these injuries are associated with weight lifting activities. Given the physiotherapy movement toward extended scope of practice, physiotherapists may frequently become the first source of referral for diagnosis and treatment of these types of injuries. The ability to accurately identify and treat ruptures of the pectoralis major is aided by an accurate knowledge of the anatomy, clinical presentation, and appropriateness of conservative versus surgical management. Until recently, the actual anatomy of the pectoralis major has been misunderstood, particularly with regard to the number of muscle segments and tendon layers and their lines of action. These factors, along with an increase in surgical cases that require post-operative rehabilitation, can significantly impact a physiotherapist's approach to assessing and treating patients with pectoralis major injuries.

**Target Population:** Orthopaedic physiotherapists

**Description of Supporting Evidence:** A systematic literature review of all reported cases of pectoralis major rupture was conducted by one of the presenters (a manuscript of findings is currently pending review in the American Journal of Sports Medicine). We compiled an analysis of injury patterns, mechanism of injury, clinical presentations, and treatment options. The anatomic and biomechanical overview is based on evidence resulting from a three-dimensional, digitized anatomic study of the pectorlis major performed in collaboration with anatomy researchers at the University of Toronto. Further evidence is provided by the presenters' own experience in assessing and treating an increasing number of pectoralis major injuries as part of an orthopaedic surgeon's upper extremity practice.

**Description:** This will be primarily a lecture format that includes graphics and video clips to demonstrate anatomic findings, clinical presentation and assessment techniques, as well as approaches to specific exercises. We will also use audience volunteers to demonstrate appropriate setup, hand placement, movement errors, etc. during descriptions of assessment and treatment techniques.

**Conclusions:** Increasing interest in fitness and competitive athletics has resulted in an escalation of reported injuries to the pectoralis major muscle. Almost half of these injuries occur while weightlifting. Given that this has historically been considered a rare injury, physiotherapists may lack familiarity with these clinical presentations and how to properly assess and treat them, either conservatively or post-operatively. We provide an evidence-based overview of the complex musculotendinous anatomy of the pectoralis major, a classification of injury patterns, assessment techniques and surgical management alternatives for treating ruptures to the pectoralis major muscle. This information should provide the foundation for a confident approach to clinical diagnosis and post-operative treatment of pectoralis major injuries.

# Abstract: P017 – SACROILIAC JOINT DIAGNOSIS, SCREENING AND SUBGROUPING: SIMPLE SOLUTIONS TO AN OLD CONTROVERSY

**Published:** 2011-07

**Authors:** R Rosedale, A Long

**Affiliations:** * London Health Sciences Centre, 339 Windermere Road, London, ON; † Bonavista Physical Therapy, Calgary, AB

**Learning Objectives and Session Content:** Historically the sacroiliac joint is one of the most controversial joints in the body. This session will summarize decades of debate which has centered around three main themes.

Firstly, reported prevalence rates have varied greatly. Some believe that the prevalence is high and the sacroiliac joint is a common source of pain, others argue that it is rarely the primary source of pain. At one end of the spectrum are groups who feel the sacroiliac joint should always be treated. The rationale is that if it is not the primary site of pain in lumbosacral disorders then it causes pain when its mechanics are faulty. This creates strain that produces pain further up the kinetic chain (lumbar ligaments, discs, facet joints). Others do not accept these models and feel the sacroiliac joint is highly over-diagnosed, is rarely the source of pain and thus only infrequently needs intervention.

Secondly, debate continues in some circles regarding the amount of available movement and the functional relevance of that movement in the sacroiliac joint. Advocates support the concept that movement at the joint becomes dysfunctional when it becomes blocked or excessive or when movement is asymmetrical and this can have major implications for pain and function. Opponents to this view argue that there is no meaningful range of motion at these joints and they call into question both the ability to detect minute changes in these motions and the clinical relevance of such changes.

Finally, the literature contains a plethora of possible assessment procedures which have been proposed to help diagnose a sacroiliac joint problem, most based on biomechanical models. Numerous studies are now available that consider whether palpation and osteokinematic testing can detect positional faults and joint movement abnormalities. Alternately, pain provocation tests have been studied both in terms of reliability and validity, but are not designed to give any biomechanical insight. Depending on their training, clinicians tend to follow one or the other of these assessment paradigms.

The last two decades have provided us with a volume of evidence that gives some clarity, though clinician friendly summaries are needed to facilitate translation of this evidence into clinical practice.

**The objectives for this session will be to:**

1. Outline and critique the literature pertaining to the three main areas of controversy noted above (prevalence rates, ROM, assessment procedures).
2. Present a systematic evidence based assessment algorithm to rule out the lumbar spine and demonstrate which tests will help the clinician make a reliable and validated SIJ diagnosis.
3. Provide suggestions for how clinicians can translate the assessment findings into a patient-centered treatment approach.

Clinicians frequently encounter patients with back pain. Since the area of the SIJ is a common site for referred pain, the clinicians' first challenge is to make a differential diagnosis in an area where symptoms overlap and false positive clinical tests can create diagnostic errors. Subjectively there are only a few historical or subjective clues to help in differential diagnosis. Therefore the clinician must adopt reliable and validated clinical tests. While researchers continue to debate whether or not clinicians can accurately differentiate between lumbar sources of pain (disc, facet, ligaments, muscle), there should no longer be controversy over our ability to distinguish lumbar versus true SIJ pain.

**Relevance to Physiotherapy Profession:** Inaccurate diagnoses and the variability in treatment options that result can lead to less than optimal outcomes, wasted healthcare resources, and patient frustration. Physical therapists can increase the accuracy of their evaluation and decrease false positive sacroiliac diagnoses. Outcomes may suffer if the sacroiliac is treated when a lumbar problem is the source of pain, and vice versa. Physical therapists will benefit from learning this evidence-based method of ruling out the lumbar spine and applying reliable tests to confirm the sacroiliac as the problem. The findings from the assessment will help guide individualized treatment options.

**Target Population:** This session will be relevant to physical therapists and researchers:

Physical therapists that regularly assess, treat, and/or direct management of patients with low back pain, sacroiliac joint area pain, and related symptoms.

Researchers who are interested in exploring differential diagnosis in the lumbar spine and looking at the implications of the assessment process on appropriate patient subgrouping

**Description of Supporting Evidence:** Numerous anatomical and biomechanical studies, reliability studies, and validation studies will be summarized in order to differentiate between proven and questionable sacroiliac joint assessment methods. There is substantial evidence to suggest that the facts lie somewhere between these two widely divergent opinions. Adding strength to this body of evidence is that the findings have been validated by several independent research groups. Attendees will be provided an email link for the references summarized.

**Description:** This session will be a lecture format, followed by presentation of a published case study which will be used to illustrate the clinical application of a structured and evidence-based assessment algorithm and clinical reasoning process for identification of sacroiliac joint pain. The case study will be used to stimulate a question and answer session.

**Conclusions:** The breadth of diverse literature on sacroiliac joint diagnosis and screening can now be streamlined into an evidence-based assessment algorithm. As clinicians put this research into practice a greater consistency between clinicians can be achieved as more dependency is placed on reliable and validated clinical tests and less on those tests that have so far failed to achieve credibility in the literature. Old controversies can be put to rest so that research can move forward with randomized controlled treatment trials for sacroiliac joint pain where subject populations are not inadvertently contaminated with lumbar spine patients and the appropriate intervention can be tailored to the appropriate subgroup. This clinician friendly presentation will provide useful tools that can be immediately implemented into clinical practice.

# Abstract: P018 – OUTCOME MEASUREMENT. PART 1: WHERE ARE WE NOW?

**Published:** 2011-07

**Authors:** AM Hoens, MD Westby, P Lawrence

**Affiliations:** * Department of Physical Therapy, University of British Columbia; † Mary Pack Arthritis Centre, Department of Physical Therapy, University of British Columbia, Vancouver; ‡ Seniors Program, Vancouver Coastal Health; North Vancouver

**Learning Objectives and Session Content:** In response to requests from participants at this team's presentation at CPA Congress 2010, this session will enhance the exploration of issues related to current practices in use of outcome measurement (OM) in total joint arthroplasty (TJA).

Physiotherapists in Canada are increasingly encouraged to extend their use of standardized outcome assessment beyond that of physical impairment (e.g., range of motion and strength) to capture patient-centred outcomes such as more complex function, activity and participation. Further, therapists are requested to use outcome measures with stronger measurement properties in order to enhance clinical decision-making and permit program evaluation and research application.

At Congress 2010 we introduced three initiatives (a chart audit, focus group and survey), facilitated by the BC Physical Therapy Knowledge Broker, which collectively explored current outcome measurement use and shared creative strategies to enhance its use along the TJA continuum (pre-operative phases through post-operative rehabilitation). Concurrently, but not presented at Congress 2010, one of the members of the team undertook an extensive Delphi survey with two Canadian-American expert panels to reach consensus on clinically important measures in the post-acute arthroplasty population. The collective results of these four initiatives are providing greater clarity as to what steps need to be undertaken to support physical therapists in their regular and sustained utilization of OM. This session will provide participants with a tangible framework for understanding the current utilization of OM and lay the foundation for the subsequent session "Outcome Measurement. Part 2: Similar to Exercise, Incorporating Simple Strategies into Your Current Way of Practice Can Lead to Sustained Use and Rewarding Benefits."

Objectives:

1. To highlight the findings of four complementary research initiatives aimed at better understanding current use of outcome measures in the total joint arthroplasty patient population.
2. To illustrate the importance of the successful partnerships, facilitated by a Knowledge Broker, incorporating multiple stakeholders' views and data gathering methods in developing a full/comprehensive picture of outcome measure use and contextual factors that may influence future use and successful knowledge transfer strategies.
3. To engage participants in a discussion on the clinical implications of the use of standardized outcome measures along the TJA continuum.

**Relevance to Physiotherapy Profession:** Total joint arthroplasty is an increasingly common surgery for advanced osteoarthritis of the hip or knee. In 2006/07, more than 62,000 procedures were performed, with more than 11,000 in BC alone. Most of these clients receive physiotherapy services in one or more clinical settings as part of their routine rehabilitation with the aim of restoring strength, mobility, physical function and quality of life. Physiotherapy interventions after these surgeries vary tremendously across Canada and world wide, as does the use of outcome measurement in this patient population (Westby 2010). Similar to other health professional organizations, the Canadian Physiotherapy Association promotes the use of standardized outcome measures in the clinical setting. This session, together with the content of a complementary session (Part 2) will provide physiotherapy leaders and frontline clinicians with the knowledge and tools to use OM to enhance clinical decision-making and program evaluation ultimately improving patient-centered care.

**Target Population:** This session will be of interest to clinicians, educators, knowledge brokers, researchers and decision makers interested in outcome measurement in, but not restricted to, the total joint arthroplasty population. Indeed, the concepts and principles will be relevant across the spectrum of practice areas.

**Description of Supporting Evidence:** Use of outcome measurement in joint arthroplasty rehabilitation research is inconsistent making clinical interpretation of treatment effects problematic (Riddle 2008). This is even more evident in joint arthroplasty clinical practice (Westby 2010) and in physiotherapy practice in general (Jette 2009). Barriers to routine use of outcome measures are multi-faceted and include personal (practitioner) and environmental factors such as access to resources, organizational policies and the practice context (Rivard 2010). The Physical Therapy Knowledge Broker position is ideally suited to promote the use of outcome measurement through their sensitivity to the practice context and organizational factors (Rivard 2010).

**Description:** This session will consist of presentations followed by participant discussion regarding their experiences with and insight into the use of outcome measures in the clinical setting.

**Conclusions:** Understanding current practices and other contextual factors affecting use of outcome measurement provides a baseline against which the success of knowledge brokering, behavioural change and knowledge transfer strategies can be evaluated. The total joint arthroplasty population provides a relevant and important area of practice to explore these issues. This patient population has been targeted by a number of health care organizations as a key area for standardized use of outcome measurement. Multiple data collection approaches across different health care settings and with varied stakeholder input increases the likelihood of a valid and realistic understanding of “current practice” as well as an appreciation of the contextual issues and eventual success of a change in professional behaviour. The physical therapy knowledge broker is well positioned to facilitate the steps required to elicit this change.

## References

[1] Westby MD, Backman CL (2010). Patients and health professional views on rehabilitation practices and outcomes following total hip and knee arthroplasty for osteoarthritis: a focus group study. BMC Health Services Research.

[2] Riddle DL, Stratford PW, Bowman DH (2008). Findings of extensive variation in the types of outcome measures used in hip and knee replacement clinical trials: A systematic review. Arthritis Rheum.

[3] Jette DU, Halbert J, Iverson C, Miceli E, Shah P (2009). Use of standardized outcome measures in physical therapist practice: perceptions and applications. Phys Ther.

[4] Rivard LM, Russell DJ, Roxborough L (2010). Promoting the use of measurement tools in practice: A mixed-methods study of the activities and experiences of physical therapist knowledge brokers. Phys Ther.

# Abstract: P019 – OUTCOME MEASUREMENT. PART 2: SIMILAR TO EXERCISE, INCORPORATING SIMPLE STRATEGIES INTO YOUR CURRENT WAY OF PRACTICE CAN LEAD TO SUSTAINED USE AND REWARDING BENEFITS

**Published:** 2011-07

**Authors:** PW Stratford, DM Kennedy, A Kozlowski, V Wong, G Alcock

**Affiliations:** School of Rehabilitation Science, McMaster University, Hamilton, ON; Sunnybrook Holland Orthopaedic & Arthritic Centre, MSK Program, Toronto; Department of Physical Therapy, University of Toronto; Rehabilitation Institute of Chicago, Centre for Rehabilitation Outcomes Research; University of Western Ontario, Fowler Kennedy Sports Medicine Clinic; London, ON

**Learning Objectives and Session Content:** Physiotherapists use impairment level outcome measures (e.g., range of motion and strength) on a daily basis to identify patient problems and evaluate change over time. An ongoing challenge is to integrate standardized outcome measures that assess functional status into the clinical decision-making and clinical reflection processes (e.g., goal setting and outcome assessment). Similar to commencing a personal exercise program, sometimes the practice challenge is working out regularly, given that there is an additional initial burden with a lag to receiving benefits. This session will show that behaviours currently used to assess impairments are transferable to the assessment of activities and participatory endeavors, and that the benefits can more than offset the expenditure required to adapt one's practice. Also like exercise, the means to which an individual clinician can be motivated to ‘get moving’ may be related to their practice style. Thus matching the approach to implementing an outcome evaluation strategy to the clinician's practice style may improve the potential for long-term adoption.

Objectives:

1. Assess practice style relative to evidence based practice and recognize how one's practice style might impact one's motivation, interest, and willingness to implement an outcome evaluation process.
2. To introduce Six Simple Strategies to ensure the Sustained Success of Standardized outcome measures in clinical practice (6 S's) (1) Start by declaring what it is you hope to assess with the measure. (2) State important patient related constraints. (3) State important constraints relevant to your clinical setting. (4) Search efficiently and effectively for relevant outcome measures –resources exist to help you. (5) Seek out measures that provide information on the interpretation of score values (6) Scrutinize the measurement properties.
3. To illustrate the importance and benefits of interpreting standardized outcome measures' scores and integrating the information into the clinical decision-making process to develop realistic goals, guide treatment and better address patients' needs. (1) How confident am I in a measured value? (2) What does the measured value mean? (3) How much change is required to be reasonably certain a patient has changed? (4) What is the ideal reassessment interval to assess the change? (5) What is the patient's expected terminal goal value? (6) When is the patient expected to reach his expected goal value?

CPA's Outcome Measurement initiative will also be highlighted as it includes the development of an online tool to improve accessibility and interpretation of outcome measures for Canadian physiotherapists.

**Relevance to Physiotherapy Profession:** An ongoing priority of the Canadian Physiotherapy Association is the successful use of standardized outcome measures to enhance clinical decision-making. Often the impetus for using outcome measures has been external (e.g., payers and administrators); this session will focus on the positive personal benefits to patients and physiotherapists gained by interpreting the scores of outcome measures.

**Target Population:** By offering key principles and strategies, the session will be relevant to all physiotherapists who assess the outcomes of patients across the spectrum of practice areas.

**Description of Supporting Evidence:** Despite the importance and rationale for outcome measurement, studies have shown that a significant number of physiotherapists still do not regularly use standardized outcome measures. In a recent survey examining the use of standardized outcome measures in physical therapy practice, only 48% of participants used them. Reasons for not using standardized outcome measures included: time for patients to complete; time for clinicians to analyze/calculate/score and difficulty for patients to complete independently. A number of other studies have cited a variety of organizational and practitioner barriers including the difficulty to critically appraise the measures and interpret their results. Understanding clinician practice traits may provide some assistance in developing implementation strategies.

**Description:** This interactive session will consist of a mix of presentations from different stakeholders (Academic, Clinical, and Professional Association) and will involve presentations and participant feedback. It will build on information shared in Outcome Measurement. Part 1: Where are we now?

**Conclusions:** Assessing patients and applying the information gained to guide clinical decisions forms the cornerstone of physiotherapy practice. Confidence in clinical decisions is directly related to the confidence in measured values on which the clinical decisions are based. The hallmark of standardized outcome measures is their detailed description for administration and scoring, as well as information concerning the interpretation of score values, and the extent to which the measures have been validated in a clearly described context. It is our belief that the primary reason for the less than optimal utilization of standardized outcome measures is the lack of understanding of the benefits to the patients and physiotherapists. The information provided in this session will be useful in conveying the benefits to patients and physiotherapists, and in providing strategies for selecting the most appropriate measure given the constraints unique to each physiotherapist's practice.

# Abstract: P020 – CLOSING THE GAPS IN KNOWLEDGE AND CARE FOR PEOPLE WITH EATING DISORDERS: AN EMERGING ROLE FOR CANADIAN PHYSIOTHERAPISTS

**Published:** 2011-07

**Learning Objectives and Session Content:** Excessive exercise can be viewed as the alpha and omega of the eating disorder (ED). It is both a common trigger in ED development and the last symptom to resolve, occurring in persons with anorexic and bulimic-type disorders, with prevalence between 30-80% (Meyer, Taranis and Touyz, 2008). For people with an ED, excessive exercise behavior leads to longer bouts of hospitalization, predicts shorter relapse time post-discharge and points to a poorer longer term outcome generally. The Readiness and Motivation Interview (RMI) can measure where a person is in regard to their stage of change with this behavior. Despite all the evidence, there are currently no specific guidelines regarding the targeting of exercise or excessive activity within therapeutic interventions for eating disorders (Meyer 2008).

Physiotherapists are the experts in exercise metabolism, training adaption, sports biomechanics and exercise prescription, as well as assessment and intervention of orthopedic and athletic injuries. This session will explore how physiotherapy knowledge and skill is essential in an interdisciplinary approach to the treatment of people with eating disorders.

Learning Objectives:

1. To understand the clinical difference between excessive exercise and overtraining in physically active women.
2. To learn the physical complications of an Eating Disorder and how they apply to exercise prescription and treatment options for physiotherapists.
3. To use two case studies to explore how using an innovative motivational, multi disciplinary approach can effectively address excessive exercise.

**Relevance to Physiotherapy Profession:** In Austria, Switzerland, Sweden and Australia, physiotherapy is already an integral part of the treatment of people with EDs. A role for Canadian Physiotherapists is now emerging, but progress is slow. While multidisciplinary programs exist across Canada to help clients manage eating disorders, the HOPE (Healthy Opportunities for People with Eating Disorders) program in St. John's, Newfoundland is the only Public Health Program in the country to employ a physiotherapist solely to work with people with a diagnosis of an ED.

Eating disorders are a mental illness with significant physical complications. A variety of physical impairments are under-recognized in this population, including musculoskeletal complications such as low bone density, proximal muscle weakness, muscle and tendon shortening, tendonitis, stress fractures, poor posture and spinal alignment.(Michelle & Crow,2006: Katzman, 2005). Physiotherapists have the expertise to treat these complications, but there is a danger inherent in treating clients with these issues without understanding the underlying pathology.

Over training and excessive exercise lead to an energy imbalance which can be life threatening: arrhythmias or cardiac failure due to dehydration, hypophosphatemia, hypokalemia and hypomagnesium. In treating these serious physical symptoms it is easy to loose sight of the fact that excessive exercise has its roots in mental health disorders, eating disorders in particular. Anorexia has the highest mortality rate of all the mental health disorders. People diagnosed with AN have a 57% increased likelihood to die from suicide than people without (Keel PK, Dorter, Eddy, Franco, Charlatan & Herzog. 2003). Physiotherapists can aid in the detecting the early signs of an eating disorder. Early detection and intervention can decrease the likelihood of such tragedies.

Mental illness can lead to physical distress, but the current health care model is one that continues to separate mental and physical health to the detriment of the client. As physiotherapists become more involved with the treatment of EDs recognition of potential mental illness is crucial. We need to be aware of the red flags in order to know when the expertise of other professionals is required. This is critical to ensure that comprehensive care is provided.

**Target Population:** This presentation is relevant for physiotherapists working with persons with eating disorders or those at risk for eating disorders (competitive athletes, physically active woman, women in the Military and children and adolescents). Issues raised will also be important for therapists working with clients in other areas of mental health, with altered bone metabolism and in other areas using a motivational approach.

**Description of Supporting Evidence:** Currently, there are no specific guidelines regarding the targeting of exercise or excessive activity within therapeutic interventions for eating disorders (Meyer 2008). This is primarily due to the common belief that exercise hinders weight gain (Calegero & Pedretty, 2005) and the lack of formal exercise protocols. It is recognized that any exercise program with this population needs to be supervised by a trained professional (Thein et al, 2000). Calegero and Pedrotty (2004) report the use of an exercise program that targets excessive exercise in women with eating disorders results in positive change without interfering with weight gain. In less than 4 weeks, women in the exercise program reported reduced disorder of thought, feeling and behaviors about exercise. Ron Manely and Kit Stanish (2003) report that as the result of an in-patient supervised exercise group, 33% their clients reported their obsession with exercise had decreased. An equal amount reported improved body image; 63% indicated the group increased their interest in pursuing exercise for fun and 90% said that an exercise group should be offered as part of their treatment.

A motivational approach incorporates empathy and reflective listening to promote an open discussion about change (Miller & Rollnick, 2002).It is a useful assessment and intervention strategy in the management of a wide range of issues including eating disorders, alcohol abuse, drug addiction, obesity and physical inactivity. Using the motivational approach, Jossie Geller et al (2008) devised a Readiness and Motivation Interview (RMI) to assess the readiness for change in people with eating disorders. The RMI is a symptom specific measure of readiness for change. Unlike global measures of readiness, which conceptualize the disorder as a single entity, the RMI provides readiness and internality (the extent to which change is occurring for self versus others) scores for each symptom domain of an eating disorder. As a result, a clinical team can know where a client is with regard to their readiness to change (pre contemplation, contemplation, preparation, action and maintenance) behaviors such as restricting, binge/purge and exercise. Knowing a client's stage of change with regard to exercise is valuable information and can assist a physiotherapist in designing effective therapeutic exercise and treatment for this population.

**Description:** A didactic presentation supported by clinical examples.

**Conclusions:** Research indicates that an ED reduces life expectancy by as much as 25 years and a third of people who develop an ED never recover (Guesella & Casey, 2002).

Physiotherapists need to be able to tell the difference between healthy physical activity and excessive exercise rooted in mental illness. We also need to be able to work together with our fellow health care professionals to effectively provide intervention when we see that line has been crossed.

The knowledge gap on how to address exercise and the physical complications of an eating disorder is as large as the divide between physical and mental healthcare models. This presentation is the initial step toward exploring best-practice to closing these gaps. Only then can we achieve our ultimate goal of putting our clients on the path to true wellness.

# Abstract: P021 – SPORT SPECIFIC PROGRAMMING IN REHABILITATION AND EVIDENCE OF BEST PRACTICE: EVALUATION AND INTERVENTION OF THERAPEUTIC GOLF REHABILITATION

**Published:** 2011-07

**Authors:** S Shatil, L Golverk

**Affiliations:** Neuphysio Rehabilitation, London, ON

**Learning Objectives and Session Content:** 1) To review the evidence supporting Therapeutic Golf Rehabilitation as a clinical physiotherapy program and business model. 2) To describe the treatment approach and program components through interactive case studies. 3) To present a framework of translating innovative programs into business practice methodology and promote discussion around same.

**Relevance to Physiotherapy Profession:** Physiotherapists are the ideal professional to assist people to golf after injury or disease. Therapeutic Golf Rehabilitation involves the use of golf as a tool to improve balance and return to sport. By integrating golf training principals with physiotherapy and practice, performance is enhanced both on and off the course. Sport specific programs are a valuable addition to the rehabilitation process, are easy to implement and create satisfied patients.

**Target Population:** This session will be of interest to physiotherapy clinicians, entrepreneurs, managers, researchers and golfers.

**Description of Supporting Evidence:** Participation in sport and leisure activities promotes health through the lifespan and is recommended for all persons regardless of ability. According to statistics from The Heart and Stroke Foundation and Canadian Stroke Network (2009), about 300,000 Canadians are living with the effects of stroke. Based on participation, Statistics Canada (2008) reports golf as the number one recreational activity in Canada with an estimated 6 million golfers and an expected growth rate of 14 percent between 2009 and 2011. People living with stroke can use golf as a means to promote health. According to the literature, there is evidence that golf increases flexibility, improves core and extremity strength, balance, postural control and coordination, speed and functional fitness in a healthy population. Older golfers score higher on balance tests than non-golfers.

Therapeutic Golf Rehabilitation was first described in the literature in 2001. It is based on sound principals of rehabilitation including neuroplasticity, motor learning, practice and performance. Participation has been shown to improve balance and quality of life in stroke survivors. Three single case study designs demonstrate the philosophy, treatment techniques and outcomes of Therapeutic Golf Rehabilitation. A business model based on five years of operations projects early return on investment and satisfied customers.

**Description:** This session will be interactive lecture format, with opportunity for discussion regarding clinical experiences and entrepreneurial ideas. Power point, a laptop, a projector, a screen, and podium microphone will be required.

**Conclusions:** Using sport during rehabilitation is valuable to the physiotherapy process. Therapeutic Golf Rehabilitation is one example of a successful sport specific physiotherapy model that is easy to implement, enhances outcomes and brings joy to patients.

# Abstract: P022 – EUCAPNIC BREATH RE-TRAINING AS A PAIN MANAGEMENT TOOL

**Published:** 2011-07

**Authors:** A Heayn

**Affiliations:** Body Help Wellness Education

**Learning Objectives and Session Content:** At the end of this workshop participants will learn:

- the relation between pain control and the physiology of Hyperventilation Syndrome
- basic techniques to improve patient CO2 levels
- a simple equipment–free CO2 monitoring process

During this 2-hour workshop, exercises will be combined with lectures regarding the theory, research and physiology concerning eucapnic breathing, hyperventilation syndrome and client pain experience

**Relevance to Physiotherapy Profession:** Physical Therapists are 'drug-free-practitioners' developing and directing therapeutic exercise, and advocating health programs to treat, reverse and / or prevent pain and dysfunction for our clients. Since the pharmaceutical revolution of the 1940's, many conditions that could be treated by physical therapists have been eclipsed by drug intervention (Field, 1998).

'Mindfulness breathing' has been shown to beneficially modify response to pain stimuli (Zautra et al 2010), and eucapnic breathing techniques have been shown to have substantial benefit in other hyperventilation related conditions such as asthma, with up to 96% reduction in medication usage (Cowie et al, 2008; McHugh et al, 2003).

While this workshop specifically addresses eucapnic breathing as a pain management tool there is also significant research demonstrating its value for management of asthma and other hyperventilation related disorders, and as a tool for improving fitness and sport performance.

**Target Population:** PT's working with chronic pain populations

**Description of Supporting Evidence:** There is a subgroup of often difficult to manage patients suffering from various conditions clinically linked to chronic hyperventilation such as: hyperalgesia, persistent neural sensitization, sweaty or tingling hands and feet, migraines, headaches, dizziness, non-specific abdominal pain, and anxiety. And unrecognized hypocapnia is common in fibromyalgia, Chronic Fatigue Syndrome, and nonspecific dizziness (Naschitz JE, et al 2006). Hyperventilation in its various forms is known to effect neural sensitization and provoke 'strange' and seemingly unrelated symptoms (Gardner 1996, McLaughlin 2009). Claims have been made that more than 60% of ambulance calls in the USA are related to hyperventilation dysfunction whether chronic, sub-acute or acute (Litchfield 2003).

Estimates of disordered breathing problems in the general population vary from 10% to 90%. A 2004 pilot study by Perri and Halford determined a figure of 75% leading them to suggest that of any four new patients presenting on any given day, the condition of three of them will be affected by breathing issues.

**Description:** Exercise instruction will be integrated with lecture material incorporating a review of the theory, research, and physiology informing this approach to pain management. A self-monitoring process will be repeated throughout the session so that participants can monitor their own changes in capnic status during the workshop. The basic capnic modification exercise, with variations, will be taught and contextualized. There will be a question period and discussion at the end.

**Conclusions:** Aside from the cost of pain syndromes in human suffering, the economic burden of chronic pain in Canada, including medical expenses, lost income, and lost productivity, is estimated to exceed $12.5 billion annually (Warriner B et al 2007) and medications often carry with them unintended adverse effects.

The value of offering clients improved self-control over pain while possibly also effecting concurrent reduction in potentially dangerous drug side-effects seems self-evident, as is the potential reduction in treatment costs to the individual and to society.

# Abstract: P023 – EXPLORING THE ROLE OF PHYSIOTHERAPISTS IN ADVOCATING FOR EQUITABLE HEALTH CARE FOR ABORIGINAL PEOPLE IN CANADA

**Published:** 2011-07

**Authors:** D Levac, D Taylor, R Roots, KJE Reinikka, M Fricke, T Miller-Mifflin, L Arcobelli, E Foster-Seargeant, D Debassige

**Affiliations:** * School of Rehabilitation Science, McMaster University, Hamilton, ON; † St. Joseph's Care Group, Thunder Bay, ON; ‡ University of British Columbia, Vancouver, BC; § Northern Ontario School of Medicine, Thunder Bay, ON; ¶ School of Medical Rehabilitation, University of Manitoba, Winnipeg, MB; ** Department of Health & Social Services, Iqaluit, NU; †† Cree Board of Health and Social Services of James Bay, Chisasibi, QC; ‡‡ Manitoulin Physio Centre

**Learning Objectives and Session Content:** Upon completion of this presentation, participants will:

1. Understand the health disparities experienced by Aboriginal people using the Social Determinants of Health framework.
2. Appreciate how the Canadian health system organization and administration impacts upon Aboriginal health.
3. Link the concept of advocacy to the physiotherapy profession and to CPA position statements on health determinants, population health, and primary health care.
4. Develop strategies for how, as physiotherapists, we can identify opportunities to advocate for improving the health of Aboriginal and other marginalized populations in Canada.

Participation in international health initiatives is of interest to many Canadian physiotherapists. However, practical constraints prevent most from traveling abroad to volunteer their skills with marginalized populations in low and middle income countries (LMICs). Socioeconomic disparities faced by marginalized populations in Canada are often similar to those of LMICs. In particular, Aboriginal peoples have well-documented health inequities arising from a history of colonization impacting social and economic determinants of health. Diverse ethnicities, differing provider jurisdictions and governing authorities, and varying levels of governance and stability across communities further complicate these issues. Addressing these inequities offers an opportunity for physiotherapists to become involved in ‘closer-to-home’ health equity initiatives. Physiotherapists interested in these issues are supported by relevant CPA position statements outlining the role for physiotherapy in advocating for and addressing population health issues within Canadian clinical practice. However, the role of physiotherapists in creating and implementing advocacy efforts regarding these important issues remains largely unexplored.

The purpose of this session is to illustrate how the physiotherapy scope of practice can address health determinants and advocacy for individuals and community health, with a focus on Aboriginal peoples. The panel will provide examples of advocacy on three levels: individual, community, and provincial/federal. At the level of the individual, these may include how physiotherapists can advocate for individual health care needs and access to services, including culturally relevant services. The role of a physiotherapist within the community health services model will be explored, including disease prevention, health promotion and education, community capacity building, and epidemiological monitoring. Physiotherapists are also advocates at the provincial and federal level through policy development, agency partnerships, curriculum development, continuing education programs for health care professions (including physiotherapy) on cultural relevance and competence to work with Aboriginal cultures, as well as capacity-building initiatives for Aboriginal physiotherapists. Finally, physiotherapists can engage the public in raising awareness and understanding of Aboriginal health, culture, and their social determinants of health. The presentation will discuss these examples and associated challenges; providing strategies for supporting physiotherapist involvement in these issues.

**Relevance to Physiotherapy Profession:** Aboriginal peoples make up a significant percentage of the physiotherapy client population in both rural and urban settings across Canada. CPA position statements encourage physiotherapists to be advocates for the health, mobility, and independence of all Canadians and to consider political, ethical, and social issues impacting patient welfare within their practice. Advocacy for health equity, whether on the level of individual clients, communities, or policies, is an emerging role for physiotherapists about which little information exists. A better understanding of the determinants of health of Aboriginal Canadians and this role will provide physiotherapists with an opportunity to address these issues and has the potential to improve the health of marginalized populations in Canada.

**Target Population:** The content of this presentation will appeal to educators, policy-makers, physiotherapy students, physiotherapists, and physiotherapy support workers at all levels of their careers, who have an interest in developing and implementing advocacy efforts for marginalized populations in Canada and internationally.

**Description of Supporting Evidence:** People of Aboriginal ancestry represent a growing segment of the Canadian population (Department of Indian and Northern Affairs, 2010). Aboriginal peoples in Canada experience lower life expectancy, higher incidence of chronic conditions and infectious diseases such as diabetes, obesity, and tuberculosis (Sarkar, Lix, Bruce, & Young, 2010), and greater rates of smoking, suicide, violence, infant mortality, and substance use as compared to non-Aboriginals (Frohlich, 2006). Social and economic factors are key determinants of health in this population, particularly: poverty and unemployment, inadequate housing, and limited access to education, health care services, and food security. Many feel that these factors have their roots in a history of colonialism, oppression, discrimination, and assimilation, as seen most dramatically through the multi-generational impact of the residential school system (Health Council of Canada, 2005).

These matters are further complicated by differing provider jurisdictions and governing authorities for health and social programs (federal, provincial/territorial, and Aboriginal), diverse cultures, ethnicities (e.g. First Nations people with or without registered treaty status, Inuit, and Métis), histories, geographies, varying levels of stable governance and communities facing a variety of crisis level health and social issues. Finally, physiotherapists must consider the ethical challenges of potential advocacy efforts in the context of historical injustices faced by Aboriginal communities for whom policies have traditionally been imposed with little stakeholder input.

The CPA advocates for the health, mobility and independence of all Canadians. The position statements on the determinants of health and population health suggest that the profession has a role in advocating for healthy public policies (CPA, 2010). Physiotherapists are able to address the determinants of health in their daily professional practice and are competent in identifying risk factors for disease and disability in individuals and their environment. Therefore, physiotherapists can, and should integrate a population health approach in their practice by collaborating to develop interventions that address population health needs (CPA, 2010).

However, there is little information available to guide the specifics of how physiotherapists can become involved in advocacy efforts on any level regarding Aboriginal health in Canada. A review of research exploring the health needs of Aboriginal populations in Canada did not include papers that were rehabilitation-specific (Young, 2003). Further education and input in the form of practical, grass-roots initiatives will assist physiotherapists to play a greater role in health promotion and prevention and advocate for improving health equity.

**Description:** This will be a structured panel discussion in which three experts will each present their experience and insights related to the content areas. A moderator will facilitate a discussion with the audience to explore the advocacy role of physiotherapists.

**Conclusions:** The International Health Division aims to promote advocacy in global health and human rights issues in Canada and abroad. Health equity for Aboriginal peoples in Canada is a substantial social issue that can be addressed by physiotherapists working in rural and urban settings across Canada. This session will provide physiotherapists with the tools to advocate for and address typical ‘international health’ issues within their practice in Canada. The session will provide opportunities for participants to learn from the panelists and from each other by providing a forum for discussion and networking.

# Abstract: P024 – LOW BACK PAIN: WHAT'S THE NEXT PRIORITY AFTER RULING OUT RED FLAGS? A MOVE TOWARDS STANDARDIZING AN ASSESSMENT AND TREATMENT PATHWAY

**Published:** 2011-07

**Authors:** A Long, R Rosedale, C Davies

**Affiliations:** * Bonavista Physical Therapy, Calgary, AB; † London Health Sciences Centre, London, ON; ‡ Westminster Physiotherapy Clinic, New Westminster, BC

**Learning Objectives and Session Content:** In low back pain research there has been repeated calls for the identification of specific subgroups that can be matched to specific interventions. This has contributed to an increased focus on a growing number of classification systems that have developed in order to address this need. The systems derive from a number of physiotherapeutic approaches and have significant variability in the assessment procedures used and in the subgroups identified. However, there is a degree of overlap between a number of these systems. This overlap involves the relatively large subgroup of patients found to have Directional Preference and/or the Centralization Phenomenon described by Robin McKenzie and originally used exclusively within the context of the McKenzie System of Mechanical Diagnosis and Therapy.

The objectives for this session will be to:

1. Explain how using a reliable assessment to identify a large subgroup improves the efficiency of the overall patient care pathway.
2. Provide a summary of over 90 clinical publications documenting directional preference and the centralization phenomenon.
3. Summarize and critique prevalence rates and discuss how this important body of literature can be used to promote the role of physiotherapy in the primary care of low back pain, with or without sciatica.

The initial patient contact is crucial and there is a strong consensus in the literature and guidelines that screening for red flags is the first priority. After this step, there is very little consensus on what type of screening or form of assessment should follow. The result of this lack of consensus is that the patient's experience can be extremely divergent. It will vary greatly depending on the practitioner's choice of treatment philosophy, style of practice and form of training.

A large body of evidence will be used to make a case for the assessment for directional preference and centralization as the next important step after the exclusion of red flags. Assessment methods employed to rule in or rule out these phenomena create a clear path to identifying the subgroup of patients that respond to a simple exercise based intervention with subsequent rapid resolution of pain and restoration of range of movement. If the potential for a simple solution is missed at this early stage, more burdensome, complex, invasive and costly interventions may needlessly be pursued. The implications are that patient satisfaction, outcomes, and potential health care costs will all be adversely impacted.

The exponential growth in the evidence-base supporting mechanical assessment and classification of LBP is challenging for working clinicians to track. Cook (2008) has proposed that synopses of literature will become one method of providing clinicians with up-to-date evidence summarizing large volumes of literature which the average clinician does not have time to search, read, and interpret. The content of this session will be focused on putting into context over ninety clinical articles related to DP and CP. Trends emerging from the body of evidence will be outlined, strengths and limitations will be discussed, and future directions will be proposed. The assessment method needed to identify this important subgroup is used worldwide, and in some countries it is the predominant form of spinal assessment. It is not however so commonly practiced in Canada and this summary of the literature may be the first step that informs and inspires clinicians to translate this evidence into clinical practice.

**Relevance to Physiotherapy Profession:** Physical therapists have the skills to transform the primary care of low back pain and sciatica. Adopting assessment methods that reliably classify low back pain patients into treatment and prognostic subgroups can reduce waste of healthcare funds and help direct resources where they are most needed. An understanding of this specific screening procedure and of the implications of the research could lead to its inclusion as an important component in promoting the profession's potential role in musculoskeletal care.

Emerging trends in continuing education have resulted in clinicians acquiring a large repertoire of tools for their treatment tool box. More than ever clinicians need to reflect on their choice of an assessment tool box in which to organize these many treatment options. Assessing the options in a way that reflects the strength of supporting literature will enhance the clinician's ability to prioritize and focus on the components of the assessment which reliably direct treatment. This will facilitate the early identification of a subgroup of patients with a good prognosis and predictable response to simple interventions.

**Target Population:** This session will be relevant to physical therapists that regularly assess, treat, and/or direct management of patients with LBP with or without sciatica. The content is applicable to acute or chronic patients, and pre-surgical decision making.

**Description of Supporting Evidence:** Over 90 clinical publications will be grouped and the resulting trends summarized. These references include 17 reliability studies, 50 clinical studies including a variety of observational, case series, prognostic studies, and diagnostic validation studies, and 29 randomized controlled trials pertaining to directional preference and centralization. Participants will be given an email link for the references summarized. Strengths and limitations will be discussed.

**Description:** This session will be a lecture format, followed by a general question and answer session and brainstorming regarding barriers to translation of this body of evidence into clinical practice.

**Conclusions:** Centralization and Direction Preference are well documented in the literature and represent the largest and most validated subgroup. This summary of this fast growing body of evidence will help clinicians gain insight into method that can improve traditional care pathways for low back pain and/or sciatica, and promote our profession as primary care providers

# Abstract: P025 – SOUNDER SLEEP TO UNRAVEL THE INSOMNIA-PAIN CYCLE

**Published:** 2011-07

**Authors:** A Heayn

**Affiliations:** BodyHelp Wellness Education

**Learning Objectives and Session Content:** At the end of this workshop participants will learn how to:

- Reduce their own stress and improve their sleep
- Assist their clients with sleep and stress disturbances
- Ease pain syndromes to facilitate client healing.

During this experiential 'sleep-shop' exercises will be interspersed with lectures regarding the theory, research and physiology of sleep, sleep disturbance and client pain experience.

**Relevance to Physiotherapy Profession:** Physical Therapists are 'drug-free- practitioners developing and directing therapeutic exercise, and advocating health programs to treat, reverse and / or prevent pain and dysfunction for our clients. Since the pharmaceutical revolution of the 1940's, many conditions that might have been treated by physical therapists have been eclipsed by drug intervention (Field, 1998). Research clearly indicates the negative effect of disturbed sleep in decreasing pain threshold (Calli-Schmidt 2003; Edmounds, 2009; Hakki Onnen 2001; Older 1998). In addition to pain, many of our patients are dealing with trauma-related stress following motor vehicle accidents. Moreover, other research demonstrates linkages between sleep, healing and other health issues such as obesity, diabetes, memory loss, and psychological disturbances (Barclay L 2010; Mednick S 2006). This workshop specifically addresses improving sleep quality as a stress-reducer and a pain management tool.

**Target Population:** PT's working with chronic pain populations.

**Description of Supporting Evidence:** The benefits of sleep go far beyond pain control. Sleep is sufficiently vital to health that in the natural world some animals actually fly or swim with one half of their brain awake while sleeping with the other half (Rattenborg NC et al 2000) and sleep deprivation is a common form of torture (West LJ et al 2006).

Various sources indicate a prevalence of sleep disturbances in the general population of 25–30 % regularly to up to 70% occasionally. There is evidence that pain and sleep disturbances have a 'bidirectional relationship' with up to 88% of patients with chronic pain also demonstrating sleep disturbances. Decreased sleep has a hyperalgesic effect and significantly decreases pain thresholds (Webster LR et al 2008). Furthermore problems with sleep are a significant predictor of low back pain in industrial workers. (Miranda H et al 2008)

Selye's work on the General Adaptation (Stress) Syndrome tells us that stress, the body's response to stressors, is cumulative if not somehow relieved. Research on 'whiplash victims' shows stress, pain and insomnia to be correlated factors in maintaining disability (Selye 1974; Scaer 2001). The exercises as instructed in this workshop are intended to interrupt this stress accumulation model.

**Description:** There are two inter-locking parts to this session. First, it is intended to be significantly experiential providing stress-reduction benefit to the participants. Secondly, the experience / instructions in the neurosomatic focusing exercises are intended to help overcome the 'cognitive popcorn' that keeps us awake. Therefore, exercises will be integrated with lecture material to incorporate a review of sleep theory, sleep research, and physiology. This approach to pain management allows participants to use these techniques with their own clients. There will be a question period and discussion at the end.

**Conclusions:** Aside from the cost of pain syndromes in human suffering, the economic burden of chronic pain in Canada, including medical expenses, lost income, and lost productivity, is estimated to exceed $12.5 billion annually (Warriner B et al 2007). Moreover, sleep and pain medications often carry with them unintended adverse effects.

The value of offering clients improved self-control of pain while possibly also effecting concurrent reduction in potentially dangerous drug side-effects seems self-evident, as is the potential reduction in treatment costs to the individual and to society.

# Abstract: D001 – MECHANOTHERAPY; THE SCIENTIFIC UNDERPINNING OF PHYSIOTHERAPY AND ITS CLINICAL IMPLICATIONS

**Published:** 2011-07

**Authors:** K Khan

**Affiliations:** University of British Columbia, Canada

**Background:** The term “mechanotherapy” refers to the process of how load may be used therapeutically to stimulate tissue repair and remodeling in tendon, muscle, cartilage and bone. Common problems such as tendinopathies, muscle tears, and fractures require controlled loading to institute healing and return to function. There has been a significant amount of literature demonstrating the benefits of exercise for patients with a wide range of musculoskeletal disorders. The scientific underpinning of how this occurs is related to “mechanotransduction”, which refers to a physiological process where cells sense and respond to mechanical loads. This session will illustrate this physiological process and provide clinical examples that turn movement into tissue healing.

**Relevance to Physiotherapy Practice:** Tendinopathies, muscle tears, and fractures comprise a significant proportion of patient problems presenting to a physiotherapists practice. Exercise therapy as well as various manual therapies apply load to tissues with the goal of promoting healing. The underlying processes surrounding the effects of these therapies are poorly understood for many clinicians. Understanding the underlying physiological process behind many of our common therapies is necessary for treatment planning, therapist and patient education.

**Target Audience:** This session is designed for physiotherapists, exercise physiologists, kinesiologists, and support workers working with patients with disorders of muscle, tendon, cartilage, or bone.

**Summary of Supporting Evidence:** Numerous systematic reviews and randomized controlled trials state that a variety of exercises provide benefit to patients with a wide range of musculoskeletal problems. Evidence has shown that tendons experience tensile and compressive forces during many functional activities, such as walking, and that these forces foster a deformation of the cell that can trigger a range of responses depending on the type, magnitude and duration of loading. Injured structures have been shown to respond favourably to controlled loading in many circumstances. Research in this area is ongoing.

**Description of Session Format:** One hour lecture with 15 minutes of audience questions.

**Session Objectives:** Upon completion of this session participants will be able to:

1. Apply the scientific basis of mechanotherapy to common patient problems.
2. Provide therapies that involve tissue loading to promote healing of many injuries presenting to a therapists practice.
3. Understand the physiological processes underlying many of the common exercises and manual therapies prescribed in a therapists practice.

# Abstract: D002 – BIO? PSYCHO? SOCIAL? – THE WAD DILEMMA

**Published:** 2011-07

**Authors:** J Elliott, M Sterling, M Sullivan

**Affiliations:** * Northwestern University, USA; † University of Queensland, Australia; ‡ McGill University, Canada

**Background:** Patients presenting with whiplash-associated disorders are a common entity in musculoskeletal clinical practice. Such presentations can be very complex in nature and require accurate diagnosis and a comprehensive bio-psycho-social approach to management. In recent years the evidence for the physical and psychological manifestations of WAD has significantly increased. These include sensory disturbances indicative of augmented central nociceptive processing, motor and sensori-motor changes. In the psychological domain, posttraumatic stress symptoms play an important role. It is important that physiotherapists can assess, interpret assessment findings and integrate these to the most optimal management approaches for WAD. Recent MRI studies have allowed further information that will assist in the diagnosis of WAD patients.

It is clear that current treatment strategies are not successful in reducing the transition to chronicity following injury. Why is this case? Are new MRI findings the reason? Are there underlying psychological mechanisms or sociological reasons not being addressed by current treatment strategies? Are new treatment approaches required? The aim of this presentation is to equip physiotherapists with a new understanding of the WAD condition that includes all aspects of this condition. Participants will learn how to integrate this knowledge into more targeted treatment approaches that go beyond the standard motor control approaches to this condition. Particular emphasis will be placed on psychosocial techniques that physiotherapists can deliver in their practice.

**Relevance to Physiotherapy Practice:** Whiplash is a recalcitrant condition to standard physiotherapy approaches to treatment. It is important that physiotherapists can recognize those patients at risk of poor recovery and/or at risk of non-responsiveness to treatment. The research evidence also suggests that some patients, especially those with poor outcomes demonstrate a complex clinical picture that will likely require an integrated approach to management from several providers. The physiotherapist, by virtue of our assessment skills, will play a unique and important role in the integration of patient care.

This presentation will provide physiotherapists with an introduction to the skills required to assess the patient with whiplash, taking into account all aspects of the condition both physical and psychological. This lays the foundation for enhanced management and improved patient outcomes.

**Target Audience:** This presentation is aimed at graduate physiotherapists who are involved in the management of patients with WAD.

**Summary of Supporting Evidence:** It is becoming clear that whiplash is a heterogeneous condition with sub-groups of patients able to be identified based on varying physical and psychological presentations (Sterling and Kenardy, 2008). Whilst the presence of motor dysfunction occurs almost universally in all those with neck pain (Jull et al., 2004; Sterling et al., 2003b), sensory disturbances and psychological factors differentiate those with higher levels of pain and disability (Sterling et al., 2010; Sterling et al., 2003a). These whiplash injured people have a more complex presentation involving widespread sensory mechanical and thermal hyperalgesia, occurring both local and remote to the site of injury, and symptoms of posttraumatic stress. Both these factors are strong predictors of poor functional recovery following whiplash injury (Sterling et al., 2006; Sterling et al., 2005). Sensory hypersensitivity has been shown to occur independently of psychological distress and likely reflects biological phenomena involving augmented central pain processing (Sterling et al., 2008). Further investigation of these phenomena has shown that chronic WAD participants with sensory hypersensitivity also demonstrate hypoaesthesia to vibration, thermal and electrical stimuli suggesting a minor peripheral nerve injury as a possible contributor to whiplash pain (Chien et al., 2010). Furthermore the presence of some of these factors (particularly mechanical and cold allodynia) in patients with a chronic whiplash condition mitigated the successful effects of multimodal physical treatment shown in patients without these features (Jull et al., 2007). Musculoskeletal clinicians play an important role in the early assessment and management of the whiplash injured. As such the early assessment and identification of features associated with both poor and good recovery is necessary for both targeted intervention strategies and/or appropriate referral. Most of these factors have been identified in a laboratory centre and it is essential that the findings are translated into clinical practice. Research has commenced on the development of time-efficient tools for use in clinical practice and these will be discussed and demonstrated. The quest for prevention of the transition to chronicity remains with improved early diagnosis and classification.

**Description of Session Format:** 3 X 30 minute presentations with 30 minute panel discussion for audience questions

James Elliott: Ongoing pain following whiplash but no visible body damage…is it time to look under the hood?

Michele Sterling: Bio or psycho: time to stop the dualism

Michael Sullivan: Augmenting the impact of physiotherapy through the use of psychosocial techniques

**Session Objectives:** At the completion of this presentation, participants should be able to:

1. Understand the emerging use and utility of MRI in the management of individuals with chronic WAD.
2. Understand the psychological assessment of whiplash and the role physiotherapists play in this assessment
3. Integrate psychological findings into their overall management of the patient with whiplash
4. Understand the role physiotherapists play in co-ordinating care of the whiplash injured person

# Abstract: D003 – INJURY PREVENTION IN SPORT: AN EVIDENCE-BASED PERSPECTIVE ACROSS THE SPECTRUM OF RECREATIONAL PARTICIPANT THROUGH ELITE ATHLETE

**Published:** 2011-07

**Authors:** C Emery, K Khan

**Affiliations:** * University of Calgary; † Faculty of Medicine and Department of Human Kinetics, University of British Columbia, Vancouver

**Background:** This evidence-based symposium will be delivered by Dr. Carolyn Emery and Dr. Karim Khan. This lecture will provide an interdisciplinary approach to injury prevention in sport which should be a key consideration for all physiotherapists in clinical practice who work with children or adults who are sport participants or elite athletes. This presentation will provide original data and evidence from the literature related to risk factor identification and prevention strategies for acute and chronic sport injury across the lifespan.

Injury prevention in sport is a key element in the delivery of care by physiotherapists working with sport participants or athletes across the lifespan in the community. Physiotherapists are becoming more active in maximizing the prevention of injuries in sport through primary, secondary and tertiary prevention approaches. Evidence related to the identification of high risk participants in sport and prevention strategies to reduce the risk of injuries will be presented.

**Target Audience:** This session will not require prior exposure to evidence related to injury prevention in sport. The presenters will address the topic of injury prevention in sport with interdisciplinary evidence across basic science, clinical and population health perspectives.

**Summary of Supporting Evidence:** The cost of injury care to the health care system and society is enormous. For example, it is estimated that unintentional injuries cost Canadians approximately 8.7 billion dollars per year with injuries to children accounting for approximately half that total. Further, unintentional injuries result in more potential life years lost before age 70 than any other single health problem in Canada. In fact, sport and recreation is the leading cause of injury in youth. Cross-sectional survey data in Alberta estimates the rate of adolescent sport injury requiring medical attention to be 38 injuries/ 100 adolescents/ year. These injuries significantly lower the quality of life of Canadians. While physical activity prevents all-cause morbidity and mortality associated with a sedentary lifestyle, injuries can become a barrier to active living. Reduction of sport and recreation-related injury would improve quality of life through the maintenance and promotion of active living and the prevention of osteoarthritis. This is a critical issue in health care and in the promotion of health and wellness in our communities. There is a rapidly growing societal need to address sport & recreation injury and its future health impact in young age groups during the time they are most active (and when the benefit of injury prevention strategies may be the greatest). Evidence from systematic reviews, cohort studies and RCTs will be provided to support this session.

**Description of Session Format:** Lecture/ dialogue between two presenters.

**Session Objectives:** Upon completion of this session, participants will understand a recursive model which allows the physiotherapist to appropriately consider target risk factors and injury prevention approaches to minimize the risk of injury in sport and recreation; be able to provide evidence-based examples of risk factors and injury prevention approaches appropriate for recreational sport participants and elite athletes; be exposed to evidence supporting prevention strategies for both acute onset and chronic injury and be motivated to incorporate primary and secondary prevention approaches into their clinical practices in order to maximize participation in sport and recreation at all levels.

# Abstract: D004 – FIT TO PLAY – CONNECTING YOUR CORE: SMART TRAINING FOR SWINGING SPORTS

**Published:** 2011-07

**Authors:** C Petersen

**Affiliations:** City Sports and Physiotherapy Clinics, Vancouver

**Background:** This session will provide a general functional definition of core stability, describe the relevant anatomy and discuss current practices in multi- core (upper and lower) stability training and it's role in the kinetic chain in swinging sports. It will review the research surrounding the functional core and it's importance in maintaining proper alignment and control of the lumbo-pelvic and scapulothoracic regions during swinging sports. Many commonly prescribed exercises by physiotherapists are machine based and involve or isolate a single joint and only allow movement in one plane of motion without full kinetic chain involvement. Participants will be educated on connecting the core with both multidirectional upper and lower core stability training thus providing smart strategies that can be taught to clients involved in swinging sports.

**Relevance to Physiotherapy Practice:** Clinicians in sports and orthopaedic settings will very likely encounter patients who participate in swinging sports. Injuries to the lumbo-pelvic (lower core) and scapula-thoracic (upper core) are common in sports like golf, racquet sports and field sports.

The changing nature and demands of sports as well as participation by ever younger athletes may also be changing the nature of these injuries. Functional data suggests that elite adolescents possess poor proprioception, strength and agility in key spine stabilizers, including multifidus, iliopsoas and transversus abdominus (Alyas et al, 2007). Non-elite clients engaging in similar swinging activities may also have the same concerns. Recent research has demonstrated that lower extremity position influences scapular muscle recruitment and muscle balance ratios in closed kinetic chain exercises(Maenhout et al, 2009) As well, trunk and lower extremity position and movement influence scapular muscle recruitment and muscle balance ratios in open kinetic chain exercises (De Mey et al, 2010).

The challenge for busy clinicians, and thus the goal of this session, is how to effectively prescribe exercises that ensure optimal recruitment, balance, timing, deceleration control and mimic performance demands of swinging sports.

**Target Audience:** Physiotherapists who treat patients that participate in swinging and throwing sports or are themselves sport participants.

**Summary of Supporting Evidence:** The anatomical and functional core has been studied at length and has been described in literature. Physiotherapists, physicians, coaches and fitness trainers are all well aware of the importance of upper and lower core stability training to a player's overall development, performance and injury prevention (Petersen, 2009). Very few competitive players in swinging sports make it through an entire season without experiencing some form of lumbar, hip, knee, thoracic or shoulder pain associated with kinetic chain weakness and/or malalignment issues (Petersen & Nittinger, 2010). Movements in swinging sports include quick acceleration and deceleration, planting and cutting, lateral movements, twisting and sliding. These quick movements pass through many planes of motion and create rotational and torsional forces on numerous joints and muscles at the same time. If core stability is inadequate this can lead to malaligment concerns and alterations in length tension relationships of muscles. This malalignment can be exacerbated by the unilateral (one sided) nature of tennis strokes since in the modern game seventy five percent of the strokes are forehand or service motion placing abnormal rotational and deceleration stresses on the dominant side (Petersen & Nittinger, 2010).

Abnormal alignment and associated biomechanical changes can contribute to injury in players due to imbalances of muscle length and strength. The subsequent increased tissue tension can cause overuse and tissue breakdown. This malalignment puts athletes at increased risk of injury, and once injured they are likely to take longer to recover, or may even fail to do so (Schamberger, 2002). Weaknesses and imbalances of the core have been related to low back pain (Akuthoto & Nadler, 2004) and lower extremity injuries (Ireland et al, 2003).

The core muscles attach in groups forming functional slings from the hips through the lumbo-pelvic (lower core) to the scapula-thoracic (upper core) regions. Four slings of muscle systems have been described in the literature. (Vleeming et al, 1995a) (Snijders et al, 1993) These are the posterior oblique sling, the anterior oblique sling, the longitudinal sling and the lateral sling. These slings of muscles help transfer energy from the legs through the core (trunk) to the upper body and arms. This is especially important in swinging sports that involve rotation and deceleration.

The core musculature includes muscles of the trunk and pelvis that are responsible for the maintenance of stability of the spine and pelvis and help in generation and transfer of energy from the large to small body parts during many sports activities (Baechle et al, 2000) (Putnam, 2003). In tennis players, the abdominal musculature plays a significant role in trunk and core stability providing a mechanical link between the lower and upper limbs (Maquirrian et al, 2007).

It is estimated that fifty percent of the force generated in a tennis service motion starts at the ground and must be transmitted through the lower to upper core and funneled through the scapula to provide power and speed (Kibler et al, 2006). Similar demands can be hypothesized for other swinging sports. This makes it important to develop a good set of connecting your core exercises to ensure these forces are transmitted efficiently.

**Description of Session Format:** This session will combine lecture and practical/demonstrations. It will highlight a series of innovative functional multi-core stability exercises that connect your core in a sequence of exercises using a variety of equipment commonly found in the clinical environment. This is a comprehensive lecture and demonstration session that provides therapists, coaches and trainers with exercise tools they can use immediately in the clinical or training venue.

**Session Objectives:** Upon completion of this session, participants will have an increased understanding of the importance of multi-core stability in working with clients involved in swinging sports. They will be better able to prescribe exercise progressions that work the muscle slings in variety of different bridging positions. As well they will be able to confidently prescribe a variety of different functional squatting, split squat, lunge and step up combinations that can connect the upper and lower core and can be carried out in a clinical setting. Participants will leave with a better understanding of multi-core stability and a repertoire of exercises appropriate for multiple sports that can be used by athletes of all ages and can be implemented immediately.

# Abstract: D005 – CAUSE AND PREVENTION OF FALLS AND FALL-RELATED INJURIES ACROSS THE LIFESPAN

**Published:** 2011-07

**Authors:** SN Robinovitch

**Affiliations:** Department of Biomedical Physiology and Kinesiology, and School of Engineering Science Simon Fraser University

**Background:** This talk will focus on the epidemiology and biomechanics of falls and fall-related injuries across the lifespan. Comparisons will be made between the causes and consequences of falls in children, young adults, and older adults. This will lead to a discussion of approaches for assessing risk and strategies for preventing falls and their related injuries in these different populations. A review will be provided of “balance recovery” for preventing falls in the event of imbalance, and “safe landing responses” for avoiding injury in the event of a fall, and how these change with age. An emphasis on the changes for older adults will be made and how these changes influence the severity and type of injuries that lead to profound morbidity and mortality in this population. Adults 65 years of age and older are the fastest growing segment of the Canadian population and falls in older adults are a significant public health issue. It is important that the unique health care needs of older adults are met to optimize health, physical function, quality of life and independent living. The speaker will draw upon examples from laboratory experiments and an ongoing project involving video capture of real-life falls for older adults in long-term care.

**Relevance to Physiotherapy Practice:** Falls are the number one cause of unintentional injury, and have especially devastating consequences for older adults. Approximately 90% of hip fractures, 60% of head injuries, and 40% of vertebral fractures in this population are due to falls. This talk will focus on new and established approaches for assessing risk for falls, and preventing injuries in the event of a fall. Specific distinction will be drawn between the ability to maintain balance, the ability to recover balance (e.g., by stepping or grasping), and the ability to safely arrest a fall (e.g., with the upper extremity). Many key risk factors for falling; strength, balance, and gait are within the clinical domain of physiotherapy. There are effective treatments available to prevent falls or aid an older adult's return to their optimal function after a fall. Physical therapists can help with both of these approaches. Working with a physical therapist will allow older adults to stay active and remain living independently in the community. The risk of falling in older adults can be reduced when specific exercises, activities and interventions are prescribed by a physical therapist. Up to date information on fall prevention and the understanding of underlying mechanisms of falls in older adults will have direct benefit on clinical care that physiotherapists provide to clients.

**Target Audience:** This talk should be appropriate to care providers and clinician scientists having a wide range of background knowledge and experience. Some familiarity with biomechanical concepts, and the literature of falls and fractures, will be useful but is not essential.

**Summary of Supporting Evidence:** Peer-reviewed published results will be reviewed from (a) prospective studies examining risk factors for falls and fall-related injuries (e.g, Study of Osteoporotic Fractures); (b) laboratory studies on age-related changes in balance maintenance and recovery, and safe landing responses; and (c) field studies involving video capture of real-life falls.

**Description of Session Format:** This session will involve a lecture of approximately 40 minutes duration, followed by a 20 minute interactive question and answer period with the audience.

**Session Objectives:**

1. improved understanding of the cause and circumstances of falls in older adults residing in long-term care;
2. improved understanding of the role of sensorimotor, musculoskeletal, and cognitive variables in the cause of falls and fall-related injuries;
3. improved understanding of the strategies that humans rely upon to avoid injury in the event of a fall, and how these are affected by age.

# Abstract: D006 – ASSESSMENT AND PREVENTION OF HIP FRACTURES IN OLDER ADULTS: A ROUND-TABLE DISCUSSION

**Published:** 2011-07

**Authors:** SW Muir, SN Robinovitch, W-L Hamilton

**Affiliations:** * Division of Geriatric Medicine, University of Western Ontario; Parkwood Hospital, Department of Geriatric Medicine, London, ON; † Department of Biomedical Physiology and Kinesiology, and School of Engineering Science Simon Fraser University; ‡ EKM Community Health Centre, Wolfville, NS

**Background:** This talk will focus on the assessment and prevention of hip fractures among older adults. The frequency of fall-related injury is nine times higher among older adults than amongst any other age group. Over half of all older adults who fall will experience an injury, of which 5-10% will sustain a major injury such as a fracture. Research evidence on the magnitude and scope of the morbidity and mortality sequelae of a fall in this group is sobering and clearly shows the public health importance of the problem. Falls have important public health implications as they are a major cause of disability and death in older adults. Hip fractures are the second leading cause of hospitalization in people over the age of 65 years and ninety percent of hip fractures are due to falls. This talk will address three topic areas i) epidemiology, risk factors and rehabilitation strategies to prevent fall-related injuries, ii) hip protectors and flooring materials to prevent injuries and iii) implementation and treatment compliance of rehabilitation interventions.

**Relevance to Physiotherapy Practice:** Falls are the number one cause of unintentional injury, and have especially devastating consequences for older adults. This talk will focus on new and established approaches for assessing risk for falls, and preventing injuries in the event of a fall. Many key risk factors for falling; strength, balance, and gait are within the clinical domain of physiotherapy. There are effective treatments available to prevent falls or aid an older adult's return to their optimal function after a fall. Physical therapists can help with both of these approaches. Working with a physical therapist will allow older adults to stay active and remain living independently in the community. The risk of falling in older adults can be reduced when specific exercises, activities and interventions are prescribed by a physical therapist. Up to date information on fall prevention, the understanding of risk factors for falls and the successful implementation of rehabilitation treatment in older adults will have direct benefit on clinical care that physiotherapists provide to clients.

**Target Audience:** This talk should be appropriate to care providers and clinician scientists having a wide range of background knowledge and experience. Some familiarity with the literature and clinical treatment of falls and fractures in older adults will be useful but is not essential.

Peer-reviewed published results will be reviewed from (a) prospective studies examining risk factors for falls and fall-related injuries (e.g, Study of Osteoporotic Fractures); (b) laboratory studies on the use of protective equipment and materials to prevent fall-related injuries (hip protectors and flooring materials); and (c) evidence-based review of intervention strategies to prevent falls and improve compliance among clients.

**Description of Session Format:** This session will be a round table discussion involving three speakers who will each give a 10-15 minute presentation. This will be followed by a 15 minute interactive question and answer period with the audience.

**Session Objectives:**

1. Improved understanding of the cause and circumstances of hip fractures in older adults;
2. Improved understanding of the role of hip protectors and flooring to prevent injuries;
3. Improved understanding of the strategies that physiotherapists can use to improve compliance with treatment interventions.

# Abstract: D007 – PHYSICAL THERAPY FOLLOWING ENDOVASCULAR INTERVENTIONS FOR MULTIPLE SCLEROSIS

**Published:** 2011-07

**Authors:** LE Klassen

**Affiliations:** Saskatoon; Bourassa & Associates Rehabilitation Centre, Saskatoon

**Background:** The etiology of Multiple sclerosis (MS) is unknown but is currently thought to be caused by a complex interaction of environmental and predisposing genetic factors. An Italian group of researchers first presented the chronic cerebral and spinal venous insufficiency (CCSVI) hypothesis in 2009, proposing that CCSVI is prevalent in those with multiple sclerosis and is a significant factor in the development and/or progression of neuropathology in MS. This group went on to develop and use endovascular treatment procedures, involving percutaneous transluminal venous angioplasty, to address cerebral and spinal venous stenosis in MS.

Controversy surrounds the hypothesis of CCSVI as a causative factor in MS, the prevalence of CCSVI in MS, and the benefits of endovascular procedures used to treat CCSVI. There is currently limited information about the short and long term safety of the procedures being used. There is also limited evidence, and none from randomized controlled trials, of effectiveness of these procedures. The evidence currently available suggests better outcomes in those who have been recently diagnosed, those with relapsing/remitting MS, and those with mild or moderate disease presentation.

Despite safety concerns and limited evidence of effectiveness, endovascular procedures for CCSVI are being offered in a number of centers throughout the world and are being accessed by hundreds, possibly thousands, of Canadians with MS. Following intervention, some form of anti-coagulant therapy is prescribed. In addition, intensive physical therapy intervention is suggested, but written exercise guidelines and precautions are not provided.

In this session, evidence supporting the benefits of exercise interventions in MS, as well as currently recommended exercise guidelines and precautions will be reviewed. The applicability of these guidelines to individuals who have undergone endovascular procedures will be discussed within the neurorehabilitation contexts of restoration, maintenance and prevention. Additional precautions that may be needed for this particular population, as well as methods of monitoring for potential adverse events following endovascular procedures will be suggested. Selected outcome measures considered useful in evaluating the effects of endovascular procedures and the physical therapy interventions that follow will be discussed. The value of collecting qualitative data to augment quantitative data will be illustrated.

**Relevance to Physiotherapy Practice:** Physical therapists possess knowledge and skills that will be of particular value to individuals with MS who have undergone endovascular procedures. Some who seek our services may be doing so for the first time. This will allow us the opportunity to promote regular exercise as one effective tool in the ongoing management of this chronic neurological condition. The development of optimal physical therapy assessment and treatment methods to be used in conjunction with endovascular procedures will take time to determine. This education session presents suggestions for the both assessment and treatment based upon recent clinical experience and currently available evidence.

Although the Expanded Disability Status Scale (EDSS) is commonly used to rate clinical status and disease progression in MS, it is inadequate for evaluating the effects of either endovascular procedures or the physical therapy interventions that follow. The usefulness of four balance measures and two gait measures will be contrasted in the case studies presented. Critical appraisal of possible outcome measures will be useful to physical therapists involved in both clinical practice and future research.

In this session, it will be proposed that interventions following endovascular procedures should initially be impairment and task oriented, focusing upon optimizing any gains in neurological function (e.g., muscle strength) and using these gains in the practice of balance and mobility tasks. A fitness orientation, in the form of aerobic exercise training should be added once compliance with the initial program is established.

**Target Audience:** A basic understanding of the clinical presentation of MS, as would be taught in a Masters PT program, is needed for participants to benefit optimally from the session. Basic knowledge of exercise prescription is also needed.

**Summary of Supporting Evidence:** The current body of evidence supporting exercise interventions for MS used in this presentation will include a meta analysis, a Cochrane review, randomized controlled trials, as well as non randomized and non controlled trials. It should be noted that the available evidence is almost exclusively derived from the study of individuals with mild to moderate disease severity.

The effects of aerobic exercise training have been studied more extensively than the effects of resistance (i.e., strength) training. The reported benefits of low to moderate intensity aerobic training include: a small but clinically significant improvement in quality of life (meta analysis), improvements in peak and maximal oxygen consumption, and improvements in mood. The reported effects of aerobic exercise training upon both lower extremity muscle strength and activity limitations suggest limited or no benefit.

The methodological quality of most studies investigating the benefits of resistance training is low. Benefits reported include consistent findings of improvements in muscle strength, mixed findings of improvements in activity limitations, and findings from single studies of improvements in fatigue and psychological well being.

As physical therapy management following endovascular surgery is an emerging area of practice, the body of evidence briefly summarized above combined with recent clinical experience (case studies) will be used as foundations to propose methods for physical therapy management before and following endovascular procedures.

**Description of Session Format:** The session will include approximately 45 minutes of lecture and 15 minutes of discussion. It is hoped that attendees will share their experiences and their ideas for managing this emerging area of practice.

**Session Objectives:** Upon completion of this session, participants will be able to:

- Identify ‘red flags’ for adverse events that may occur as a result of endovascular procedures performed on individuals with MS.
- Critically appraise outcome measures as to usefulness in evaluating the effects of endovascular procedures and the physical therapy interventions that follow.
- Include qualitative data collection methods in the evaluation of effects of endovascular procedures and the physical therapy interventions that follow.
- Modify current MS exercise prescription guidelines and precautions for use with individuals who have undergone endovascular procedures.

# Abstract: D008 – VESTIBULODYNIA: UP-DATE ON ETIOLOGY, ASSESSMENT AND TREATMENT

**Published:** 2011-07

**Authors:** E Gentilcore-Saulnier

**Affiliations:** Queen's University, School of Rehabiliation Therapy, Kingston, ON

**Background:** Vestibulodynia is a common subtype of vulvodynia (i.e., chronic vulvar pain). The pain of PVD is described as a sharp, burning pain at the entrance of the vagina in response to pressure to the vaginal entrance (Bergeron et al, 2001a). Vestibulodynia affects 12% of pre-menopausal women in the general population (Harlow, Wise, & Stewart, 2001) and has significant negative impacts on sexual functioning, relationship adjustment, psychological well-being, and overall quality of life (Arnold et al, 2006). Currently, PVD is conceptualized from a multidimensional viewpoint in which biological, psychological, and social factors are seen as fundamental to understanding the causes, effects, and treatment (Weijmar et al, 2006), and management should be based on the most recent international guidelines (vanLankveld et al, 2010).

Gentilcore-Saulnier et al's study (2010), using electromyography, demonstrated that although women with PVD exhibited significantly more superficial PFM hypertonicity and over-reactivity to pain than non-affected women, there were less consistent findings at deeper layers – there was a lack of generalized pelvic floor over-reactivity and hypertonicity. The authors posit that the tension begins as a protective guarding response to the pain and over time this response results in the resting tone observed. In addition, Goldfinger et al (2009) showed that these heightened responses were no longer observed after women had undergone a standardized physiotherapy protocol. The authors also evaluated predictors of treatment success as well as the impact of physiotherapy on psychosexual variables in women with vestibulodynia. Similarly recent research by Thibault-Gagnon & McLean (2010), using ultrasound imaging, found shortened pelvic floor muscles in women with vestibulodynia.

Typical physiotherapy interventions include education about the role of the pelvic floor muscles, biofeedback, electrical stimulation, manual techniques, and insertion techniques (e.g., use of vaginal dilators). To date, there has been one prospective or controlled studies conducted on the effectiveness of a comprehensive physiotherapy intervention on women with vestibulodynia in treating the physiological, psychological, and sexual problems (Goldfinger et al, 2009). Their evaluation of improvement of myriad outcome variables (i.e., physical, psychophysical, psychosocial) gives support to the conceptualization of vestibulodynia from a multidimentional viewpoint and highlights the interplay between physical and mental health and led to recommendations of best practice in the field of physiotherapy.

**Relevance to Physiotherapy Practice:** There has been an increasing amount of research in the field of women's sexual pain disorders in the past 10 years, leading to major changes in classification, terminology, aetiology and thus management of those conditions, and of interest of this presentation, vestibulodynia. It is suspected that few of those advances have been translated effectively into practice. Canadian physiotherapists ought to be informed of the state-of-the-art conceptualization and approach on vestibulodynia to improve patients' outcomes. Study results will inform clinicians on the impact of a rehabilitation protocol on the physical, psychological and psychosexual outcomes of women with vestibulodynia. Conceptual models for treatment will be presented. As a result, clinicians will be able to better appraise the need for multidisciplinary management of the condition.

**Target Audience:** This session is directed towards physiotherapists with interest and-or experience in the assessment and management of sexual pain disorders in women. Basic understanding of pelvic floor anatomy is requested, which corresponds to an entry-level skill level.

Information will be drawn from recent guidelines in the management of female sexual pain disorders have been put forward by the 3rd International Consultation on Sexual Medicine, an expert committee comprised of eight researchers and clinicians (VanLankveld et al, 2010). There are yet no randomized-controlled trials assessing the effectiveness of physiotherapy for vestibulodynia, hence results will be mostly drawn from recent case-control studies (Goldfinger et al, 2009; Gentilcore-Saulnier et al., 2010, Thibault-Gagnon et al, 2010), as well as review papers, expert opinion, and previous case-control and observational studies that have shaped our current state of understanding of vestibulodynia.

**Description of Session Format:** Lecture (20 min), case study discussion (5 min), question and answer period (5 min)

**Session Objectives:**

1. Appraise the current level of understanding of the pathophysiology of vestibulodynia.
2. Compare women with and without vestibulodynia on physical and psychological variables.
3. Create evidence-based physiotherapy rehabilitation treatment protocols for women with vestibulodynia according to recent studies and international guidelines.
4. Understand the impact of physiotherapy rehabilitation on psychosexual, morphological and functional outcomes in women with vestibulodynia.

# Abstract: D009 – LEVATOR ANI AVULSION INJURY: CAN WE 'FEEL' WITH OUR FINGERS AS WELL AS WE'SEE' WITH ULTRASOUND?

**Published:** 2011-07

**Authors:** C Dumoulin

**Affiliations:** École de réadaptation, Faculté de médecine, Université de Montréal

**Background:** Physiotherapists trained in pelvic-floor rehabilitation evaluate pelvic floor muscle function using intra-vaginal manual palpation in the evaluation and treatment of pelvic floor dysfunction. It is becoming increasingly recognized that trauma to the levator ani (LA) (‘avulsion’ injury) is significantly associated with pelvic floor muscle dysfunction (1). Magnetic resonance imaging (MRI) and pelvic floor ultrasound (US) can visualize avulsion injuries whereas manual palpation appears to require substantial training (2). Despite ultrasound being generally more accessible, it is still not available in all clinical settings and is operator dependent, increasing the need for a reliable digital means of assessment. When correlated to US findings, the predictive value of four manual palpation parameters was confirmed. The level of evidence is still limited therefor more research is needed in different patient populations as results are limited to women with urinary incontinence. Knowledge of proper evidence-based avulsion will be easily achieved by demonstrations and will impact current practice effectively.

(1) Dietz, HP. The aeitology of prolapse. Int Urogyne J Pelvic Floor dysfunction. 2008 19(10) 1323-9

(2) Dietz, HP., Shek, C. Validity and reproducibility of the digital detection of levator trauma. Int Urogynecol J Pelvic Floor dysfunction. 2008 19 1097-1101

**Relevance to Physiotherapy Practice:** Recent randomized control trials and systematic reviews on the management of pelvic floor dysfunctions have established the efficacy of physiotherapy intervention. However, effective intervention relies heavily on appropriate assessment. Proper manual palpation provides physiotherapist with a reliable cost-effective accessible assessment of pelvic floor muscle avulsions.

**Target Audience:** This session is directed towards physiotherapists trained in pelvic-floor rehabilitation with expertise in evaluating pelvic floor muscle function using intra-vaginal manual palpation. Those with without expertise but with interest in the assessment of the pelvic floor muscles in women are also welcomed. Basic understanding of pelvic floor anatomy is suggested.

**Summary of Supporting Evidence:** Levator ani muscle trauma is a common consequence of vaginal childbirth with a prevalence of 15-25% and is commonly associated with organ prolapse, anal sphincter tears and fecal incontinence (Deitz & Shek, 2008). While imaging, both by ultrasound and magnetic resonance, can reliably detect such defects, access to both technologies (and the skills to utilise such technology) is limited.

**Description of Session Format:** Lecture format

Fours techniques will be reviewed: 1. direct palpation of a discontinuity of puborectalis where the muscle attaches to the pubic ramus. 2. Palpation of the distance between the two muscle insertion sites, 3. Palpation of LA strength, and 4. Palpation of LA tone. US images and video clips will facilitate understanding and visualization of pelvic floor anatomy and demonstrations on a 3D pelvic floor model of the different manual palpation techniques will ensure that assessment skills are reproducible. A 10 minute question and answer period will allow the audience to clarify key concepts.

**Session Objectives:**

1. Define pelvic floor muscle avulsions and their role in pelvic floor dysfunction.
2. Visualize and recognize pelvic floor muscle avulsions using US images.
3. Demonstrate how to assess pelvic floor muscle avulsions in women using a reliable manual palpation approach.

# Abstract: D010 – THE 5 DIMENSIONS OF WELL BEING: MAXIMIZING YOUR PERSONAL IMPACT WITH PATIENTS

**Published:** 2011-07

**Authors:** T Yardley, W Coombs, E Trimble

**Affiliations:** * CBI Health Group, British Columbia, Canada; † PT Health, Alberta, Canada; ‡ Prosperous Physical Therapy, Vancouver

**Background:** Working conditions in the healthcare sector are a worldwide problem (Yassi et al, 2002). Studies have shown that healthcare workers' physical and mental well-being is at high risk due to "on" and "off" work demands with 45 % of them reporting most days as being "quite" or "extremly" stressful.

Turbulent times in healthcare require the development a sound knowledge basis of the components of well being not only for patients but also for health professional themselves. Well-being is one measure of the quality of life of an individual and of a society (Diener, Shigehiro & Lucas, 2003). Career, Social, Financial, Physical and Community Wellbeing have been identified the 5 elements of achieving that status.

The aim of this presentation is to expand the knowledge of these topic areas into the realms of behavioral science, economics, neurobiology and epidemiology to better understand the patient's rehabilitation experience and to facilitate self-reflection amongst clinicians on their and work-life balance.

**Relevance to Physiotherapy Practice:** Rehabilitation has been criticized for its preoccupation with the physical aspects of care. Within a patient-centered approach, a holistic acknowledgement of the individual is crucial. Patient-centered practice has been associated with improved patient care and increased efficiency in care. Stress has been shown to impact communication, interpersonal effectiveness and empathy, skills required to establish patient-therapist rapport, a requirement for patient-centered care. Deepening self-awareness amongst physiotherapist about their own well-being and providing strategies to influence it may positively impact patient-centered practice, therapeutic outcomes and overall quality of care.

**Target Audience:** This session will be of interest to a broad range of physiotherapy professionals including clinic owners, managers, professional leaders, clinicians, educators, and students who are interesting in gaining knowledge about wellbeing and increase their awareness and understanding of the impact of each dimension on health status and quality of life. Prior exposure to the topic area is not required.

**Summary of Supporting Evidence:** Although the science of well-being is in its infant stages, it is rapidly evolving (Huppert & Baylis, 2004). The concept of well-being encompasses positive physical, psychological and social states. Animal studies first highlighted the importance of mother -infant bond and the development of future trusting loving relationship in the development of well-being. From a neurobiological perspective, these studies showed a permanent increase in glucocoticoid receptors in the hippocampus and prefrontal cortex resulting from high levels of affectionate behaviors in rats (Meaney, 2001). The brain patterns have also been examined in humans; individuals with more positive and optimistic attitudes exhibit greater activity in the left pre-frontal cortex. High levels of activity in the left pre-cortex were also associated with advantageous profile of hormonal responses to stress, including reduced durations of cardiovascular responses to the stress, and high levels if immune function (Davidson, 2004). Research in clinical epidemiology established the effects of intra-uterine environment and childhood development on physical and mental health later in life (Barker 2004). These results suggest that individuals who experience frequent positive emotion maintain better levels of health than those who do not. A reciprocal relationship between physical health and well-being has been theorized in the literature however has yet to be demonstrated experimentally (Huppert & Baylis, 2004).Research in economics and politics have focused on the relationship between our social capital and well-being; findings suggest that those individuals who have trust in others tend to have highest level of both happiness and life satisfaction (Helliwell & Putman). Positive emotions have also been demonstrated to fuel an urge to play, thus encouraging imagination and enhance social relationships (Frederickson, 2004).

The key to well-being is the balance between appropriate positive and negative emotions. Based on this evidence, a variety of strategies to address well-being have been examined in the literature and focus primarily on the nurturing of positive emotions, attitudes and behaviors. Integration of these strategies at both the individual and public policy level has been proposed.

**Description of Session Format:** This session will be in lecture format and will include a question and answer period at the end of the presentation that will provide participants the opportunity to share their perspectives and experiences on the topic.

**Session Objectives:**

1. Acknowledge the interplay between neurobiology, psychology and social science in the concept of “well-being”
2. Identify the 5 elements of well-being
3. Communicate at least 3 strategies to enhance well-being in each of the 5 elements
4. Develop leadership skills to increase well-being within the workplace

# Abstract: D011 – WE CAN IMPROVE PATIENT ADHERENCE TO PHYSIOTHERAPY RECOMMENDED TREATMENT

**Published:** 2011-07

**Authors:** W Coombs, D Purcell

**Affiliations:** * PT Health, Alberta; † PT Health, Nova Scotia

**Background:** Issues surrounding poor adherence to treatment and non-compliance have been identified across many health care disciplines including physiotherapy. In physiotherapy, intervention effectiveness and achievement of outcomes are highly dependent on patient adherence to treatment. The role of the patient experience in adherence cannot be underestimated. Effective provider-patient communication and accessible environments have been shown to improve the patient experience, increase patient satisfaction and adherence (Potter et al, 2003). Due to the multifactorial nature of non-adherence, successful physiotherapists need to be comfortable and confident in discussing any issues that patients may raise. Additional training in communication skills, behavior modification, and the identification of organizational factors which act as barriers to adherence has been identified by physiotherapists as essential in developing better strategies to overcome non-adherence (Potter et al, 2003).

**Relevance to Physiotherapy Practice:** The cost of non-adherence to treatment of persons with chronic disease is astounding. Funding models continue to be powerful determinants of the frequency and duration of physiotherapy care and are highly influenced by achievement of outcome. With the risk of physiotherapy being deemed ineffective as a consequence of non-adherence, physiotherapists need to develop strategies to improve adherence as a component of effective physiotherapy.

**Target Audience:** This session will be of interest to a broad range of physiotherapy professionals including clinic owners, managers, professional leaders, clinicians, educators and students who are interested in improving patient adherence.

**Summary of Supporting Evidence:** The importance of physiotherapists adopting patient-centered approaches and developing effective communication skills to optimize the physiotherapist interaction is well supported in the literature. Patient most often attribute good experiences to effective communication ability, followed by high quality service provided by the physiotherapists (Liddle, Baxter & Gracey, 2007; Potter et al, 2003). Important communication attributes of a physiotherapist from the patients' perspective relate to the physiotherapists' interpersonal skills, manner and teaching ability (Potter et al, 2003). In addition, physiotherapists are also expected to be organized and demonstrate appropriate professional behavior while providing services in a welcoming and easily accessible environment (Potter et al, 2003).

Interventions that combine cognitive behavioral approaches with the management of practical and organizational patient barriers have been suggested in the literature (Burton, Bradley and Littlewood, 2010; Jack et al, 2009) however have yet to be studied. Moderate evidence exists supporting motivational cognitive behavioral programs are effective in improving short-term adherence to treatment and attendance to clinical sessions (Burton, Bradley and Littlewood, 2010). Treatment goal setting is the most commonly used cognitive-behavioral technique in rehabilitation and has been reported to be positively associated with adherence (Scherzer et al, 2001). However, goal setting, particularly where patients' views are elicited and incorporated, involves significantly evolved communication skills (Parry, 2008)

Communication has been described as the most important aspect of practice that health professionals have to master and an essential requirement underpinning any successful encounter. Communication training interventions aimed at improving clinical communication performance have been well studied in medicine and in nursing but not in physiotherapy. Positive effects include changes in attitude, behaviors, quality of care and patient satisfaction (Schultz, Wellard & Swerissen, 2008). However, evidence indicates that for training to be effective, learners need to be ready and motivated for change and training (Schultz, Wellard & Swerissen, 2008, 2008).

**Description of Session Format:** This session will be delivered in lecture format with time set aside for participants to practice various strategies to improve patient adherence through role play. During the discussion period, the speakers will encourage participants to share their challenges and successes in improving patient adherence to treatment.

**Session Objectives:** 1) To discuss how clinical outcomes are related to patient adherence to the recommended treatment plan. 2) To provide communication strategies, systems, and processes for clinicians and support staff to be able to influence patient adherence. 3) To discuss training platforms for staff that can be implemented by management to create consistency in the patient experience regarding these strategies.

# Abstract: D012 – SOCIAL MEDIA GONE WRONG

**Published:** 2011-07

**Background:** Social network sites are a web-based service that allow individuals to 1) construct a public or semi-public profile within a bounded system; 2) articulate a list of other users within whom they share a connection, and 3) view and traverse their list of connections and those made by others within the system (Boyd & Ellison, 2008). They are a widely used networking tool and are fast becoming an indispensable business commodity. However, the rules and boundaries around social, personal and business information shared on these sites are not well defined or understood. The lack of understanding of the personal responsibility surrounding the utilization of these sites and the negative consequences that can result from inappropriate network content have raised significant concerns in many health care groups about the appropriate use of such tools by professionals.

**Relevance to Physiotherapy Practice:** The healthcare sector is not immune to the societal trends and fads that impact other fields; they also influence healthcare and subsequently physiotherapy. As physiotherapists in both the public and private sectors learn to utilize social media to promote their business, provide patient education and promote healthy living activities, a need also exists to increase the awareness of the risks associated with utilizing social media and network sites.

**Target Audience:** Public and private physiotherapy business managers, employees/contractors, physiotherapist, physiotherapists' assistants and students who use social network sites.

**Summary of Supporting Evidence:** The use of Internet social networking sites has exploded in popularity as a means for individuals to post information about themselves and communicate with others. By late 2009, the most popular network site, Facebook, reported 200 million active users world-wide (Nelson, Simek & Foltin, 2009). Its popularity has been fuelled by the perception that it is somewhat of a private forum with access limited only to those enrolled. Users of the site have therefore felt relatively free to post personal information about themselves and their social network. Evidence suggests that social network users are, by in large, quite oblivious and unconcerned about their personal privacy with the scope of network users exposing themselves to various physical, cyber and security risks, and make it extremely easy for third parties to create digital dossiers of their behaviours (Gross & Acquisti, 2005). Studies have reported that 20% of users indicate that they had content on their social networking page that they would not want current or prospective employers to see suggesting that users of social network sites are somewhat naive about the potential negative consequences concerning the access and use of the information on these websites (Peluchette & Karl, 2007). Seventy -five percent of employers are reportedly becoming increasingly aware of these sites and are taking advantage of the massive amount of newly available information to assist them in their hiring and dismissal decisions (Byrnside, 2008). In addition to utilizing these sites for background checks, many businesses have also embraced the use of social networking as a way of doing business, creating new avenues to exchange information, recruit, market goods and services and collaborate. Many sites require the creation of a personal profile and the upload of business data. Information posted on these sites can lead to organizational security risks such as exposure of corporate data to the public or workplace exposure of individuals' private information (Williams et al, 2007). Finally, cyberbullying, initially reported in children and adolescent social networking, is quickly proliferating to the workplace. In a recent survey, 11% of respondents reported cyberbullying in the workplace (Priviter and Campbell, 2009).

Although the appropriateness of information on social networks and its use by various parties has been questioned, it poses unique challenges and it is not clear how legislation applies. The development of social media use workplace policies are therefore essential in setting workplace expectations with regards to use of social networks both on and off the job.

**Description of Session Format:** Lecture with role playing scenarios and audience participation.

**Session Objectives:** This workshop will explore the significant risk factors for employer and employee/contractors using social media both from the business and personal perspective.

On completion of the session all participants will

1. Recognize their professional responsibility when engaging with social media websites
2. Understand what needs to be considered when utilizing this business tool
3. Consider some of the potential legal, legislative and cultural implications of social media. All participants will be more aware of the impact that social networking can have on the workplace relationships and be more prepared to develop appropriate workplace policies about social media and its use.

# Abstract: D013 – WHIPLASH: 'MINOR' INJURY BUT COMPLEX CONDITION

**Published:** 2011-07

**Authors:** M Sterling

**Affiliations:** University of Queensland, Aus

**Background:** Patients presenting with whiplash-associated disorders are a common entity in musculoskeletal clinical practice. Such presentations can be very complex in nature and require accurate diagnosis and a comprehensive bio-psycho-social approach to management. In recent years the evidence for the physical and psychological manifestations of WAD has significantly increased. These include sensory disturbances indicative of augmented central nociceptive processing, motor and sensori-motor changes. In the psychological domain, posttraumatic stress symptoms play an important role. It is important that physiotherapists can assess, interpret assessment findings and integrate these to the most optimal management approaches for WAD.

It is clear that current treatment strategies are not successful in reducing the transition to chronicity following injury. Why is this case? The aim of this presentation is to equip physiotherapists with a new understanding of the whiplash condition that includes all aspects of this condition. Participants will learn how to integrate this knowledge into more targeted treatment approaches that go beyond the standard motor control approaches to this condition.

**Relevance to Physiotherapy Practice:** Whiplash is a recalcitrant condition to standard physiotherapy approaches to treatment. It is important that physiotherapists can recognize those patients at risk of poor recovery and/or at risk of non-responsiveness to treatment. The research evidence also suggests that some patients, especially those with poor outcomes demonstrate a complex clinical picture that will likely require an integrated approach to management from several providers. The physiotherapist, by virtue of our assessment skills, will play a unique and important role in the integration of patient care.

This presentation will provide physiotherapists with an introduction to the skills required to assess the patient with whiplash, taking into account all aspects of the condition both physical and psychological. This lays the foundation for enhanced management and improved patient outcomes.

**Target Audience:** This presentation is aimed at graduate physiotherapists who are involved in the management of patients with WAD.

**Summary of Supporting Evidence:** It is becoming clear that whiplash is a heterogeneous condition with sub-groups of patients able to be identified based on varying physical and psychological presentations (Sterling and Kenardy, 2008). Whilst the presence of motor dysfunction occurs almost universally in all those with neck pain (Jull et al., 2004; Sterling et al., 2003b), sensory disturbances and psychological factors differentiate those with higher levels of pain and disability (Sterling et al., 2010; Sterling et al., 2003a). These whiplash injured people have a more complex presentation involving widespread sensory mechanical and thermal hyperalgesia, occurring both local and remote to the site of injury, and symptoms of posttraumatic stress. Both these factors are strong predictors of poor functional recovery following whiplash injury (Sterling et al., 2006; Sterling et al., 2005). Sensory hypersensitivity has been shown to occur independently of psychological distress and likely reflects biological phenomena involving augmented central pain processing (Sterling et al., 2008). Further investigation of these phenomena has shown that chronic WAD participants with sensory hypersensitivity also demonstrate hypoaesthesia to vibration, thermal and electrical stimuli suggesting a minor peripheral nerve injury as a possible contributor to whiplash pain (Chien et al., 2010). Furthermore the presence of some of these factors (particularly mechanical and cold allodynia) in patients with a chronic whiplash condition mitigated the successful effects of multimodal physical treatment shown in patients without these features (Jull et al., 2007). Musculoskeletal clinicians play an important role in the early assessment and management of the whiplash injured. As such the early assessment and identification of features associated with both poor and good recovery is necessary for both targeted intervention strategies and/or appropriate referral. Most of these factors have been identified in a laboratory centre and it is essential that the findings are translated into clinical practice. Research has commenced on the development of time-efficient tools for use in clinical practice and these will be discussed and demonstrated. The quest for prevention of the transition to chronicity remains with improved early diagnosis and classification.

**Description of Session Format:** 45 Min Lecture with 15 min question period for audience participation.

**Session Objectives:** Learning Outcomes, at the completion of this presentation, participants should:

1. Understand the clinical assessment and management of whiplash in terms of sensory assessment to identify potential underlying pain processing mechanisms.
2. Understand the psychological assessment of whiplash and the role physiotherapists play in this assessment
3. Have knowledge of the current evidence base for the management of whiplash injuries
4. Be able to integrate sensory and psychological findings into their overall management of the patient with whiplash
5. Have knowledge of appropriate outcome measures for the evaluation of whiplash
6. Be able to understand the role physiotherapists play in co-ordinating care of the whiplash injured person

# Abstract: D014 – OVERTRAINING SYNDROME IN SPORT: A PHYSIOTHERAPIST'S ROLE

**Published:** 2011-07

**Authors:** D Stacy

**Affiliations:** Hockey Canada

**Background:** Overtraining syndrome (OTS) is a condition in sport that is extremely misunderstood, misdiagnosed and mistreated. It is, however, something that can be avoided. As a sport physiotherapist working on the front lines, you must be aware of how this condition presents itself and how it can be avoided. Research in this area is limited due to the nature of the syndrome, but evidence still exists to guide physiotherapists in proper management.

**Relevance to Physiotherapy Practice:** Overtraining syndrome in sport may be encountered by many physiotherapists who work with athletes. A summary of the nature of overtraining syndrome, risk factors, signs and symptoms, factors that may be involved in the development of overtraining syndrome, assessment tools, management and prevention strategies will be discussed in this session. An understanding of signs and symptoms, risk factors and a strategy for prevention and management will enable the treating physiotherapist to recognize this syndrome and facilitate appropriate treatment strategies.

**Target Audience:** This course is designed for physiotherapists with clinical practice in the areas of sport, orthopaedics and neurological physiotherapy. There is no prerequisite for this course and it is applicable to therapists of all levels of experience.

**Summary of Supporting Evidence:** There is limited data available; therefore the presentation is largely based on theory. No single metabolic process accounts for all signs and symptoms associated with OTS and no single definitive diagnostic tool. Some references used to support the presentation include: Achten J, and AE Jeukendrup. Heart Rate Monitoring: Applications and Limitations. Sports Med. 33(7): 517-538. 2003.; Armstrong, LE, and JL Vanheest. The unknown mechanism of the overtraining syndrome. Clues from depression and psychoneuroimmunology. Sports Med. 32: 185-209. 2002.; Baumert M, Brechtel L, Lock J, Hermsdorf M, Wolff R, Baier V, and Voss A. Heart Rate Variability, Blood Pressure Variability, and Baroreflex Sensitivity in Overtrained Athletes. Clin J Sports Med. 16(5): 412-417, 2006.; Budgett R, E Newsholme, M Lehmann, C Sharp, D Jones, T Peto, D Collins, R Nerurkar, and P White. Redefining the overtraining syndrome as the unexplained underperformance syndrome. B J Sports Med. 33: 17-18. 1999.; Halson, SL and AE Jeukendrup. Does Overtraining Exist? An analysis of overreaching and overtraining research. Sports Medicine, 34(14): 967-981, 2004.; Meeusen R, Watson P, Hasegawa H, Roelands B, and Piacentini MF. Brain Neurotransmitters in Fatigue and Overtraining. Appl Physiol Nutr Metab. 32: 857-864, 2007.; Urhausen, A, and W Kindermann. Diagnosis of Overtraining: What tools do we have? Sports Med. 32(2): 95-102. 2002.

**Description of Session Format:** The session will be primarily lecture format with some opportunity for group interaction and participation.

**Session Objectives:** The participant will increase their knowledge related to overtraining based on a continuum of evidence from basic science, through clinical and population health. They will also understand the complexity of signs and symptoms associated with overtraining in sport and be able to apply the different theories around the causation and diagnosis of the overtraining syndrome. Participants will examine evidence-based perspectives regarding the treatment and prevention of overtraining in sport.

# Abstract: D015 – SCOPE OF PRACTICE IN THE 2010'S – DIFFERENTIAL DIAGNOSIS, IMAGING AND EXERCISE PRESCRIPTION

**Published:** 2011-07

**Authors:** K Khan, W Becker, L McLaughlin

**Affiliations:** * University of British Columbia, Vancouver; † University of Calgary; ‡ McMaster University, Hamilton, ON

**Background:** Physical Therapy has gradually evolved into a profession with specialized areas of practice, which requires musculoskeletal “expertise” in evaluation and treatment. Numerous studies published in the past decade support the appropriateness, effectiveness, and patient satisfaction related to primary care initiatives involving physiotherapists. In Canada, and internationally, Advanced Practice (AP) initiatives have grown such that new exciting roles are emerging for physiotherapists in multidisciplinary environments. These include triage mechanisms for patients with spinal disorders seeking surgical options where appropriateness and use of diagnostic imaging in physiotherapists practice is required.

Physiotherapists are also required to provide appropriate differential diagnosis in patients with complex conditions. Patients with persistent headache can present with such a multifaceted scenario. Differential diagnosis and identification of headaches that are amenable to physiotherapy management versus those that may require further medical evaluation are essential steps in the effective management of this condition.

Physiotherapists are also expected to maintain an effective treatment role. Exercise prescription is a mainstay of a physiotherapists practice for many musculoskeletal problems. The evidence is overwhelming in support of exercise as part of a multimodal management physiotherapeutic intervention. Physiotherapists are especially trained in exercise prescription and utilize this as a significant part of their patient-centered intervention.

**Relevance to Physiotherapy Practice:** As primary care providers, physiotherapists are at the forefront in managing patients with various musculoskeletal problems including spinal disorders and headaches. In many circumstances, one of the key factors related to positive treatment outcomes is an accurate diagnosis. Complex and functionally disabling clinical problems such as headaches and back pain require a diagnosis based on scientific evidence and international guidelines. Management of such disorders may require physiotherapy intervention and/or they may require medical treatment, rendering accurate diagnosis essential. Advanced physiotherapy practitioners are often faced with clinical scenarios where the patient may require conservative care or surgical management. Sound decisions are necessary for optimal management of such patients. Arguably, both relative simple and complex clinical problems will benefit from interventions related to exercise where physiotherapists are at the forefront of providing patient-specific, evidence-based exercise prescription.

**Target Audience:** This workshop will be of interest to a broad range of physiotherapists including students, clinicians, managers, professional leaders, educators, and researchers interested in maximizing patient outcomes in rehabilitation settings.

**Summary of Supporting Evidence:** There have been numerous studies published in the past decade supporting the appropriateness, effectiveness, and patient satisfaction related to primary care initiatives involving physiotherapists. Importantly, patient safety has been maintained. As part of the primary care initiatives, the appropriateness and use of diagnostic imaging in physiotherapists practice has been described.

The effective management of cervicogenic headache has been reported in randomized clinical trials and includes manual therapy and exercise intervention. International guidelines have been published outlining the clinical criteria for the diagnosis of various types of headache. Based on the type of intervention required, accurate classification of the type of headache is necessary.

There have been many systematic reviews and randomized clinical trials published evaluating the effectiveness of exercise intervention for various musculoskeletal disorders. Transparent research methodology enables clinicians to transcribe the parameters related to the exercise intervention directly into their clinical practice. Application of exercise principles can be provided by clinicians of all levels of expertise making this type of intervention attractive for use in clinical practice.

**Description of Session Format:**

Setting the stage – Description of symposium

Session Chair: Introduction - 5 minutes

A. Advanced Practice and Diagnostic Imaging in PT Practice

Laurie McLaughlin

Objectives:

1. To synthesize information and concepts applied to advanced practice and diagnostic imaging in physiotherapy practice 30 minutes

B. Differential Diagnosis of Headache

Werner Becker

Objectives:

1. To apply evidence-based criteria for the differential diagnosis of headache
2. To identify common patient presentations in scenarios related to headaches 30 minutes

C. Supersize my exercise! Learning from Mad Men, the Marlboro Man and Freakonomics to Promote Physical Activity

Karim Khan

Objectives:

1. To apply the principles of exercise prescription in practice
2. To illustrate the benefit of exercise, both in injury rehabilitation, but in the community as a whole in regard to public health benefits of exercise
3. To demonstrate the important role physiotherapists play in regard to effective exercise prescription 30 minutes

D. Panel Discussion - 30 minutes

**Session Objectives:** To highlight the expanding role of physiotherapy scope of practice in the 2010's.

# Abstract: D016 – THE INFLUENCE OF THE THERAPEUTIC RELATIONSHIP ON PATIENT OUTCOMES

**Published:** 2011-07

**Authors:** T Yardley

**Affiliations:** CBI Health Group, British Columbia

**Background:** Clinicians understand the importance of having a good relationship with our patients from a patient satisfaction standpoint, but do we really understand the impact of this relationship on the treatment outcome? A systematic review summarizing relevant research on the influence of the therapist-patient relationship on treatment outcomes was published in the Physical Therapy Journal in 2010. The results confirmed the need to shift from a biomedical to a biopsychosocial model.

Why is it that the strongest clinicians don't always get the strongest results? It is a source of frustration to many that many of our competitors in the healthcare marketplace rely on gadgets and gimmicks with minimal research and questionable results. Regardless, many of them build a healthy business on this shaky foundation. In this session, we will explore the results of the outcomes research and the notion that the patient's outcome is correlated to a significant degree to numerous factors, of which the treatment technique is only a small part. Biopsychosocial factors such as the patient-therapist alliance, the environment, the beliefs of the PT, the beliefs of the patient and the placebo effect also have a significant effect on the outcome. Some of the research indicates that the relationship actually accounts for 60% of the treatment effect!

When we fail to acknowledge the role and importance of the therapeutic relationship between the clinician and client and the impact that our own beliefs have on the patient's experience and outcome, we miss key pieces of the puzzle. The goal of this session is to explore how we can enhance the patient's experience of rehabilitation, forge a strong, sustainable therapeutic alliance, and build customer engagement.

**Relevance to Physiotherapy Practice:** Most clinicians spend their continuing education resources on the acquisition of additional clinical skills, techniques and equipment, often with little change in patient adherence, engagement or outcomes. In this session we will explore factors influencing patient adherence, learn how to measure patient engagement, and determine relevant outcome measures that pertain to the building of a positive therapeutic relationship.

**Target Audience:** A prior level of exposure to this topic is not required to successfully follow, enjoy and take away valuable information and skills from this session. This session is targeted to any clinicians in healthcare including private practice clinic owners, public and private administrators, employees, and contractors.

**Summary of Supporting Evidence:** Supporting evidence will be drawn from the following sources:

Physical Therapy Journal - The influence of the therapist-patient relationship on treatment outcome in physical rehabilitation: a systematic review. (2010)

This comprehensive systematic review explores the relationship between the therapist-patient alliance and results indicate that it appears to have a positive effect on treatment outcomes in physical rehabilitation settings.

Spine Journal – Physical Therapist Pain Beliefs and Their Influence on the Management of Patients with Chronic Low Back Pain (2004)

The authors of this qualitative study explored physiotherapist's pain beliefs and how their beliefs affected their management of patients with chronic low back pain. They also explored the concept that the therapeutic encounter is a meeting of two belief systems and that patients and physiotherapists may occasionally act at cross-purposes. Despite overwhelming literature in favour of a biopsychosocial approach, treatments were biomedically oriented, even when psychological factors were apparent in patients' presentation. This contributed to PT's feeling frustrated, disheartened, and less sympathetic, which related to patients having a sense of poor self-efficacy and outcome expectancy beliefs, and ultimately a less-than-favourable outcome.

This research will be coupled with research from the behavioural sciences and economics fields to make the link with a patient's experience and the level of patient satisfaction/engagement and to identify key areas in which we might impact a patient's outcomes (Economic Journal – Lags and Leads in Life Satisfaction: a Test of the Baseline Hypothesis – 2008; Psychoneuroendicrinology – Perceived stress and cortisol levels predict the speed of wound healing in healthy adult males – 2004; BMJ – Dynamic Spread of Happiness in a large social network; Longitudinal analysis over 20 years in the Framingham heart study – 2008)

**Description of Session Format:** This session will be in an interactive lecture format that includes analysis of a case study to promote experiential learning and critical thinking and will include a question and answer period at the end of the presentation to provide participants the opportunity to share their perspectives and experiences on the topic.

**Session Objectives:** Upon completion of this session, participants will be able to:

1. Access relevant literature on the critically important subject of relationship-building
2. Identify and employ strategies to build a stronger and more meaningful patient-therapist alliance (communication skill development)
3. Measure the impact of their actions on patient engagement and adherence through use of biopsychosocial outcome measures as opposed to biomedical indicators alone

# Abstract: D017 – PLACEBO EFFECTS AND ACTIVATION DURING TREATMENT AND PHYSIOTHERAPY INTERACTION

**Published:** 2011-07

**Authors:** B Shay

**Affiliations:** Department of Physical Therapy, University of Manitoba, Winnipeg, MB

**Background:** The neurophysiological basis for placebo effects has been established in many different physiotherapy treatment interventions. A growing body of evidence related to the role of patients' expectations and anticipation in enhancing or reducing the pain experience has led experts in the field to reconsider the effect of the placebo response including the influence of the different aspects of human experience on the magnitude of placebo analgesic response. Exploitation of the placebo mechanism for the benefit of the patient has been a topic of much controversy. As evidence-based practitioners, physiotherapists need to understand this placebo mechanism, its role in their treatment interventions and how it is elicited during various therapeutic interactions. A lack of understanding in the relationship between acupuncture and placebo has been identified as a barrier to advancing research in this field. The purpose of the session is to provide a review of the current literature on placebo effects with an emphasis on the literature surrounding acupuncture achieved analgesia.

**Relevance to Physiotherapy Practice:** As physiotherapists, we should understand the placebo effects linked to our interventions and take advantage of this response in achieving analgesia for our patients. In addition, this knowledge is also key in carefully avoiding adverse reactions, such as a nocebo response, the placebo mechanism's evil twin (Reid 2002).

**Target Audience:** Physiotherapists and students who treat patients with pain in their practice will find this session relevant. An entry-level to practice background knowledge of pain theory would be useful, however, current physiological theories in pain modulation will be reviewed. No prior knowledge of acupuncture is required.

**Summary of Supporting Evidence:** It is clear that expectation is a potent factor in the relief of pain. Evidence continues to emerge shifting the idea that placebo effects are the result of inert agents given to patients, to the idea that placebo effects result from the expectations, beliefs and desires of patients and vary in magnitude as a function of these variables (Price et al, 1999; Price & Barrell, 2000; Price et al 2007). The recent introduction of brain imaging tools has provided scientists in a variety of areas with the capacity to document brain activity and the effects of placebo analgesia (Kong et al, 2005). This has given way to an increasing body of evidence in the field of psychology, pharmacology and neuroimaging supporting placebo analgesia (Price 1999b, Price and Bushnell 2004, Tracey 2010).

Results from psychological studies have shown that the perception of the placebo agent is central to the magnitude of the placebo analgesic effect (Price 1999b, Vase, Riley, & Price, 2002, Price and Bushnell 2004, Tracey 2010) thus highlighting the importance of participants' knowledge and understanding of the therapeutic intervention in maximizing its effect (Vase, Riley & Price, 2002). Pharmacological evidence has suggested that the perception and expectation of the placebo agent trigger the descending opioid inhibitory pathways which can modulate the processing of pain, resulting in not only reducing pain intensity but also in reducing the pathophysiological consequences of pain. These pathways have also been shown to be activated during physical interventions commonly used by physiotherapists such as acupuncture, transcutaneous electrical nerve stimulation (TENS) and exercise. Neuroimaging research has also highlighted the activation of these similar pathways during both the placebo and nocebo conditions (Sackett 2009; Schweinhardt and Bushnell, 2010; Tracey 2010) and have identified different brain activation patterns during different circumstance and with different modalities (Kong et al, 2005).

It is clear that the placebo analgesic effect is multimodal and the identification and understanding of the factors that contribute to it are vital in optimizing the general effectiveness of pain treatments and maximizing the analgesic effect of physical interventions.

**Description of Session Format:** The session is a combination of standard presentation with interactive audience contribution. Case studies will be used to assist in knowledge integration.

**Session Objectives:**

1. To introduce the current literature exploring the placebo and treatment
2. To explore the link between expectation and the relief of pain in terms of placebo analgesia
3. To learn how to exploit the effects of placebo in their treatment and not create a negative effect or nocebo.
